# Unique gene duplications and conserved microsynteny potentially associated with resistance to wood decay in the Lauraceae

**DOI:** 10.3389/fpls.2023.1122549

**Published:** 2023-03-08

**Authors:** Xue-Chan Tian, Jing-Fang Guo, Xue-Mei Yan, Tian-Le Shi, Shuai Nie, Shi-Wei Zhao, Yu-Tao Bao, Zhi-Chao Li, Lei Kong, Guang-Ju Su, Jian-Feng Mao, Jinxing Lin

**Affiliations:** ^1^ National Engineering Research Center of Tree Breeding and Ecological Restoration, State Key Laboratory of Tree Genetics and Breeding, Key Laboratory of Genetics and Breeding in Forest Trees and Ornamental Plants, Ministry of Education, College of Biological Sciences and Technology, Beijing Forestry University, Beijing, China; ^2^ National Tree Breeding Station for Nanmu in Zhuxi, Forest Farm of Zhuxi County, Hubei, China; ^3^ Department of Plant Physiology, Umeå Plant Science Centre, Umeå University, Umeå, Sweden

**Keywords:** Lauraceae, *Lindera megaphylla*, wood decay resistance (WDR), tandem and proximal duplications (TD/PD), gene microsynteny

## Abstract

Wood decay resistance (WDR) is marking the value of wood utilization. Many trees of the Lauraceae have exceptional WDR, as evidenced by their use in ancient royal palace buildings in China. However, the genetics of WDR remain elusive. Here, through comparative genomics, we revealed the unique characteristics related to the high WDR in Lauraceae trees. We present a 1.27-Gb chromosome-level assembly for *Lindera megaphylla* (Lauraceae). Comparative genomics integrating major groups of angiosperm revealed Lauraceae species have extensively shared gene microsynteny associated with the biosynthesis of specialized metabolites such as isoquinoline alkaloids, flavonoid, lignins and terpenoid, which play significant roles in WDR. In Lauraceae genomes, tandem and proximal duplications (TD/PD) significantly expanded the coding space of key enzymes of biosynthesis pathways related to WDR, which may enhance the decay resistance of wood by increasing the accumulation of these compounds. Among Lauraceae species, genes of WDR-related biosynthesis pathways showed remarkable expansion by TD/PD and conveyed unique and conserved motifs in their promoter and protein sequences, suggesting conserved gene collinearity, gene expansion and gene regulation supporting the high WDR. Our study thus reveals genomic profiles related to biochemical transitions among major plant groups and the genomic basis of WDR in the Lauraceae.

## Introduction

Wood is an exceptionally useful biomaterial, with myriad uses in construction, pulp and paper, and as a biofuel. Moreover, wood is a renewable material. One problem with using wood as a renewable biomaterial is that many microbes and insects have evolved to use wood as an energy source, producing enzymes that break down the components of the wood. Some species have evolved mechanisms to resist microbial damage and oxidation; many species with high wood decay resistance (WDR), such as teak (*Tectona grandis*), redwood (*Sequoia sempervirens*), and mahogany (*Swietenia mahagoni*) are rare and extremely valuable. Therefore, understanding the genetic basis and molecular mechanisms of WDR has the potential to provide effective information for improving WDR in commercially grown tree species. Wood is mainly composed of cellulose, hemicellulose, and lignin, which provide structural support for trees and resistance to microbial attack (Nascimento et al., 2013). Generally, lignin, a phenolic compound that is extremely resistant to degradation by certain fungi and plant diseases, acts as the basal component of wood durability by covering and protecting cellulose (Vance et al., 1980; Mounguengui et al., 2016). Further, trees resistant to decay exhibit significant production or accumulation of some bioactive compounds that function as antifungal compounds, antioxidants, or insect antifeedants, and are the main factors contributing to WDR (Nascimento et al., 2013). WDR is influenced by alkaloids such as indols and beta-carboline alkaloids, which have strong antifungal activity, as well as berberine and palmatine, which have shown good antifeedant and antioxidant activities (Kawaguchi et al., 1989; Anouhe et al., 2018; Ekeuku et al., 2020; Imenshahidi and Hosseinzadeh, 2020). Moreover, flavonoids are phenolic compounds with strong fungicidal activity, natural antioxidants and are excellent free radical scavengers, which have a significant effect on improving WDR (Schultz and Nicholas, 2000). In addition, terpenoids, including triterpenoids, diterpenoids, sesquiterpenoids, and monoterpenoids, have important antifungal, antifeeding, and antioxidant abilities, and contribute greatly to WDR (Park et al., 2000; Isman, 2002).

Lauraceae, a family of the order Laurales in the Magnoliids, includes about 67 genera and over 2,500 species (Anouhe et al., 2018). Lauraceae species are economically important, playing important roles in timber production, medicine, spice production, and ecological afforestation (Anouhe et al., 2018). A distinguishing feature of most Lauraceae species is the extremely high decay resistance of wood, including resistance to fungi, insect erosion, and oxidation (Jagels et al., 2005). Nanmu species, a group of tree species belonging to the Lauraceae family, are characterized by their straight trunks, fragrant and dense wood, and most notably by their super WDR (Jiao et al., 2022). Given these valuable traits, Nanmu wood is a precious natural resource that has historically been exploited, for example, for the construction of royal palaces (Xie et al., 2015). Generally, most species of the *Phoebe* and *Machilu* genera are recognized as Nanmu (e.g., *Phoebe zhennan* and *Machilu nanmu*) (Jiao et al., 2022). Another tree, *Lindera megaphylla*, has all superior qualities of the generally accepted Nanmu species, and was extensively used for the construction of royal buildings in Beijing in the Qing dynasty (**Figure S1**). *L. megaphylla* accumulates a variety of alkaloids (Chou et al., 1994) that promote resistance to microbial infection and herbivore attack, increasing the antifeeding and antioxidant activities of its wood (Kawaguchi et al., 1989; Ekeuku et al., 2020). *L. megaphylla* also has a wide range of medicinal properties due to alkaloid accumulation (Cao et al., 2016). In addition, the wood of some other Lauraceae species, e.g., *Cinnamomum* (Zhou et al., 2019) and *Litsea* species, have good natural durability and are highly valuable in construction, furniture, sculpture, and other building applications. With the development of society, there is increasing demand for naturally durable wood. However, genetic studies on the natural durability of wood, especially of Lauraceae species, are limited. Therefore, it is of great significance to identify the genes of biosynthetic pathways related to WDR, to investigate whether the WDR-related gene families have expanded significantly, and to reveal whether there are unique and conserved characteristics of WDR-related genes in Lauraleae species.

The phylogenetic location of Magnoliids remains to be further clarified. *Lindera megaphylla* belongs to Lauraceae, which together with Canellales, Piperales, and Magnoliales, constitutes the Magnoliids, including 9,000 species (The Angiosperm Phylogeny Group et al., 2016). Although multiple genomes of Magnoliids have been published, the relationship between magnoliids, eudicots, and monocots remains discordant. For example, the gene sequence-based phylogenomic analyses of *Liriodendron chinense* (Chen et al., 2019), *Piper nigrum* (Hu et al., 2019), *Persea americana* (Rendon-Anaya et al., 2019) and *Phoebe bournei* (Chen et al., 2020a) supported the Magnoliids as sister to the monocots-eudicots clade, while analyses of *Cinnamomum kanehirae* (Chaw et al., 2019a), *Chimonanthus salicifolius* (Lv et al., 2020) and *Chimonanthus praecox* (Shang et al., 2020a) supported Magnoliids as sister clade of eudicots. In addition, the phylogenomic analyses of *Litsea cubeba* suggested that the definite evolutionary relationships between Magnoliids, monocots, and eudicots remains to be resolved due to the possibility of incomplete lineage sorting (ILS) (Chen et al., 2020b). Microsynteny, gene colocality or collinearity, is the local conservation of gene order or gene neighborhood. Microsynteny provides valuable information to infer gene and genome evolution (Bowers et al., 2003; Van de Peer, 2004; Dewey, 2011), and is significant in phylogenetic inferences (Zhao and Schranz, 2019; Zhao et al., 2021c).

Here, we generated a chromosome-level genome assembly of *L. megaphylla* with long-read sequencing and Hi-C scaffolding technologies. The wood of *L. megaphylla* is dense and durable, making it an ideal material for construction, furniture, and shipbuilding. We conducted phylogenomic reconstruction of main angiosperm groups based on multiple strategies of concatenation, coalescent-based, and network-based microsynteny. Further, through the comparative genomics, especially shared gene microsynteny among major angiosperm lineages, we identified unique gene duplications and conserved microsynteny associated with isoquinoline alkaloids (IA), flavonoids, lignin, and terpenoids biosynthesis in Lauraceae species, which may be associated with outstanding wood durability in Lauraceae trees. The genome resources and findings presented here provide a basis for further evolutionary or functional studies in Lauraceae species, and for additional exploration of Lauraceae wood decay resistance.

## Results

### *L. megaphylla* genome sequencing, assembly, and gene annotation

**Figure 1:**
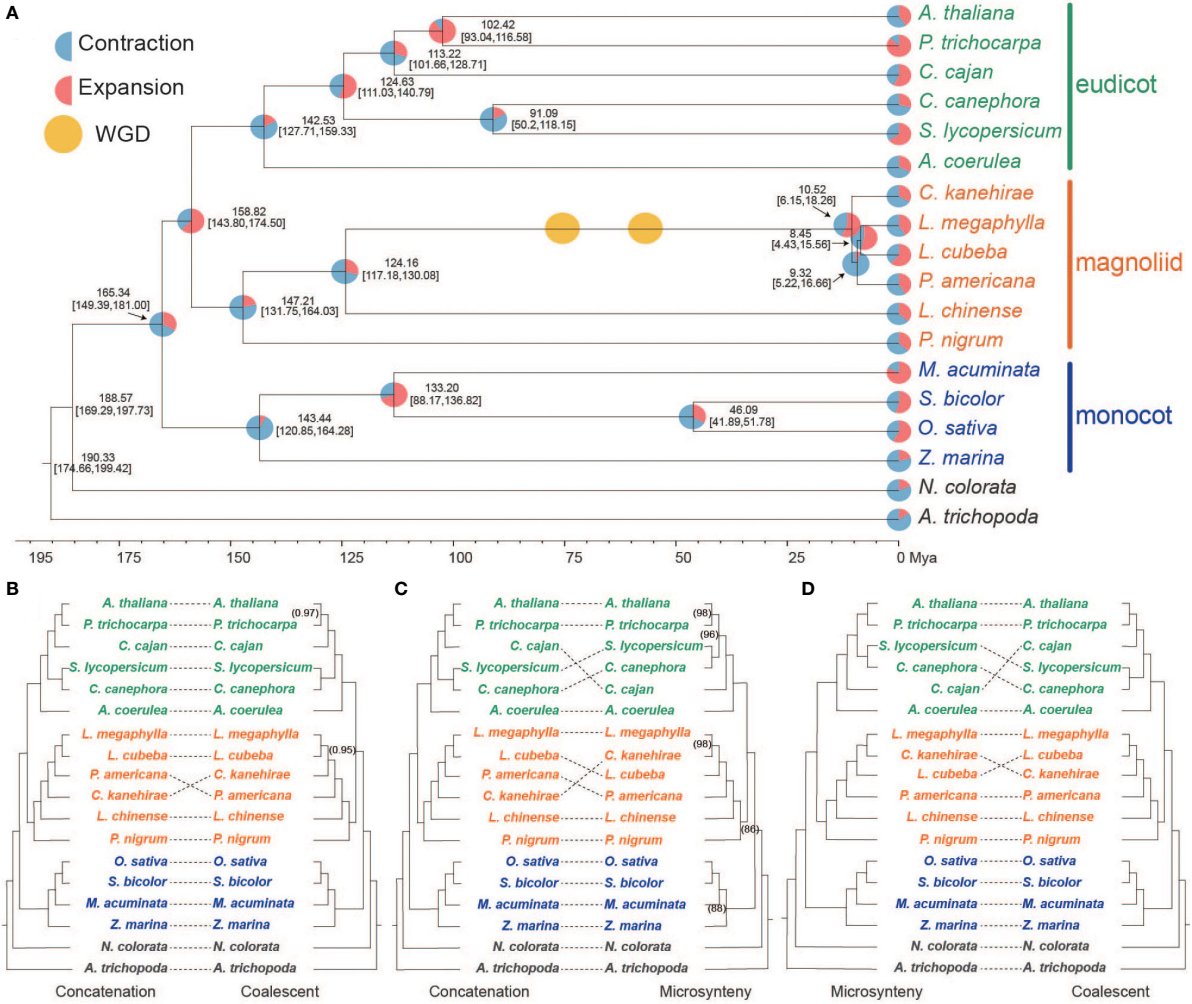
Phylogenomic analysis of three major angiosperm groups. **(A)** Phylogenetic tree of 18 plant species generated by the concatenation-based method. Pie charts indicate the predicted expansion (red) and contraction (blue) of the gene family. The numbers represent divergence time of each node (Mya, million years ago), and values in brackets are 95% confidence intervals for the time of divergence. The yellow circle shows the WGD events identified in Lauraceae species. **(B)** Comparison of phylogenetic trees produced by the concatenation- and multi-species coalescent (MSC)-based methods. **(C)** Comparison of phylogenetic trees generated using concatenation- and microsynteny-based methods. **(D)** Comparison of phylogenetic trees produced using the microsynteny- and MSC-based methods.

As a first step to understand genomics of WDR in Lauraceae species with significant WDR, we sequenced the genome *L. megaphylla*. According to *k*-mer analysis, the genome size of *L. megaphylla* (**Figure S1**) was estimated to be ~1.3 Gb, with a 0.5% heterozygosity rate (**Figure S2** and **Note S1** for details). We generated 178.78 Gb (10.3 million reads, roughly 130× coverage) of Oxford Nanopore Technologies (ONT) long reads (**Table S1**) for primary assembly, 160.28 Gb (1068 million reads, 120× coverage, PCR-free library) of Illumina paired-end reads for correction and polishing, and 223.23 Gb (1488.194 million reads, 170× coverage) of Hi-C paired-end reads for scaffolding (**Figure S3** and **Table S1**). A final genome assembly of 1.27 Gb was obtained, which consisted of 486 scaffolds, including 12 chromosome-level scaffolds, with a scaffold N50 of 104 Mb (**Figure 1**; **Table 1**; **Tables S2**, **3**). The high confidence of the genome assembly was supported by high ten-fold minimum genome coverages of 95.1% (Illumina) and 99.6% (ONT), as well as the high mapping rates of 99.2% (Illumina) and 81.3% (ONT) reads. A 90.7% (1,306 complete genes) Benchmarking Universal Single Copy Orthologs (BUSCO) recovery score (Simão et al., 2015) and a high LTR Assembly Index (LAI) (Ou et al., 2018) score of 12.40 revealed a high completeness in the final assembly (**Table S4**).

**Table 1:** Statistics of the *Lindera megaphylla* genome assembly and annotation.

Sequencing	
Raw bases of WGS-ONT Sequel (Gb)	178.78
Raw bases of WGS-Illumina (Gb)	160.28
Raw bases of Hi-C (Gb)	223.23
Raw bases of mRNAseq (Gb)	144.90
Assembly	
Genome size (Mb)	1,268.60
Number of scaffolds	486
N50 of scaffolds (bp)	104,721,408
L50 of scaffolds	5
Chromosome-scale scaffolds (bp)	1,206,404,078 (95.10%)
Number of contigs	1,407
N50 of contigs (bp)	2,612,587
L50 of contigs	125
Number of Gap	921
BUSCO (genome)	90.70%
GC content of the genome (%)	39.44%
Annotation	
Number of predicted genes	34,216
Number of predicted protein-coding genes	32,586
Average gene length (bp)	7,693.79
Average CDS length (bp)	1,250.89
Average exon per transcript	5.22
Number of tRNAs	579
Number of rRNAs	248
Repeat sequences (bp)	849,656,470 (66.98%)
BUSCO (gene set)	91.70%

A total of 32,586 protein-coding genes were predicted from the final assembly (**Table S5**). The average lengths of total gene regions, transcripts, coding sequences, exons, and introns were 7,693.8, 1,410.1, 1,250.9, 270, and 1,094.7 bp, respectively (**Table S5**). In addition, we annotated 579 tRNAs, 248 rRNAs (including five 28S, six 18S, and 237 5S rRNAs), and 803 other non-coding RNAs (**Table S6**). The strongly supported gene annotation was evidenced by a 91.7% complete BUSCO score, as well as by 85.9% of the predicted genes (29,400 genes) with an annotation edit distance (AED) lower than 0.5 (**Table S4** and **Figure S4**). More results of genome annotation are available in **Note S4** and **Table S7**.

We identified 34,888 gene families, of which 6,340 are shared among all 18 species (**Table S8**) (see “Methods” section for details). And 885 expansion gene families in Lauraceae were enriched in isoquinoline alkaloid biosynthesis, flavonol biosynthesis, phenylpropanoid catabolism, lignin catabolic processes, and sulfur compound transport (**Figure S5**). All of these processes are tightly associated with resistance to bacteria and fungi, insect attacks, and high wood durability. The expanded gene families in *L. megaphylla* were also enriched in isoquinoline alkaloid biosynthesis, positive regulation of flavonoid biosynthesis, and isoflavone 7-O-glucosyltransferase activity (**Figure S6**). Similarly, these processes are all tightly associated with wood decay resistance.

Results of transposable element and other repeat annotation are available in **Note S5**, **Figure S7** and **Tables S9**, **S10**.

### Phylogenetic placement of Magnoliids

To determine the phylogenetic position of the Magnoliids relative to monocots and eudicots, phylogenetic trees were constructed using three distinct methods (concatenation-, coalescent-, and microsynteny-based approaches). For the concatenation-based approach, we constructed a phylogenetic tree using 885 low-copy orthologs from 18 species, with *Amborella trichopoda* and *Nymphaea colorata* as the outgroup (**Figure 1A**) (see “Methods” section). Results showed that the Maximum likelihood (ML) trees placed the Magnoliids as sister to the eudicots (**Figure 1A**). Phylogenetic analysis indicated that divergence time between Magnoliids and eudicots was 158.8 million years ago (Mya), with 95% confidence intervals of 143.8-174.5 Mya (**Figure 1A**), which overlaps with the *C. kanehirae* genome (136-209 Mya) (Chaw et al., 2019a). Lauraceae divergence was 124.16 Mya (**Figure 1A**), which was approximately equal to *Phoebe bournei* (Chen et al., 2020a). In addition, *L. megaphylla* diverged from *C. kanehirae* and *L. cubeba* around 10.52 Mya and 8.45 Mya, respectively (**Figure 1A**).

To reduce the influence of incomplete lineage sorting (ILS) on the determination of phylogenetic position, we also performed coalescent-based analyses of gene trees from the 855 low-copy gene families with ASTRAL-Pro (version 1.1.2) (Zhang et al., 2020a). The result from the coalescent-based analysis with strongly supported topology was highly consistent with the results of the concatenation-based method, placing Magnoliids as a sister group to eudicots after their divergence from monocots (**Figure 1B**). In addition, to reduce the interference caused by gene duplication and loss, ancestor hybridization, and lateral gene transfer in the homology assessment of plants, a novel method for phylogenetic tree reconstruction based on genome-wide synteny network data has been proposed (Zhao and Schranz, 2019; Zhao et al., 2021c). This method, microsynteny or gene order conservation, has been considered to be a valuable and alternative phylogenetic character in addition to sequence-based characters (Zhao et al., 2021c). The microsynteny-based analysis results confirmed that Magnoliids and eudicots are sister groups, which was topologically identical to the results of the above two methods (**Figures 1C, D**). These results strongly support that Magnoliids and eudicots are sister branches of monocots.

### Microsynteny sharing and functional implications

**Figure 2:**
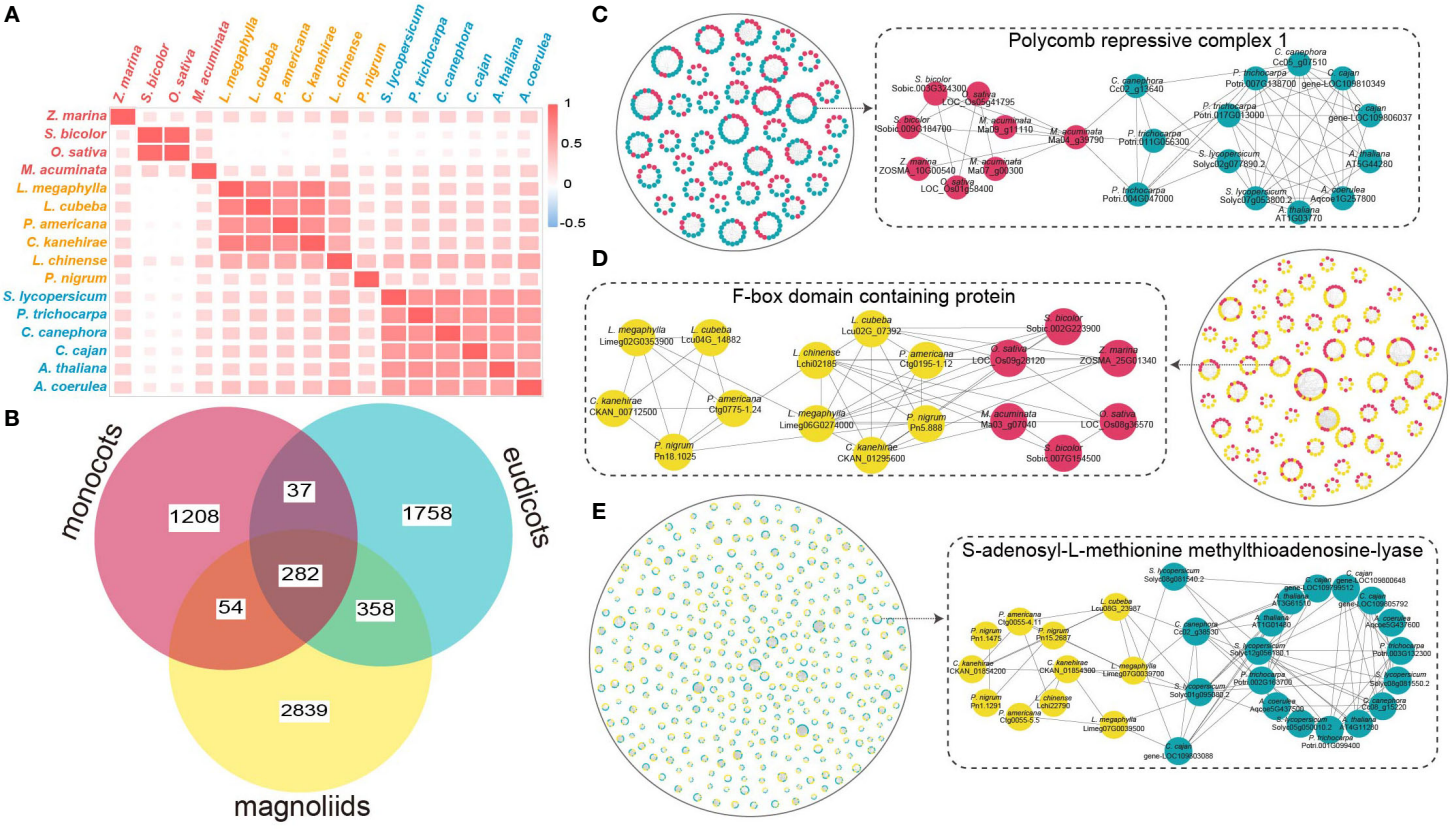
Analysis of gene microsyntenic clusters. **(A)** Heatmap of the number of microsyntenic clusters shared among Magnoliid, eudicot, and monocot species. **(B)** Venn diagram showing the microsyntenic clusters shared among Magnoliids, eudicots, and monocots. **(C)** The black solid circle on the left surrounds microsyntenic clusters shared by eudicots and monocots, where blue dots represent eudicots, and red dots represent monocots. The black dotted rectangle on the right highlights an example of a cluster (in a subnetwork) shared between eudicots and monocots. **(D)** The black solid circle on the right surrounds microsyntenic clusters shared by Magnoliids and monocots, where yellow dots represent Magnoliids and red dots represent monocots. The black dotted rectangle on the left highlights an example of a cluster (in a subnetwork) shared between Magnoliids and monocots. **(E)** The black solid circle on the left surrounds microsyntenic clusters shared by Magnoliids and eudicots, where yellow dots represent Magnoliids and blue dots represent eudicots. The black dotted rectangle on the right highlights an example of a cluster (in a subnetwork) shared between Magnoliids and eudicots.

To examine the lineage-specific microsynteny profile of major plant groups (Magnoliids, monocots, and eudicots), the genome synteny cluster obtained from microsynteny-based analysis of 16 species excluding *N. colorata* and *A. trichopoda* was analyzed. Interestingly, the Lauraceae species *L. megaphylla*, *L. cubeba*, and *C. kanehirae* had the largest number of microsyntenic clusters, with 15,347, 14,879, and 14,830 from each species, respectively (**Figure S8A**). The number of microsyntenic clusters shared by Magnoliids-eudicots (3,840) was significantly more than that shared by Magnoliids-monocots (871) and eudicots-monocots (491) (**Figure S8B**). Based on the heatmap of correlation in shared microsynteny, we observed a strong correlation between Magnoliids and eudicots (see “Methods” section) (**Figure 2A**). In contrast, the monocots showed a weak correlation with the other two clades, especially *S. bicolor* and *O. sativa*, which belong to the Poaceae (**Figure 2A**). These data signified a closer relationship between Magnoliids and eudicots.

Next, we examined the functional implications of the shared or group-specific microsyntenic clusters among the three clades by removing the species-specific cluster (see “Methods” section). We discovered 2,839, 1,758, and 1,208 clusters specific to Magnoliids, eudicots, and monocots, respectively (**Figure 2B**). The number of synteny clusters common to Magnoliids-eudicots was still the largest (358), followed by Magnoliids-monocots (54), and eudicots-monocots (37) (**Figures 2B–E**, **S9**). As revealed in the UpSet plot, the Poaceae species *Sorghum bicolor* and *Oryza sativa* shared the largest number of clusters (6,283), followed by *Piper nigrum* and *Musa acuminata* with 3,243 and 1,651 species-specific clusters, respectively (**Figure S8C**). Four Lauraceae species also shared many clusters (1,460) (**Figure S8C**). Excluding these species-specific and clade-specific clusters, the six Magnoliids and six eudicot species shared the most clusters (39) (**Figure S8C**). These results further supported that Magnoliids and eudicots may be most closely related.

Kyoto Encyclopedia of Genes and Genomes (KEGG) and Gene Ontology (GO) enrichment analyses of eudicot-specific microsyntenic clusters showed that they were mainly associated with terms related to a series of signaling pathways (**Figure S10**). Clusters specific to the Magnoliids were mainly enriched in terms such as isoquinoline alkaloid biosynthesis, ribosome biogenesis, brassinosteroid biosynthetic process, phospholipid biosynthetic process, and secondary metabolite biosynthesis (**Figure S10**). The microsyntenic clusters in monocots were mainly enriched in terms such as histidine metabolism, chloroalkane limonene and pinene degradation, cell plate assembly, and pyrimidine metabolism (**Figure S10**). Remarkably, the synteny clusters shared by Magnoliids, eudicots, and monocots were significantly enriched in sesquiterpenoid, diterpenoid, and triterpenoid biosynthesis (**Figure S10**). This finding indicates that the genes involved in terpenoid biosynthesis are conserved among plant clades, indicating the importance of terpenoids in various plants. In addition, the unique clusters in Lauraceae were mainly enriched in isoquinoline alkaloid biosynthesis, phenylpropanoid metabolic process, secondary metabolic process and lignin metabolic process, revealing potential links to the super WDR of Lauraceae trees (**Figure S11**).

Inference of whole-genome duplication in Lauraceae species are available in **Note S7** and **Figures S12**-**S14**.

### Tandem duplicate/proximal duplicate gene duplications in Lauraceae

**Figure 3:**
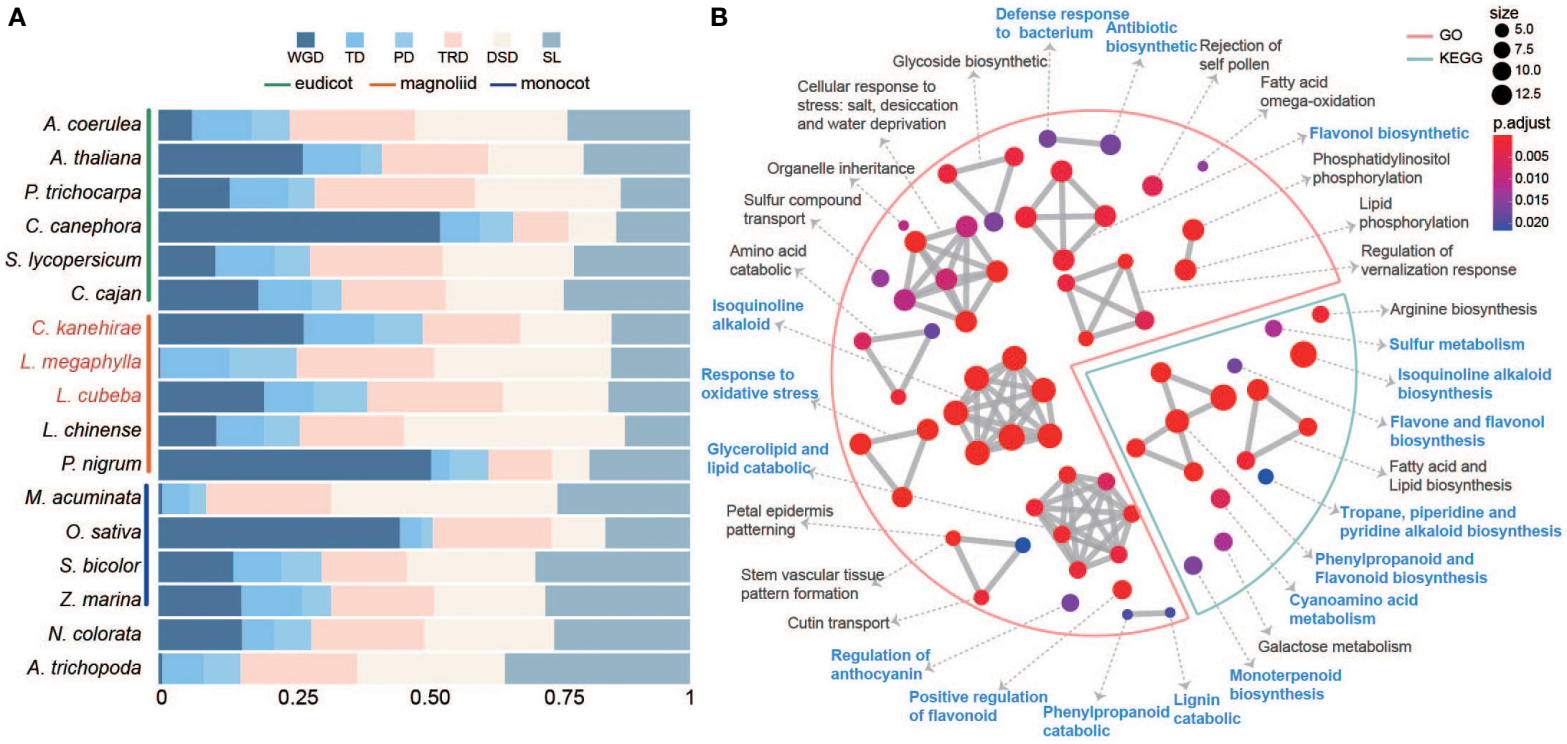
The expansion of duplicated genes. **(A)** The stacked bar chart shows the proportion of genes derived from five duplication types (WGD whole-genome duplication, TD tandem duplication, PD proximal duplication, TRD transposed duplication and DSD dispersed duplication). **(B)** GO and KEGG functional enrichment analysis of expanded genes arising from tandem and proximal duplicates (TD/PD) in Lauraceae. The red line represents GO enrichment and the blue line represents KEGG enrichment. Blue letters indicate terms related to wood decay resistance.

A total of 28,838, 22,618 and 25,951 duplicated genes originating from whole-genome duplicates (WGD), tandem duplicates (TD), proximal duplicates (PD), dispersed duplicates (DSD), and transposed duplicates (TRD) were annotated in *L. megaphylla*, *C. kanehirae*, and *L. cubeba*, respectively (**Figure 3A** and **Table S11**). Aside from 18.39% TD/PD genes in *Aquilegia coerulea*, high TD/PD ratios were found in the Lauraceae species *L. megaphylla* (19.97%), *L. cubeba* (18.36%), *C. kanehirae* (22.45%), and *P. bournei* (18.44%) (**Figure 3A** and **Table S12**). Gene families expanded *via* TD/PD duplications in Lauraceae were functionally enriched in GO categories significantly associated with wood decay resistance, such as lignin catabolism, isoquinoline alkaloid biosynthesis, flavonol biosynthesis, and phenylpropanoid catabolism (**Figure 3B**). KEGG enrichment confirmed this pattern, showing that TD/PD duplications were enriched in isoquinoline alkaloid biosynthesis, flavone and flavonol biosynthesis, phenylpropanoid and flavonoid biosynthesis, monoterpenoid biosynthesis, antibiotic biosynthesis, defense response to bacterium, response to oxidative stress, cyanoamino acid metabolism, tropane, piperidine and pyridine alkaloid biosynthesis, and sulfur metabolism (**Figure 3B**). In addition to these functions, KEGG and GO analyses also revealed significant enrichment of TD/PD duplications in the biosynthesis of various terpenoids in *L. megaphylla*, including diterpenoid, monoterpenoid, sesquiterpenoid, and triterpenoid biosynthesis (**Figure S15**) In summary, these results indicate that local gene duplication in Lauraceae contributed to the expansion of secondary metabolite biosynthesis genes related to WDR.

### Genes involved in benzylisoquinoline alkaloid biosynthesis

**Figure 4:**
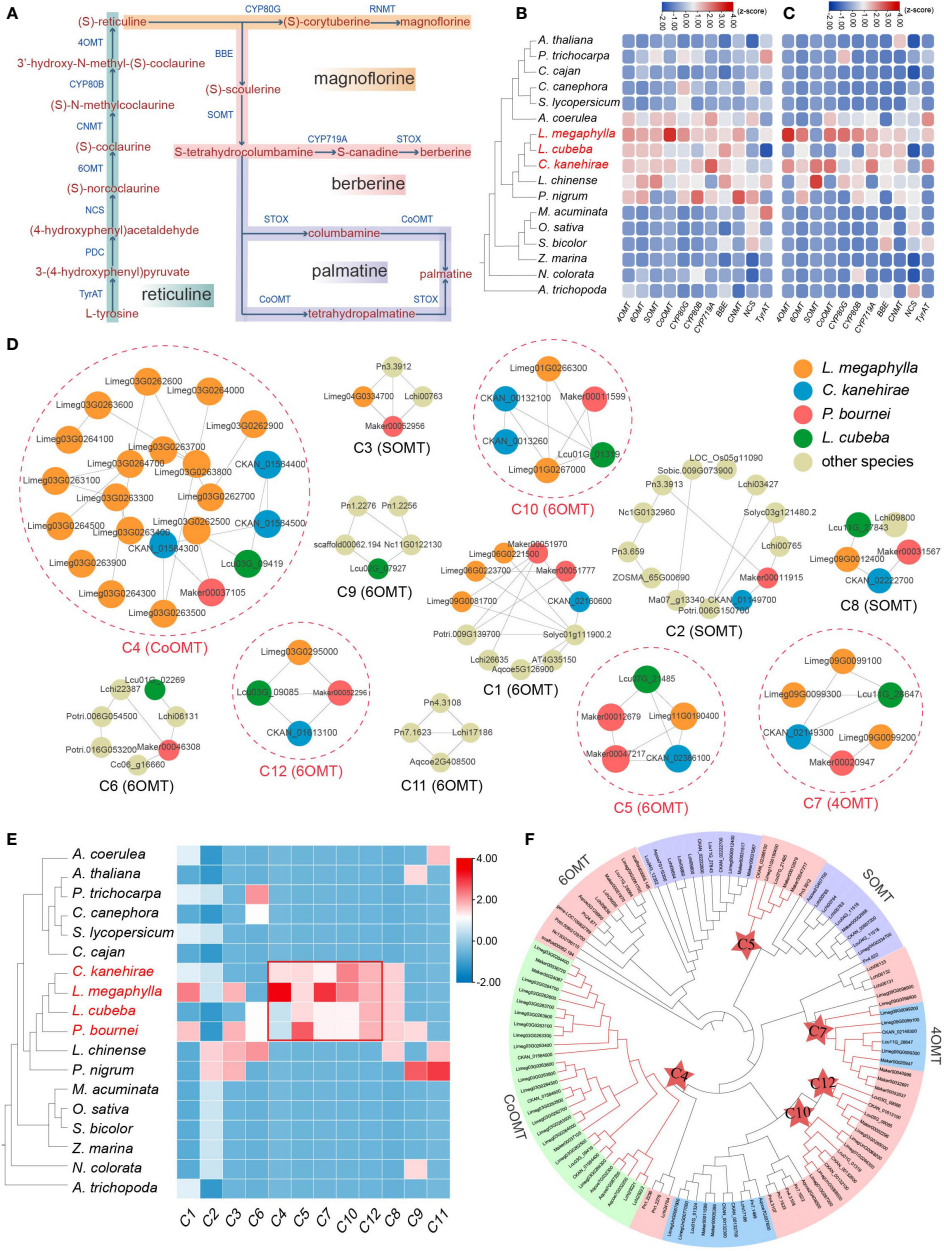
Characteristics of benzylisoquinoline alkaloid genes in Lauraceae. **(A)** The biosynthesis pathway of isoquinoline alkaloids. TyrAT, tyrosine aminotransferase; PDC, 4-hydroxyphenylpyruvate decarboxylase; NCS, (*S*)-norcoclaurine synthase; 6OMT, (RS)-norcoclaurine 6-O-methyltransferase; CNMT, (S)-coclaurine-N-methyltransferase; CYP80B, *N*-methylcoclaurine 3′-hydroxylase; 4OMT, 3′-hydroxy-N-methyl-(S)-coclaurine 4′-O-methyltransferase; CYP80G, (S)-corytuberine synthase; RNMT, reticuline N-methyltransferase; BBE, berberine bridge enzyme; SMT, (S)-scoulerine 9-O-methyltransferase; CYP719A, (S)-canadine synthase; THBO, tetrahydroberberine oxidase; CoOMT, columbamine O-methyltransferase. **(B)** Number of annotated genes in each enzyme gene family (*4OMT*, *6OMT*, *SOMT*, *CoOMT*, *CYP80G*, *CYP80B*, *CYP719A*, *BBE*, *NMT*, *NCS*, and *TyrAT*) for each species. **(C)** Proportion of tandem (TD) and proximal (PD) duplication genes in each enzyme gene family (*4OMT*, *6OMT*, *SOMT*, *CoOMT*, *CYP80G*, *CYP80B*, *CYP719A*, *BBE*, *NMT*, *NCS*, and *TyrAT*) for each species. **(D)** Microsyntenic gene clusters associated with subfamilies of the *OMT* gene family (here, *4OMT*, *6OMT*, and *CoOMT*). Circles in dashed red line denote the syntenic clusters (here, C4, C5, C7, C10 and C12) unique to Lauraceae species. **(E)** Heatmap of 12 microsyntenic clusters in **(D)**, five of which are Lauraceae-specific and highlighted by a red square. The color in the heatmap represents the gene number in each cluster for each species. **(F)** Phylogenetic analysis of *OMT* gene families. The red stars represent genes within Lauraceae-specific gene clusters identified in **(D)**.

Three different benzylisoquinoline alkaloid (BIA) biosynthesis pathways were annotated in four Lauraceae species (*L. megaphylla*, *L. cubeba*, *C. kanehirae*, and *P. bournei*), including magnoflorine, berberine, and palmatine biosynthesis pathways (**Figure 4A** and **Table S13**), all of which were important for improving decay resistance of wood. The termite antifeeding activities of berberine and palmatine have been well demonstrated (Kawaguchi et al., 1989; Park et al., 2000). A total of twelve gene families related to BIA biosynthesis were identified. The enzymes 4OMT, 6OMT, SOMT, and CoOMT belong to the O-methyltransferase (OMT) family, and CYP80G, CYP80B, and CYP719A belong to the cytochrome P450 (CYP) family. These enzymes are mainly found in Magnoliids and *A. coerulea*, but rarely in monocots and other core eudicots (**Figure 4B**). In addition, TD/PD duplication contributed the most BIA biosynthesis genes in Lauraceae, especially the *4OMT*, *6OMT*, *CoOMT*, *CYP80G*, *CYP80B*, *CYP719A*, and *CNMT* ((S)-coclaurine-N-methyltransferase) genes (**Figures 4C**, **S16**, and **Table S14**).

A total of 12 microsyntenic clusters were identified as related to *OMT* gene families (here, *4OMT*, *6OMT*, and *CoOMT*) (**Figure 4D**). Five of these 12 microsyntenic clusters were specific to Lauraceae, including C5, C10 and C12 associated with *6OMT*, C7 associated with *4OMT*, and C4 with *CoOMT* (**Figures 4D–F**). These genes on Lauraceae-specific microsyntenic clusters may play an important role in the unique WDR of Lauraceae species.

**Figure 5:**
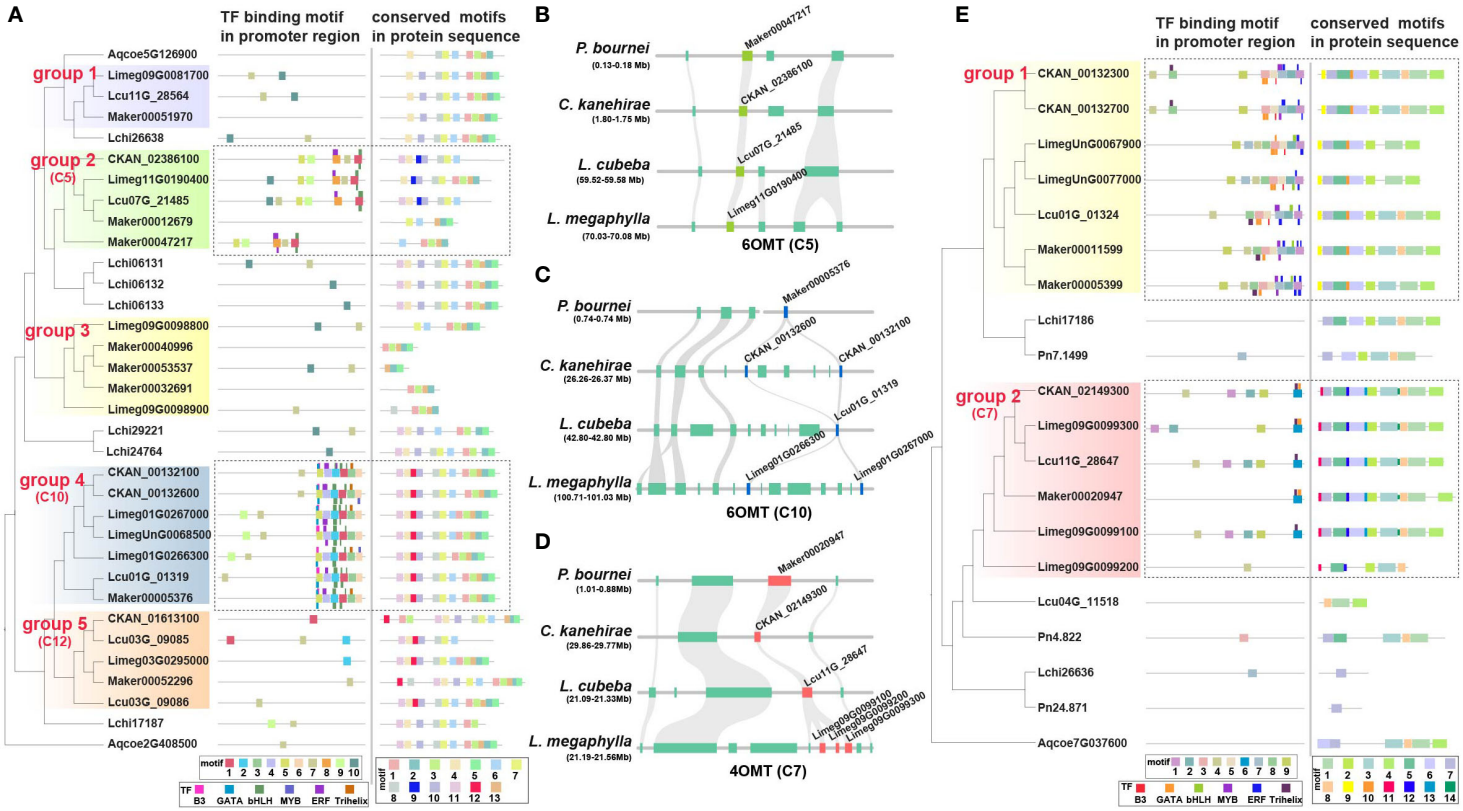
Specificity of *6OMT* and *4OMT* genes in Lauraceae. **(A)** The different panels illustrate the phylogenetic tree of the *6OMT* gene family (left), the distribution of motifs in the promoter sequences and the predicted transcription factor binding sites (TFBS) (middle), and the distribution of motifs in protein sequences (right). Thick squares represent motifs and thin ones represent TFBSs. Dashed boxes highlight genes and promoter motifs unique to Lauraceae species. **(B)** The syntenic block containing the *6OMT* gene family within the Lauraceae-specific microsynteny gene cluster (C5), which was identified in **Figures 4D, E**. This syntenic block was compared among *P. bournei*, *C. kanehirae*, *L. cubeba*, and *L. megaphylla*. Chartreuse squares represent the *6OMT* genes and aquamarine squares represent other genes on the syntenic block. **(C)** The syntenic block containing the *6OMT* gene family within the Lauraceae-specific microsynteny gene cluster (C10), which was identified in **Figures 4D, E**. This syntenic block was compared among *P. bournei*, *C. kanehirae*, *L. cubeba*, and *L. megaphylla*. Blue squares represent *6OMT* genes and aquamarine squares represent other genes on the syntenic block. **(D)** The syntenic block containing the *4OMT* gene family within the Lauraceae-specific microsynteny gene cluster (C7), which was identified in **Figures 4D**, **E**. This syntenic block was compared among *P. bournei*, *C. kanehirae*, *L. cubeba*, and *L. megaphylla*. Red squares represent *4OMT* genes and aquamarine squares represent other genes on the syntenic block. **(E)** The different panels show the phylogenetic tree of the *4OMT* gene family (left),the distribution of motifs in the promoter sequences and the predicted transcription factor binding sites (TFBS) (middle), and the distribution of motifs in protein sequences (right). Thick squares represent motifs and thin ones represent TFBSs. Dashed boxes highlight genes and promoter motifs unique to Lauraceae species.

6OMT is involved in the rate-limiting step of isoquinoline biosynthesis (Robin et al., 2016). Phylogenetic analysis showed that the *6OMT* genes in Lauraceae could be divided into five groups (**Figure 5A**). Genes of the Lauraceae-specific clusters C5, C10 and C12 were located in groups 2,4 and 5 respectively (**Figures 5A–C**). Protein sequence analysis found a Lauraceae-specific motif (motif 9) among genes in group 2 (C5). Genes in group 4 (C10) and 5 (C12) shared another Lauraceae-specific motif (motif 12) (**Figure 5A**). In addition, we identified several conserved motifs unique to Lauraceae through sequence analysis of gene promoters. Motif 1 existed in both C5 and C10 genes and overlapped with the predicted binding sites of bHLH transcription factors (TFs) (**Figure 5A**). Motif 8 was specific to C5 genes and overlapped with the predicted binding sites of ERF TFs (**Figure 5A**). Interestingly, six motifs (motif 5, 4, 2, 1, 3, and 6) formed a tandem cluster unique to Lauraceae genes in group 4 (C10). These motifs were the predicted binding sites of GATA, B3, ERF, bHLH, Trihelix, and MYB transcription factors (**Figure 5A**).

In addition to 6OMT, 4OMT is also an important rate-limiting enzyme in BIA biosynthesis (Inui et al., 2012). The *4OMT* genes in Lauraceae were divided into two groups, with C7 genes located in group 2 (**Figures 5D, E**). Four Lauraceae-specific and conserved motifs (motifs 10-14) were identified among the protein sequences of these group 2 (C7) genes (**Figure 5E**). Although no Lauraceae-specific microsyntenic cluster in group 1, phylogenetic analysis results showed that they were located in Lauraceae-specific clades, and two Lauraceae-specific motifs (motif 9 and motif 10) were identified (**Figure 5E**). Sequence analysis of gene promoters revealed that both groups (group1 and group 2) shared a common Lauraceae-specific DNA motif (motif 1), but only group 1 contained potential MYB transcription factor binding sites (TFBSs) (**Figure 5D**). One Lauraceae-specific promoter motif (motif 6) in group 2 (C7) overlapped with predicted WRKY and bHLH TFBSs (**Figure 5E**). Interestingly, we identified a unique promoter motif (motif 8) among group 1 genes, and found a conserved cluster formed by six motifs (motif 8, 9, 3, 5, 2, and 1) that overlap with WRKY, bHLH, B3, ERF, MYB, and C2H2 TFBSs. These TFBS clusters may play key roles in coordinating specific gene expression as well as efficient activation and regulation of alkaloid biosynthesis.

Columbamine O-methyltransferase (CoOMT) is a vital enzyme that catalyzes the formation of tetrahydropalmatine, an isoquinoline alkaloid. The C4, a Lauraceae-specific microsyntenic cluster contained all *CoOMT* genes (**Figures 4E**, **S17A**). We found that TD/PD duplications occurred before Lauraceae speciation, producing three major *CoOMT* groups (group 1, 2, and 3) (**Figures S17A, 17B**). In *L. megaphylla*, all members of the *CoOMT* family were found in one TD/PD cluster on chromosome 3 (**Figure S17B**). Two Lauraceae-specific motifs (motif 9 and motif 10) among *CoOMT* protein sequences were identified (**Figure S17A**). In addition, we identified two Lauraceae-specific promoter motifs (motif 3 and motif 4), of which motif 3 is the potential TFBS of WRKY, ERF, and MYB TFs, and motif 4 is the potential TFBS of bHLH TFs (**Figures S17A, 17C**).

Cytochrome P450 monooxygenases (CYPs) play an important role in the structural and functional diversity of alkaloids. The *CYP80B*, *CYP80G*, and *CYP719A* gene families play key oxidative roles in BIA metabolism (Hagel and Facchini, 2013; Nguyen and Dang, 2021). A total of 20 microsyntenic clusters were identified as related to the *CYP* gene family, among which three clusters were unique to Lauraceae (C11, C12, and C5) (**Figures S18A, 18B**, **S19**). Specifically, microsyntenic cluster C11 is related to the *CYP719A* family, and C12 and C5 are related to the *CYP80G* family. TD/PD expansion of genes on C5 cluster occurred in all Lauraceae species, especially in *L. megaphylla* (**Figure S19A**).

CYP719A catalyzes the conversion of (S)-tetrahydrocolumbamine to (S)-tetrahydroberberine, and is an essential enzyme in berberine biosynthesis (Ikezawa et al., 2003). According to the phylogenetic tree, *CYP719A* genes from Lauraceae can be divided into two groups. All members of C11 were classified into group 2, and these genes are located in a species-specific TD/PD cluster found on *L. megaphylla* chromosome 8 (**Figure S20B**). A motif unique to Lauraceae (motif 12) was discovered in the protein sequences of these *CYP719A* genes (**Figures S20A, 20C**). Further, three Lauraceae-specific motifs (motif 1, motif 4, and motif 8) were found in the promoters (**Figures S20A, 20B**). Among these motifs, motif 1 contains NAC TFBSs, motif 4 contains bHLH and ERF TFBSs, and motif 8 contains MYB TFBSs (**Figure S20C**). Notably, TFs such as bHLH, NAC, WRKY, and MYB have been implicated in the regulation of BIA biosynthesis in plants (Yamada et al., 2011; Zhou and Memelink, 2016; Deng et al., 2018). Here, we identified Lauraceae-specific and conserved protein sequences, TFBS motifs, and TFBS clusters among BIA biosynthesis genes. It is found that the genes related to BIA biosynthesis in Lauraceae species are significantly different from those in other species. These findings are valuable in the genetic dissection of BIA biosynthesis in Lauraceae species.

### Characterization of genes involved in phenolic compound biosynthesis

**Figure 6:**
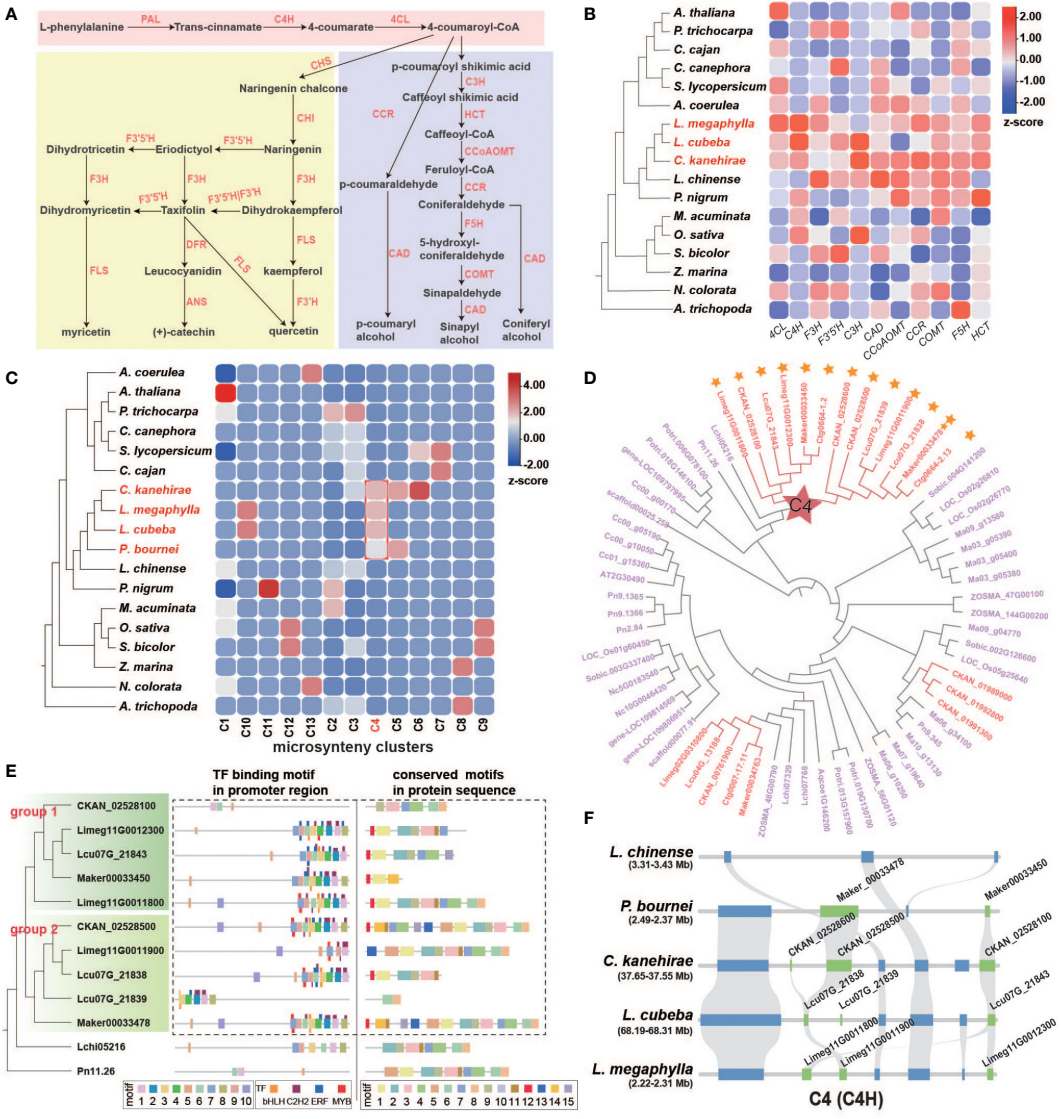
Characteristics of flavonoid and lignin genes in Lauraceae. **(A)** Biosynthesis pathways of general phenylpropanoids, flavonoids, and lignin. PAL, phenylalanine ammonia-lyase; C4H, cinnamate-4-hydroxylase; 4CL, 4-coumarate CoA ligase 4; CHS, chalcone synthase; CHI, chalcone isomerase; F3H, flavanone 3-hydroxylase; FLS, flavonol synthase; F3′H, flavonoid 3′-hydroxylase; F3′5′H, flavonoid 3′,5′-hydroxylase; DFR, dihydroflavonol 4-reductase; ANS, anthocyanidin synthase; C3′H, p-coumaroyl shikimate 3′-hydroxylase; CCR, cinnamoyl-CoA reductase; CAD, (hydroxy)cinnamyl alcohol dehydrogenase; HCT, hydroxycinnamoyl-CoA:shikimate/quinate hydroxycinnamoyltransferase; CCoAOMT, caffeoyl-CoAO methyltransferase; F5H, coniferaldehyde/ferulate 5-hydroxylase; COMT, caffeicacid/5-hydroxyferulic acid O-methyltransferase. **(B)** Proportion of tandem and proximal duplication genes in *4CL*, *C4H*, *F3H*, *F3′5′H*, *C3′H*, *CAD*, *CCoAOMT*, *COMT*, *F5H* and *HCT* gene families in each species. **(C)** Heatmap of 13 microsyntenic clusters of *C4H* gene families in 18 species. The Lauraceae-specific cluster is highlighted by a red square. Colors in the heatmap indicate gene number in each cluster for each species. **(D)** Phylogenetic analysis of *C4H* gene families. The gene names of Lauraceae species are shown in red, and red stars represent Lauraceae-specific gene clusters identified in **(C)**. Yellow stars show the tandem and proximal duplication (TD/PD) genes. **(E)** The phylogenetic tree of the C4H gene family (left), the distribution of motifs in the promoter sequences and the predicted transcription factor binding sites (TFBS) (middle), and the distribution of motifs in protein sequences (right) are shown. Thick squares represent motifs and thin ones represent TFBSs. Dashed boxes highlight genes and promoter motifs unique to Lauraceae species. **(F)** The syntenic block containing the *C4H* gene family within the Lauraceae-specific microsynteny gene cluster (C4) identified in **(C)**. Here, this syntenic block was compared among *L. chinense*, *P. bournei*, *C. kanehirae*, *L. cubeba*, and *L. megaphylla*. Chartreuse squares represent *C4H* genes and blue squares represent other genes on the syntenic block.

We next examined the lignin and flavonoid biosynthesis pathways, which are the downstream branches of phenylpropanoid metabolism related to phenol biosynthesis (**Figure 6A** and **Table S15**). Phenolic compounds can protect wood from decaying organisms and improve WDR. The key reactions of general phenylpropanoid biosynthesis involve three enzymes: phenylalanine ammonia-lyase (PAL), cinnamate 4-hydroxylase (C4H), and 4-coumarate coenzyme A ligase (4CL). Among these enzymes, we found that the *C4H* and *4CL* genes underwent remarkable TD/PD duplication events in Lauraceae (**Figures 6A, B**, **S21**, and **Table S16**).

Our microsynteny analysis of *C4H* genes revealed a Lauraceae-specific cluster (C4) (**Figure 6C**). Genes of this C4 cluster were divided into two groups resulted from the Lauraceae-specific TD/PD duplication (**Figure 6D**). Two Lauraceae-specific protein motifs (motif 12 and motif 13) were identified in these C4 cluster genes (**Figures 6E, F**, **S22**). We also found two motifs (motif 2 and motif 4) specific to Lauraceae in the promoter regions of these genes (**Figures 6E**, **S22**). Motif 2 overlapped with C2H2 TFBSs and motif 8 with that of MYB and ERF TFs (**Figures 6E**, **S22**). Members of all these TF families are involved in the regulation of phenylpropanoid biosynthesis (Ma et al., 2017; Mondal and Roy, 2018; Teng et al., 2018). In addition, among the C4 genes, these two Lauraceae-specific motifs were clustered together with motifs 7, 9, 6, 3, 1, and 8, forming a very distinct cluster of ERF, MYB, bHLH, and ERF TFBSs. This motif cluster was shared among Lauraceae species and *L. chinense* (**Figures 6E**, **S22**).

**Figure 7:**
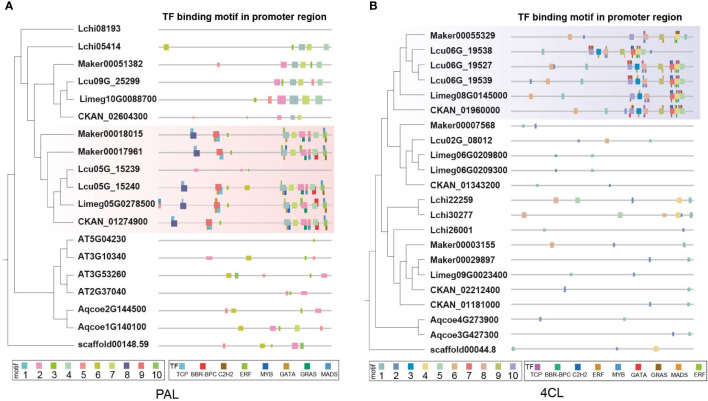
Characterization of *PAL* and *4CL* genes in Lauraceae species. **(A)** Different panels represent of the phylogenetic tree of the *PAL* gene family (the left), the distribution of motifs in the promoter sequence and the predicated transcription factor binding sites (TFBS) (the right). Fat squares represent the motifs and thin ones the TFBSs. Red boxes highlight the promoter motifs uniquely found among the Lauraceae species. **(B)** Different panels represent of the phylogenetic tree of the *4CL* gene family (the left), the distribution of motifs in the promoter sequence and the predicated transcription factor binding sites (TFBS) (the right). Fat squares represent the motifs and thin ones the TFBSs. Purple boxes highlight the promoter motifs uniquely found among the Lauraceae species.

Sequence analysis of promoter regions revealed two motifs (motif 8 and motif 9) unique to Lauraceae of *PAL* genes (**Figure 7A**). Motif 8 overlapped with TCP TFBSs and motif 9 with that of TCP and GATA TFs (**Figure 7A**). TCP TFs play an important role in plant defense and have been found to enhance flavonoid biosynthesis of *Arabidopsis thaliana* (Li and Zachgo, 2013; Li, 2014). Moreover, overexpression of a GATA gene can enhance the activity of the phenylpropanoid biosynthesis pathway in *Solanum lycopersicum* (Zhao et al., 2021b). Similar to the *PAL* genes, although there was no Lauraceae-specific collinearity cluster found related to *4CL* genes, a motif in the promoters unique to Lauraceae (motif 7) was identified and overlapped with C2H2 TFBSs (**Figure 7B**). Moreover, conserved TFBS clusters were also found among the promoters of *PAL* and *4CL* genes. These TFBSs were of TFs belonging to the TCP, BFR-BPC, C2H2, ERF, MYB, GATA, and GRAS families (**Figures 7A, B**).

**Figure 8:**
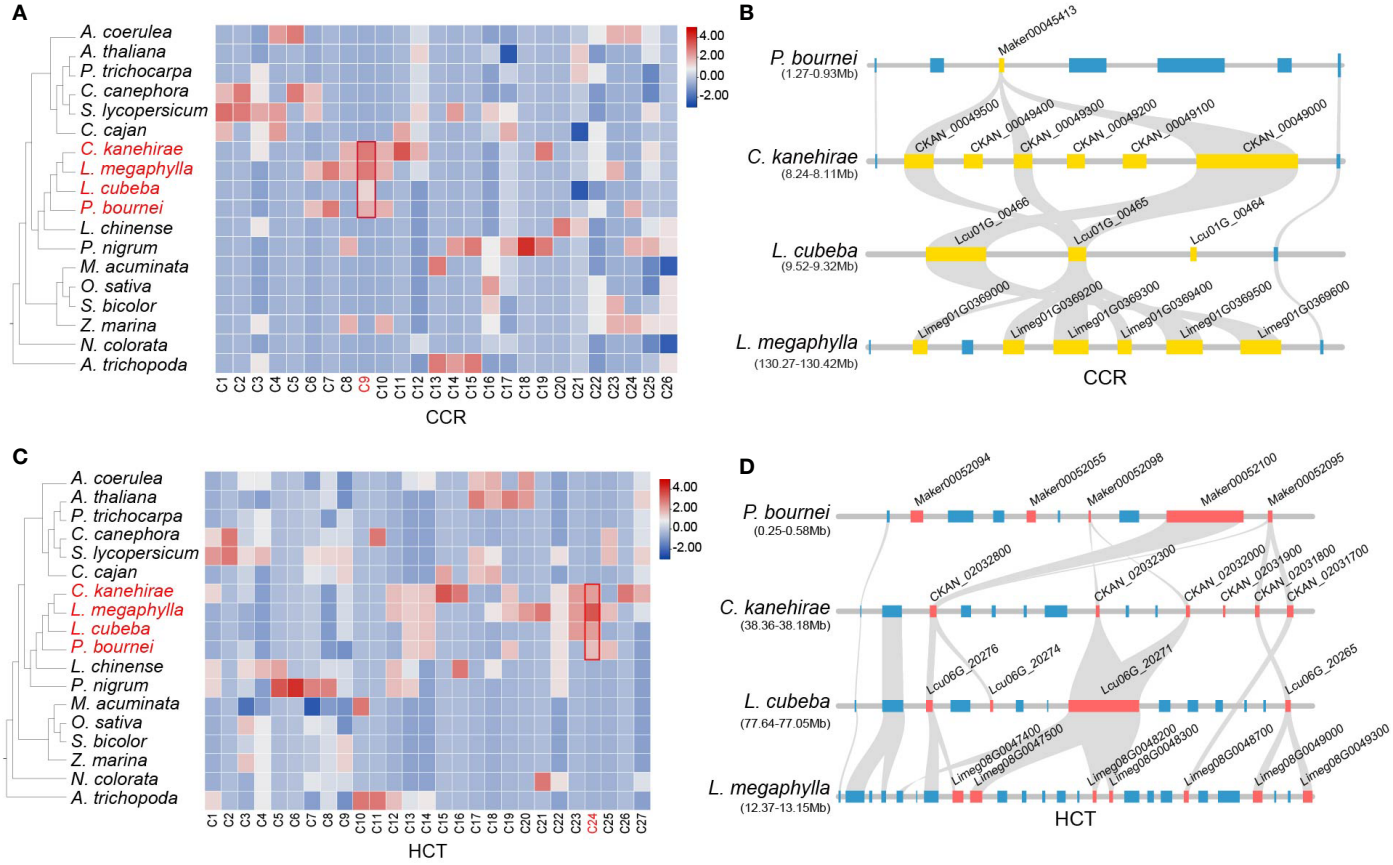
Lauraceae-specific *CCR* and *HCT* genes in lignin biosynthesis pathway. **(A)** Heatmap of 26 microsynteny clusters identified to be related with *CCR* gene family, one of which specific to Lauraceae were highlighted in a red square. Color in the heatmap was determined by the gene number found in each cluster for each species. **(B)** The syntenic block containing of *CCR* gene family within the Lauraceae-specific microsynteny gene cluster (C9) identified in **(A)**. Here this syntenic block was compared among *P. bournei*, *C. kanehirae*, *L. cubeba* and *L. megaphylla*. Yellow squares represent the *CCR* genes and blue ones represent other genes on the syntenic block. **(C)** Heatmap of 27 microsynteny clusters identified to be related with *HCT* gene familiy, one of which specific to Lauraceae were highlighted in a red square. Color in the heatmap was determined by the gene number found in each cluster for each species. **(D)** The syntenic block containing of *HCT* gene family inside the Lauraceae-specific microsynteny gene cluster (C24) in **(C)**. Here this syntenic block was compared among *P. bournei*, *C. kanehirae*, *L. cubeba* and *L. megaphylla*. Red squares represents the *HCT* genes and blue ones represents other genes on the syntenic block.

The biosynthesis pathways of taxifolin, myricetin, catechin, quercetin, and kaempferol have been annotated in Lauraceae species (**Figure 6A**). All of these flavonoids have been reported to improve plant WDR (Nascimento et al., 2013). TD/PD duplications accounted for expansions of *F3H* (flavanone 3-hydroxylase) and *F3′5′H* (flavonoid 3′,5′-hydroxylase) genes in Lauraceae (**Figures 6B**, **S21**, and **Table S17**). F3H is an important rate-limiting enzyme in flavonoid biosynthesis pathway. Enzymatic gene families of the lignin biosynthesis pathway include *C3′H*, *HCT*, *CCR*, *CAD*, *CCoAOMT*, *F5H*, and *COMT*, all of which were expanded through TD/PD duplications in Lauraceae (**Figures 6B**, **S23**, and **Table S18**). Microsynteny analysis revealed two Lauraceae-specific conserved gene clusters (C9 and C24) associated with lignin pathway genes (*HCT* and *CCR*) (**Figures 8A–D**). Although no Lauraceae-specific motifs and TFBSs were found among genes of the C9 and C24 clusters, all of these genes showed obvious TD/PD expansion (**Figures 8B, D**).

Remarkable TD/PD duplications were also found for *TPS* gene family of Lauraceae species, which may be associated with the super WDR. Details are available in **Note S8**, **Figures S24**-**26** and **Table S19**.

## Discussion

Our genomic investigation, especially the gene microsynteny profiling, may contribute to resolving the phylogenetic position of Magnoliids relative to eudicots and monocots, the other two major angiosperm groups. Although multiple assemblies of magnoliid genomes have been published, such as *C. kanehirae* (Chaw et al., 2019a), *L. chinense* (Chen et al., 2019), *P. nigrum* (Hu et al., 2019), *P. americana* (Rendon-Anaya et al., 2019), *P. bournei* (Chen et al., 2020a), *L. cubeba* (Chen et al., 2020b), *C. salicifolius* (Lv et al., 2020), and *C. praecox* (Shang et al., 2020a), the phylogenetic placement of Magnoliids still remains unclear. Our phylogenetic analyses using three different methods (concatenation-, coalescent-, and microsynteny-based approaches) confirmed that Magnoliids are the sister group of eudicots, which is in line with previous genomic analyses (Chaw et al., 2019a; Lv et al., 2020; Shang et al., 2020a) and phylotranscriptomic analyses of 92 streptophytes (Wickett et al., 2014) and 20 representative angiosperms (Zeng et al., 2014). In addition, the microsyntenic clusters of 16 species in Magnoliids, eudicots, and monocots were further analyzed. There were significantly more shared clusters in Magnoliids-eudicots compared with Magnoliids-monocots and eudicots-monocots, which strongly supports the finding that Magnoliids and eudicots are sister groups. The three clades were enriched in different GO and KEGG terms, indicating their functional divergence. The genes of Lauraceae-specific microsyntenic clusters were significantly enriched in terms including isoquinoline alkaloid biosynthesis, phenylpropanoid metabolic and lignin metabolic processes, suggesting that various Lauraceae-specific biochemical processes may influence its wood decay resistance.

A variety of bioactive compounds, including terpenoids, alkaloids, and phenolic compounds such as flavonoids, have been associated with WDR (Nascimento et al., 2013; Anouhe et al., 2018). In addition to the dual fungicidal and antioxidant effects of bioactive compounds, other factors such as lignin content also impact WDR (Vance et al., 1980; Nascimento et al., 2013; Mounguengui et al., 2016). Apart from annotating enzymes involved in the biosynthesis of isoquinoline alkaloids, flavonoids, terpenoids, and lignins, we characterized genes, gene syntenies, gene expansions, and gene promoter motifs specific to Lauraceae, which help to track genomic characters potentially related with the super wood decay resistance.

The biosynthetic pathways of three benzylisoquinoline alkaloids (BIA), namely magnoflorine, berberine, and palmatine, were annotated in Lauraceae species. Both berberine and palmatine exhibit significant antifeedant activity against termites (Kawaguchi et al., 1989). Magnoflorine is an aporphine-type BIA that has antibacterial and insecticidal effects, and may also play a role in improving WDR (Okon et al., 2020). Our comparative analyses demonstrated that the *OMT*, *CYP*, and *BBE* gene families involved BIA biosynthesis showed specific expansion in Lauraceae. Most members of these gene families originated from TD/PD duplications, which greatly enriched the enzymatic genes of the BIA biosynthesis pathway. These data indicate the significant value of TD/PD duplications in BIA biosynthesis. In the *OMT* gene family, a total of four Lauraceae-specific microsyntenic clusters were identified, including genes of the *4OMT*, *6OMT*, and *CoOMT* subfamilies. Again, TD and PD duplications were associated with significant expansion of the *CoOMT* gene family in *L. megaphylla*, which may have contributed to the accumulation of palmatine, thereby further improving WDR.

In addition to the Lauraceae-specific gene microsyntenic clusters uncovered for the biosynthesis of bioactive compounds related to WDR, we also found conserved TFBS clusters in the promoter regions of genes in these conserved clusters. These conserved TFBS clusters suggest conserved transcriptional regulation of secondary metabolite biosynthesis efficiency, which may lead to the high WDR trait shared among many Lauraceae woods. In the *OMT* gene family, the Lauraceae-specific promoter motifs were mainly TFBSs of bHLH, MYB, ERF and WRKY TFs. In the *CYP* gene family, the conserved promoter motifs were generally TFBSs of bHLH, MYB, ERF, and NAC TFs, all of which have been reported to be involved in the regulation of BIA biosynthesis (Yamada et al., 2011; Deng et al., 2018). In addition, we found that B3, GATA, Trihelix, and C2H2 TFs may bind these Lauraceae-specific TFBS clusters. However, their involvement in the regulation of alkaloid biosynthesis requires further evaluation. Compared with other species, the unique characteristics of Lauraceae species in BIA biosynthesis suggest that isoquinoline alkaloids may play a large proportion of roles in the decay resistance of Lauraceae.

There are diverse metabolic branches downstream of the general phenylpropanoid biosynthesis. Of these branches, we investigated the lignin and flavonoid pathways in the present study. The *C4H* and *4CL* genes of the general phenylpropanoid pathway, *F3H* and *F3′5′H* of the flavonoid pathway, and all gene families of the lignin pathway have undergone significant TD/PD duplication in Lauraceae. C4H is the second key enzyme in the general phenylpropanoid biosynthesis pathway, and belongs to the CYP73A subfamily. C4H directly affects the biosynthesis and yield of flavonoids and lignin in plants (Ryan et al., 2002; Millar et al., 2007). Lauraceae-specific genes were found in the *C4H* gene family. In addition to carrying motifs in the coding and promoter regions that were different from other species, these *C4H* genes also had unique TFBS clusters specific to Lauraceae. Such TFBSs in the clusters are adjacent to each other, including binding sites of bHLH, C2H2, ERF, and MYB TFs, which all have important regulatory functions in phenylpropanoid biosynthesis (Ma and Constabel, 2019; Yadav et al., 2020; Meng et al., 2021). Moreover, TD/PD events also occurred in the Lauraceae-specific genes of the *C4H* gene family, which greatly increased their coding space, and further contributed to the WDR of Lauraceae species. In addition, Lauraceae-specific TFBS clusters were also found in the promoter regions of genes encoding PAL and 4CL. PAL is a rate-limiting enzyme that catalyzes the first step in the phyenylpropanoid biosynthesis pathway. Thus, it plays an important role in phenylpropanoid biosynthesis (Zhao et al., 2021a). 4CL, the third enzyme in the general phenylpropanoid biosynthesis pathway, participates in monolignol biosynthesis through the production of p-coumaroyl-CoA, a precursor for the biosynthesis of lignin, flavonoid compounds, and plant defense compounds (isoflavonoids). Therefore, compared with other plant groups, general phenylpropanoid biosynthesis genes in Lauraceae are highly unique, which affects the biosynthesis of flavonoids and lignin and may improve the natural durability of Lauraceae wood. Studies found that functional disruption of *CCR* and *HCT* genes affects lignin content (Thévenin et al., 2011; Wang et al., 2015). Although microsyntenic clusters were notable in the *CCR* and *HCT* gene families, no unique motifs were found among the protein sequences and promoter regions of homologous genes. We suspected that these Lauraceae-specific genes may have arisen more recently and have not yet diverged significantly from the original genes, in addition, these genes also showed significant TD/PD expansion.

In summary, we investigated the WDR of Lauraceae species by identifying microsynteny clusters among different angiosperm lineages. The Lauraceae-specific biosynthetic genes related to WDR, the conserved motifs of the encoding proteins, the unique and conserved gene expansion and TFBS clusters may play a vital role in increasing and regulating WDR, which may be the main reason for the super decay resistance of Lauraceae. The present genome resources and investigation lay the foundation for molecular breeding or genetic engineering of Lauraceae, and provide key resources for further exploration of the naturally durable wood of Lauraceae species.

## Materials and methods

### Plant material

A healthy, fruitful, mature *L. megaphylla* individual was selected and used for whole genome sequencing. This individual was collected from naturally regenerated forest at the National Tree Breeding Station for Nanmu in Zhuxi, Forest Farm of Zhuxi County, Hubei, China. For RNA sequencing, flower buds, stems, buds, and leaves were sampled from healthy trees in the same location, with three replicates per tissue. Tissues were immediately flash frozen and stored at -80 °C for subsequent nucleic acid extractions.

### Genome sequencing

For Nanopore sequencing, PromethION libraries were prepared and sequenced on a Nanopore PromethION platform. For Illumina sequencing, 150-bp paired-end (PE) libraries were prepared for sequencing on an Illumina HiSeq X Ten platform. The Hi-C library prepared with the MboI restriction enzyme was sequenced in an Illumina HiSeq X Ten to generate 1488.194 million reads (~223 Gb, roughly 170x coverage of the assembled genome) from 150-bp PE reads. For RNA sequencing, four tissues (flower buds, stems, buds, and leaves) were used to construct mRNA sequencing libraries, after which 150-bp PE sequencing was performed in an Illumina HiSeq X Ten. RNA sequencing produced 996.020 million raw reads (~145 Gb).

More details regarding genome sequencing are available in **Note S2**.

### *De novo* genome assembly and quality control

*De novo* genome assembly involved three steps: primary assembly, Hi-C scaffolding, and polishing. First, we used SMARTdenovo (see “URLs” section), WTDBG (version 2.1) (Ruan and Li, 2020), and Canu (version 1.7) (Koren et al., 2017) to generate four of the primary assemblies from ONT long reads. Then, one primary assembly (v0.3, with reasonably sized assembly, fewest contigs, and highest contig N50) was chosen as the optimal assembly, and further polished with three rounds of pilon (see “URLs” section) with clean Illumina reads to generate assembly v1.0. Based on Hi-C data and assembly v1.0, primary scaffolds were produced with 3D-DNA (version 180922) (see “URLs” section). These scaffolds were inspected and manually corrected using Juicebox (version 1.8) (see “URLs” section) and re-scaffolded by 3D-DNA. Afterwards, we optimized the new scaffolds with gap closing using LR_Gapcloser (version 1.1) (see “URLs” section) followed by four rounds of pilon polishing.

Benchmarking Universal Single Copy Orthologs (BUSCO) and LTR Assembly Index (LAI) were used to assess genome completeness and continuity. To evaluate the completeness of the assembly and uniformity of the sequencing, 178 Gb of ONT reads, 160 Gb of clean Illumina reads, and 90 Gb of RNA sequencing reads were aligned to the assembly genome using BWA-MEM (see “URLs” section), minimap2 (Li, 2018), and HiSat2 (version 2.1.0) (see “URLs” section), respectively.

More details of genome assembly are available in **Note S3**.

### Genome annotation

Protein-coding genes were predicted using the MAKER2 pipeline (Holt and Yandell, 2011) including *ab initio*, homolog proteins, and EST-based prediction methods. We annotated non-coding RNAs (ncRNAs) with several databases and software including tRNAscan-SE (version 1.3.1) (Lowe and Eddy, 1997), RNAMMER (version 1.2) (Lagesen et al., 2007), Rfam database (version 9.1) (see “URLs” section), and BLASTN (version 2.2.28+).

Functions of predicted genes were annotated using sequence similarity searches by BLAT (version 36) (Kent, 2002) with 30% identity and 1e-05 E-value cutoff, as well as domain similarity annotations using InterProScan (version 5.27-66.0) (see “URLs” section). The completeness of genome annotation was assessed using BUSCO. Centurion (Varoquaux et al., 2015) was used to infer the location of all centromeres in the genome based on corrected Hi-C data.

Repeated elements were annotated using RepeatModeler (version 1.0.10) (see “URLs” section) and RepeatMasker (version 4.0.7, rmblast-2.2.28) (see “URLs” section) with homology-based and *de novo* approaches. In addition, we examined classification, age distribution, birth, and death of LTR-RTs.

More details of genome annotation are available in **Note S4** and **S5**.

### Gene family and phylogenetic inference

To determine the phylogenetic relationships among Magnoliids, we used Orthofinder (version 2.3.1) (Emms and Kelly, 2019) to identify gene families from 6 eudicots including *Aquilegia coerulea* (Filiault et al., 2018), *Populus trichocarpa* (Tuskan et al., 2006)*, Arabidopsis thaliana* (Michael et al., 2018)*, Coffea canephora* (Denoeud et al., 2014)*, Solanum lycopersicum* (Sato et al., 2012) and *Cajanus cajan* (Varshney et al., 2012), 4 monocots including *Zostera marina* (Olsen et al., 2016)*, Sorghum bicolor* (Deschamps et al., 2018)*, Musa acuminata* (D’Hont et al., 2012) *and Oryza sativa* (Ouyang et al., 2006), 6 Magnoliids including *Piper nigrum* (Hu et al., 2019)*, Liriodendron chinense* (Chen et al., 2019)*, Persea americana* (Rendon-Anaya et al., 2019), *Cinnamomum kanehirae* (Chaw et al., 2019a)*, Litsea cubeba* (Chen et al., 2020b) and *Lindera megaphylla* and 2 outgroup species including *Amborella trichopoda* (Albert et al., 2013) and *Nymphaea colorata* (Zhang et al., 2020b). A total of 34,888 orthogroups, including 112 orthologous single-copy gene families and 885 low-copy orthologs with minimum of 83.3% of species having single-copy genes in any orthogroup. Amino acid sequence alignment was performed on these low-copy genes using MUSCLE (version 3.8.31) (Edgar, 2004).

Phylogenetic trees were constructed using concatenation-, coalescent-, and microsynteny-based approaches (Zhao et al., 2021c). For the concatenation-based approach, the maximum likelihood tree was constructed based on concatenated low-copy amino acid sequences with IQ-TREE (version 1.6.7) (Nguyen et al., 2014), employing the best-fit model (-m JTT+F+R5) with ultrafast bootstrapping (-bb 1000). For the coalescent-based approach, gene trees of 855 low-copy gene families were inferred by IQ-TREE. Next, we removed low bootstrap support branches (less than 50%) using the Newick utilities. Then, gene trees were used to construct species trees with ASTRAL-pro. Quartet support of each node was estimated for this coalescent tree. Finally, the microsynteny-based method included two steps. First, after an all-by-all protein alignment of the whole genome was performed using DIAMOND (Buchfink et al., 2015), pairwise synteny blocks were identified using MCScanX (Wang et al., 2012). Then, microsyntenic clusters were detected using Infomap (see “URLs” section).

The maximum likelihood (ML) phylogenetic tree was generated with IQ-TREE (version 1.6.7), using the Mk+R+FO model and ultrafast bootstrapping (-bb 1000). The ML tree constructed using the coalescent-based approach was used as an input tree to estimate divergence time with the MCMCTree program in the PAML package (version 4.9h) (Yang, 2007). Dating was calibrated according to the TimeTree web service (http://www.timetree.org/) by placing soft bounds at four split nodes as constraints for calibrating tree age: (1) the *A. trichopoda* node (173-199 Mya), (2) *L. chinense* (117-130 Mya), (3) *O. sativa*-*S. bicolor* (42-52 Mya), and (4) *P. trichocarpa*-*A. thaliana* (98-177 Mya). Expansion and contraction of gene families were inferred with CAFÉ (version 4.1) (De Bie et al., 2006).

Kyoto Encyclopedia of Genes and Genomes (KEGG) and Gene Ontology (GO) enrichment analyses were performed using the R package clusterProfiler (version 3.6.0) (Yu et al., 2012).

Additional details are available in **Note S6**.

### Analysis of microsyntenic clusters

The microsyntenic clusters were identified with a computational pipeline previously setup (Zhao et al., 2021c). Key steps in the process are as follows. After an all-vs-all reciprocal sequence similarity search for all annotated genomes using DIAMOND (Buchfink et al., 2015), pairwise synteny block detection was performed using MCScanX (Wang et al., 2012). Then the synteny network was clustered using the Infomap algorithm (see “URLs” section). After that, a synteny cluster matrix was obtained, and the number of each species in each cluster was noted, in which the rows and columns correspond to the various species and clusters, respectively. The matrix was then converted into a binary matrix for phylogenetic inference, where 1 denoted the presence of a specific cluster for the species and 0 denoted its absence. This matrix was analyzed using the cor function in R tools to obtain the correlation coefficient between species. The correlation matrix was then plotted and visualized using the R package corrplot (Wei and Simko, 2017). The states of synteny clusters of 16 species were visualized using the UpSetR package (Conway et al., 2017). Clusters shared among Magnoliids-eudicots, Magnoliids-monocots, and eudicots-monocots were further visualized in Cytoscape (Shannon et al., 2003). To select representative clusters of Magnoliids, eudicots, and monocots, the following criteria were set: for Magnoliids, microsyntenic clusters present in four or more species were reserved; for eudicots, microsyntenic clusters present in four or more species were reserved; for monocots, microsyntenic clusters present in three or more species were reserved.

### Genome duplication

We examined genome-wide gene duplications in *L. megaphylla*, *C. kanehirae*, and *L. cubeba* using DupGen_finder (Qiao et al., 2019) with default parameters. The duplicated genes were annotated into five different gene duplication models, including whole-genome duplication (WGD), tandem duplication (TD), proximal duplication (less than 10 gene distance on the same chromosome: PD), transposed duplications (TRD), or dispersed duplications (DSD).

### Secondary metabolite biosynthesis pathways

Protein sequences from sequenced *Lindera* genomes were processed with the Ensemble Enzyme Prediction Pipeline (E2P2) package (version 3.1) (see “URLs” section) to identify putative enzymes. Based on these enzymatic annotations, we then constructed a metabolic pathway database by querying the Plant Metabolic Network (see “URLs” section). The derived pathway database was then validated using SAVI (version 3.1) (Schlapfer et al., 2017) to remove any false positives and redundant pathways, such as non-plant pathway variants, as well as pathways already included in larger pathways. Gene family trees were constructed using IQ-TREE (version 1.6.7) with 1,000 bootstrap replicates. The sequences spanning 2 kb upstream of genes were used to identify transcription factor binding sites (TFBS) in promoters. Putative TF binding sites for suspected promoter sequences were predicted by PlantRegMap (Tian et al., 2019) with q-value ≤ 0.05.

### URLs

SMARTdenovo [https://github.com/ruanjue/smartdenovo];

Pilon [http://github.com/broadinstitute/pilon];

3D-DNA (version 180922) [https://github.com/theaidenlab/3d-dna];

Juicebox (version 1.8) [https://github.com/aidenlab/Juicebox];

LR_Gapcloser (version 1.1) [https://github.com/CAFS-bioinformatics/LR_Gapcloser];

BWA-MEM [https://github.com/lh3/bwa];

HiSat2 (version 2.1.0) [https://github.com/infphilo/hisat2];

RepeatMasker [http://www.repeatmasker.org ];

RepeatModeler [http://www.repeatmasker.org ];

Rfam database (version 9.1) [http://eggnogdb.embl.de ];

InterProScan (version 5.27-66.0) [http://www.ebi.ac.uk/InterProScan];

Infomap algorithm [https://github.com/mapequation/infomap];

Timetree web service (http://www.timetree.org/ );

PMN Ensemble Enzyme Prediction Pipeline (E2P2, version 3.1) (https://gitlab.com/rhee-lab/E2P2);

Plant Metabolic Network (https://www.plantcyc.org).

## Data availability statement

The data presented in the study are deposited both in the NCBI repository with the accession number SRP382804 (https://www.ncbi.nlm.nih.gov/), and in the Genome Warehouse in National Genomics Data Center with the accession number GWHBKHA00000000 (https://ngdc.cncb.ac.cn/gwh).

## Author contributions

J-XL conceived and designed the study; X-CT, J-FG, X-MY, T-LS, SN, S-WZ, Y-TB, Z-CL, and LK prepared the materials and performed related analysis; G-JS provided the specimens; X-CT, J-FG, and J-FM wrote the manuscript; J-FM involved in structuring and polishing the manuscript. All authors contributed to the article and approved the submitted version.
