# Supplementary material for: Unique gene duplications and conserved microsynteny potentially associated with resistance to wood decay in the Lauraceae
Source: Front Plant Sci. 2023 Mar 8;14:1122549. doi: 10.3389/fpls.2023.1122549 (PMC10030967; doi:10.3389/fpls.2023.1122549)
Supplement: Supplementary file 1 [file DataSheet_1.docx]

**Note S1. Estimation of genome size, heterozygosity, and repeat content**

The Nanopore reads were filtered and corrected with CANU (version 1.7) ^1^. Then, *k*-mers were counted using Jellyfish (version 2) ^2^. Finally, GCE (version 1.0.0) was employed to estimate genome size, repeat content and the level of heterozygosity. We identified a total of 73,532,815,926 *k*-mers with *k*-mer size 17, and the peak *k*-mer depth obtained was 99 (**Figure S2**). The genome size was estimated to be ~1.29 Gb. The final cleaned data corresponded to about 99-fold coverage. Repeat and error frequencies were estimated to be 69.81% and 0.11%, respectively. The estimated heterozygosity was at ~0.77%.

**Note S2. Genome sequencing**

**Nanopore sequencing**

For Nanopore sequencing, we prepared PromethION libraries following the Oxford Nanopore 1D Genomic DNA by ligation (SQK-LSK109) PromethION (version GDE_9063_v109_revD_04Jun2018) protocol and sequencing using the nanopore PromethION platform. Finally, a total of 10.336 million raw reads (~179 Gb, roughly 130x coverage of the assembled genome) of s sequencing data with an average read length of 17,297 bp (**Table S1)**.

**Illumina sequencing**

For Illumina sequencing, the 150 bp paired-end (PE) libraries were generated using the NEBNext Ultra II DNA Library Prep Kit with an Illumina HiSeq X Ten platform. Short reads were trimmed with fastp (version 0.19.3) ^3^ to remove adapter sequences, leading and trailing bases with a quality score below 20, and low quality reads with an average per-base-quality of 20 over a 4-bp sliding window were processed with Trimmonmatic. We further removed the nucleotides after pruning less than 70 in length. A total of 1068.502 million raw reads (~160Gb, roughly 120x coverage of the assembled genome) were produced (**Table S1)**.

**Hi-C library preparation and sequencing**

The Hi-C library was prepared following a published study ^4^. After fixing with formaldehyde and lysing the cells, the cross-linked DNA was digested with the the MboI restriction enzyme. The sticky ends of the digested fragments were biotinylated, and ligated to form chimeric junctions that were purified, physically sheared, and enriched for biotin-containing fragments. We completed DNA fragment end repair, adapter attachment, and polymerase chain reaction, and then built the paired end sequencing library. The libraries were sequenced using 2x150 paired-end (PE) with Illumina HiSeq X Ten. A total of 1488.194 million reads (~223 Gb,roughly 170x coverage of the assembled genome) were obtained (**Table S1)**.

**RNA sequencing**

For gene annotation, we extracted RNA from four types of tissue: flower buds, stems, buds and leaves with three biological replicates per tissue. To study gene expression, we then extracted RNA from root, stem bark and tender leaves on the basis of the original four tissues. All frozen tissues were ground with a mortar and pestle. RNA was isolated using the NEBNext Poly(A) mRNA Magnetic Isolation Module and the quality was evaluated on an Agilent 2100 BioAnalyzer. A total of 21 sequencing libraries were prepared using the NEBNext Ultra RNA Library Prep Kit for Illumina. 150-bp PE sequencing was performed in an Illumina HiSeq X Ten machine. Finally, we obtained a total of 597.881 million raw reads (~ 90 Gb, from four tissue types) for gene annotation and 996.020 million raw reads (~ 145 Gb, from seven tissue types) for gene expression study (**Table S1)**.

**Note S3. Genome assembly**

**De novo genome assembly**

The *de novo* assembly was conducted as follows in following steps. First, the primary assembly v0.1 was generated by SMARTdenovo (version 1.0.0) (https://github.com/ruanjue/smartdenovo), assembly v0.2 by WTDBG (version 2.1) ^5^, assembly v0.3 by SMARTdenovo after correction with Canu, assembly v0.4 by WTDBG after correction with Canu. Second, assembly v0.3 (Reasonable assembly size, fewer contigs, highest contig N50) were chosen as optimal for further scaffolding and polishing based on comparison of different primary assemblies. Then the assembly v0.3 was further polished with high quality Illumina reads to produce assembly v1.0 by using three rounds of pilon ^6^.

Then, the Hi-C data were processed together with the above assembly through 3D-DNA (version 180922) pipeline [https://github.com/theaidenlab/3d-dna] to obtain the chromosome-level scaffolds. We mapped the clean Hi-C reads to the assembly v1.0 by Juicer ^7^. The oriented sacffolds were inspected and manually corrected using Juicebox (version 1.8) [https://github.com/aidenlab/Juicebox] and re-scaffolded by using 3D-DNA. The new scaffolds were further manually adjusted with Juicebox including removing erroneous inserts, adjusting the bound, order and mis-join to correct the visible errors as much as possible.

After correcting the scaffold, the genome gaps were closed twice using LR_Gapcloser (version 1.1) [https://github.com/CAFS-bioinformatics/LR_Gapcloser] with raw Nanopore long reads. In order to improve the single-base accuracy, we used pilon program [http://github.com/broadinstitute/pilon] to run four rounds polish with filtered Illumina short reads. Then we removed low-quality contigs, including scattered contigs less than 5000bp in length, low average coverage ratio (less than 10X) or high non-coverage ratio (more than 60%) contigs, and contigs with identity cutoff of higher than 0.91. Whereafter, we aligned the filtered assembly with the database by blastn (version 2.2.28+), and no contamination was found with other species in the final assembly v1.1. We revealed a low heterozygosity rate of ~0.53% and a single base error rate of ~0.02% by mapping the Illumina reads to the final assembly with BWA-MEM [https://github.com/lh3/bwa].

**Transcriptome assembly**

For transcriptome assembly, we obtained a total of 594,501 raw RNA-seq reads from four tissues including flower buds, stems, buds and leaves. Illumina adapters and low-quality reads were removed from the raw reads using Trimmomatic (version 0.36) ^8^ and Cutadapt (version 1.13) ^9^. We aligned the filtered reads to the *Lindera* genome by HiSat2 after evaluating the base quality with FastQC (version 0.11.6) (<https://www.bioinformatics.babraham.ac.uk/projects/fastqc/>).

Reference genome guided transcriptome assemblies were prepared with Trinity (version 2.0.6) ^10^ and StringTie (version 1.3.5) ^11^, De novo assemblies were independently generated using Trinity. We then combined the transcriptome assemblies and reduced the redundant sequences by using CD-HIT. Finally, 115,454 unique transcripts were obtained and used as EST (expressed sequencing tag) evidence for gene prediction.

**Evaluation of assembly quality**

We evaluated the completeness and continuity of the genome from several aspects. First, the reference assembly obtained a qualified LTR Assembly Index (LAI) score of 12.14 ^12^. Second, we mapped all clean Illumina reads to the final assembly, a high sequence coverage of 96.2% and high reads mapping rate of 99.2% were obtained with BWA-MEM [https://github.com/lh3/bwa]. A sequence coverage of 81.3% and higher reads mapping rate of 99.96%was observed for mapping Nanopore long reads to the final assembly using minimap2 (version 2.11-r797) ^13^. A total 87.0 % of the sequences could be mapped to the genome assembly by HiSat2 (version 2.1.0) [https://github.com/infphilo/hisat2] using RNA-seq data from four tissues (flower buds, stems, buds, leaves). Finally, in BUSCO assessment ^14^, a total of 90.7% completed genes were obtained by mapping 1,440 conserved plant orthologous genes to the assembled genome. To assess the correctness of our assembly, we mapped the Illumina reads from genome sequencing to the final assembly, then a heterozygosity of ~0.53% and a single base error rate of ~0.020% based on SNPs identified with SAMtools ^15^ were obtained. In additon, there was no obvious GC bias in the Nanopore sequencing data and the Illumina sequencing data. The chromatin interactions were shown by Hi-C reads mapped onto the final assembly by Juicer [https://github.com/aidenlab/juicer].

**Note S4. Gene structural and functional annotation**

We used MAKER pipeline (version 2.31.9) ^16^ to predicted protein-coding genes in the *Lindera* genome through a combination of ab initio and evidence-based which includes protein-homology and EST-based prediction methods. EST was aligned to the repeat masked reference genome using BlastN and TblastX from BLAST (v2.2.28+). For evidence-based gene prediction, we clustered and cleaned the protein sequences from the genome of *Amborella trichopoda*, *Cinnamomum kanehirae*, *Oryza sativa* and *Arabidopsis thaliana*, and then generated protein homology evidence for gene prediction by CD-HIT (95% identity and 95% coverage) ^17^. We aligned EST data to the repeat-masked genome by combining BLASTN and TBLASTX and aligned protein data to the repeat-masked genome by BLASTX. The alignments were further polished using Exonerate (version 2.4.0) ^18^. Then the evidence generated by the methods mentioned above were integrated using the MAKER2 package within AUGUSTUS ^19,20^ to produce a consensus gene set. AED (Annotation Edit Distance) scores which used to evaluate the quality of gene prediction were calculated for all the predicted genes. Finally, we obtained a high quality annotated gene set by removing genes that had abnormal and too short (<=50 aa) ORFs (open reading frames). Transfer RNA (tRNA) genes were predicted using tRNAscan-SE (version 1.3.1) ^21^. with eukaryote parameters. Ribosomal RNA (rRNA) were identified using RNAMMER (version 1.2) ^22^. Other ncRNAs were found by searching against the Rfam database (version 9.1) [http://eggnogdb.embl.de] and BLASTN (version 2.2.28+).

Functional annotation of the predicted genes based on sequence similarity searches by performing blat (version 36) ^23^ against seven protein databases: SwissProt protein database ^24^, the Translated European Molecular Biology Laboratory (TrEMBL) database ^24^, the NCBI non-redundant protein database (NR) (https://www.ncbi.nlm.nih.gov/) , the protein families database (Pfam) ^25^, the evolutionary genealogy of genes: Non-supervised Orthologous Groups database (eggnog), the KEGG Orthology database (KO) ^26^ and the GO (Gene Ontology) database ^27^, with 30% minimum identity and 1e-05 E-value cutoff. The domains and motifs were annotated using InterProScan (version 5.27-66.0) [http://www.ebi.ac.uk/InterProScan] with default parameters. The completeness of genome annotation was assessed using BUSCO.

In total, 89.1% of the 32,586 protein-coding genes (29,034 genes) were annotated by at least one protein database with the following results: the NCBI non-redundant protein database (NR) (see “URLs” section) (89.0%), the Swiss-Prot protein database ^24^ (60.4%), the Translated European Molecular Biology Laboratory (TrEMBL) database ^24^ (87.9%), the protein families database (Pfam) ^25^ (76.5%), the Kyoto Encyclopedia of Genes and Genomes, Orthology (KO) database ^26^ (37.1%), the Evolutionary Genealogy of Genes: Non-supervised Orthologous Groups (eggNOG) database ^28^ (84.8%), and the Gene Ontology (GO) database (59.7%) ^27^ (**Table S7**).

**Note S5.** **Transposable element and other repeat annotation**

We constructed a *Lindera* repeat library using a de nove approach by applying RepeatModeler (version 1.0.10) (http://www.repeatmasker.org) and subsequently the outputs from RepeatModeler were used for further identification of transposable elements (TEs) and other repeats by RepeatMasker (version 4.0.7, rmblast-2.2.28) (http://www.repeatmasker.org).

LTRharvest ^29^ and LTRdigest ^30^ were both used to predict the LTR-RTs in the assembled genome. It was required that a candidate LTR-RT was separated by 1 to 15 kb from other candidates and flanked by a pair of putative LTRs, which ranged from 100 bp to 3000 bp, with similarity > 80%. LTR-RT included gag domain, protease domain, reverse transcriptase (RT) domain and integrase domain, which was considered as an intact LTR-RT. Unpaired LTRs including solo-LTRs and truncated LTR-RTs, were identified based on sequence similarity to LTRs of intact LTR-RTs in each genome. We determined LTR homologies using BLASTN analysis with E-value cutoff of 1e-10, 90% overlap in length and 90% identity. We extracted 3kb sequences from both upstream and downstream of each detected LTR paralog and compared them with Gag-Pol protein sequences in the Gypsy database 2.0 ^31^ using TBLASTN. If at least 50% of any Gag-Pol sequence was covered by the flanking sequences with an identity > 30% and an E-value cutoff of 1e-8, the corresponding LTR was excluded from the solo-LTR list. The LTR homologies lacking any Gag-Pol homology in both the upstream and downstream sequences are considered to be solo-LTRs and LTRs with Gag-Pol sequences on one side of flanking sequences are considered to be truncated LTR-RTs.

We compared the 5’-LTR sequences of all LTR-RTs to each other with blastn using Silix to further understand the LTR-RT relationship. If two LTRs covered at least 70% of their lengths and the identity between them of at least 60%, they are assigned to the same cluster. Solo-LTRs (*S*) and truncated LTR-RTs (*T*) were also mapped to the same cluster containing 5’ LTRs from the most similar intact LTR-RTs (*I*). In addition, the ratio of solo-LTR-RTs and truncated LTR-RTs to intact LTR-RTs (*S*:*I*;*T*:*I*) and their sums were evaluated to study the removal rate of LTR-RTs over the past several million years. LTR-RT deletions was then evaluated by using proportions of clusters with *S*:*I* values greater than 3. We also performed an LTR-RTs analysis of 17 other species studied in phylogenetic analysis for interspecific comparisons.

Repetitive sequences composed 66.98% (849.66 Mb) of the *L. megaphylla* genome assembly (**Table 1** and **Table S9**), of which 73.37% were annotated as transposable elements (TEs). Long terminal repeat-retrotransposons (LTR-RTs) were the most abundant TEs, occupying 40.25% of the genome, among which *Gypsy* and *Copia* accounted for 26.70% and 12.96%, respectively (**Table S9**). By comparing *L. megaphylla* to other magnoliids with respect to LTR-RT accumulation, we found that LTR-RT accumulation was highest in *Liriodendron chinense* (solo-LTR, [S]+truncated LTR-RT [T]+ intact LTR-RT [I] = 228,97), followed by *Litsea cubeba* (S+T+I =148,04), and *L. megaphylla* (S+T+I = 141,87), whereas LTR-RT accumulation was lowest in *Cinnamomum kanehireae* (S+T+I = 32,94) (**Figure S7** and **Table S10**). Proportionately, the *L. chinense* genome is the largest (1.7 Gb) ^32^, followed by *L. cubeba* (1.26 Gb) ^33^ and *L. megaphylla* (1.25 Gb), while the genome of *C. kanehireae* is the smallest (730 Mb) ^34^. Therefore, we suggest that LTR-RTs are key contributors to genomic size variation in Magnoliids.

**Note S6. Gene families and phylogenetic inference**

To determine the phylogenetic relationships among magnoliids, we used the Orthofinder to identify the gene families between the *Lindera* and five other magnoliids (*Persea Americana*, *Cinnamomum kanehirae*, *Liriodendron chinense*, *Piper nigrum*, *Litsea cubeba*), seven eudicots (*Arabidopsis thaliana*, *Populus trichocarpa*, *Cajanus cajan*, *Coffea* *canephora*, *Solanum lycopersicum*, *Aquilegia coerulea*), four monocots (*Sorghum bicolor*, *Oryza sativa*, *Musa acuminate*, *Zostera marina*), one basal angiosperms *Nymphaea colorata* and *Amborella trichopoda* as the outgroup , a total of 34,888 orthogroups including 112 orthologous single-copy gene families and 885 low-copy gene families were identified based on effective database sizes of all versus all BLASTP with an E-value of 10 -5. Amino acid sequence alignment was performed on these low-copy genes using MUSCLE by default settings and filtered using a trimAI (version 1.2) (trimal -gt 0.8 -st 0.001 -cons 60) ^35^.

Phylogenetic trees were constructed by three methods including concatenation, coalescent, and microsynteny-based approaches ^36^. For the concatenation-based approach, the maximum likelihood tree was constructed using these concatenated low-copy amino acid sequences with IQ-TREE (version 1.6.7) ^37^ employing the best-fit model (-m JTT+F+R5) with ultrafast bootstrapping (-bb 1000) . For the coalescent-based approach, gene trees of 855 low-copy gene family were inferred by IQ-TREE. Next, we removed low support branches that less than 55% using Newick utilities and these gene trees were used to construct species trees by ASTRAL-pro ^38^ and quartet support of each node was estimated for this coalescent tree. Finally, the method based on microcollinearity, which included two steps. First, all-by-all protein alignment of the whole genome was performed using BLAST, and then the pairwise synteny blocks were identified using MCScanX. Then microsyntenic clusters were detected using Infomap algorithm [https://github.com/mapequation/infomap] within the map equation framework (Rosvall and Bergstrom, 2008). We used the two-level partitioning mode with ten trials.

Next ,we used the MCMCTree of the PAML v4.9h ^39^ to estimate the divergence time of *Lindera* from the other plants based on 112 single-copy orthologous genes. The species tree and the concatenated nucleotide aligenments of these single-copy genes as input files for MCMCtree. The dating was calibrated according to the TimeTree web service (http://www.timetree.org/) by placing soft bounds at four split nodes as constraints for calibrating tree age: (1) the *A. trichopoda* node (173-199 Mya), (2) *L. chinense* (117-130 Mya), (3) *O. sativa*-*S. bicolor* (42-52 Mya) and (4) *P. trichocarpa*-*A. thaliana* (98-177 Mya).

By separating Standard Deviation of gene families with <100 from >=100 and which were shared among two species at least, we screened out 13,398 gene families. Then we used CAFÉ (version 4.1) ^40^ with 0.05 p-value cutoff to inferred expansion and contraction of those gene families. Finally, we detected 1303 expanded and 1822 contracted gene families in *L. megaphylla*.

**Note S7.** **Whole-genome duplication in** **Lauraceae**

Synteny blocks containing at least five genes found by MCScanX were used to detect WGD events in *Lindera*. TBtools was used to construct dot plots of orthologous and paralogous blocks identified using MCScanX, as well as synteny plots among genomic regions of *L. megaphylla*, *C. canephora*, and *Amborella trichopoda* ^41^. We used KaKs_Calculator (version 2.0) ^42^ under a Yang-Nielsen (YN) model to calculate the *K_s_* values of paralogous (within the *L. megaphylla*, *C. kanehirae*, *L. cubeba*, *L. chinense* genomes, and *C. canephora*) and orthologous (between *L. megaphylla* and *C. canephora*) syntenic blocks. The WGD time of Lauraceae was estimated with *C. canephora*-Lauraceae divergence time (mean: 152 Mya) as an age constraint. *K_s_* peaks of *C. canephora* vs. *L. megaphylla* syntenic orthologs allowed for the calculation of *K_s_* per year (r) following r = *K_s_*/(2×(divergence time)). The same r value was applied to calculate the time of WGD events for Lauraceae.

Syntenic depth ratios of 4:4, 4:3, and 4:1 were determined in the comparisons of *Lindera*-*Lindera*, *L. megaphylla*- *C. canephora*, and *L. megaphylla*- *A. trichopoda*, respectively (**Figure S12A–12C** and **Figure S13A**), indicating that two whole-genome duplication (WGD) events occurred in the *L. megaphylla* genome. Moreover, the two apparent and identical *K_s_* (homologue synonymous substitutions per site) peaks at approximately 0.56 and 0.8 shared among the *L. cubeba*, *C. kanehirae*, and *L. megaphylla* genomes further supported that the Lauraceae experienced two polyploidization events before species divergence within the family (**Figure S12D**). The two polyploidization events of Lauraceae were estimated to have occurred about 57 Mya and 76 Mya (**Figure 1A**).

The distribution of one *K_s_* peak in *L. chinensis* and *Magnolia biondii*, as well as the syntenic depth ratios of 2:2 and 2:3 in *L. chinensis*-*L. chinensis* and *L. chinensis*- *C. canephora*, suggest that only one WGD event has occurred in the Magnoliaceae (**Figure S13B** and **13C**), which is consistent with the WGD results of the *Liriodendron* and *M. biondii* genomes ^32,43^. Previous reports have investigated whether the ancient WGD of Laurales is shared with the Magnoliales. Analysis of the *C. kanehirae*, *L. chinensis*, and *L. cubeba* genomes suggested that the ancient WGD event may have occurred before the divergence of Laurales and Magnoliales ^32,33^, while the *Chimonanthus praecox* ^44^ genome did not suggest a common WGD event. In our study, we found syntenic depth ratios of 4:2 in *L. megaphylla*-*L. chinensis* and 4:2 in *L. megaphylla-M. biondii* (**Figure S14)**. We also found that the *K_s_* differentiation peaks of *L. megaphylla*-*M. biondii* and *L. megaphylla*-*L. chinensis* were larger than the *K_s_* peak in *M. biondii* and *L. chinensis*. Together, these results suggest that the Laurales and Magnoliales may not share a common WGD event.

**Note S8. *TPS* genes in Lauraceae**

Biosynthesis and accumulation of terpenoids are also important for WDR ^45,46^. Terpene synthases (TPSs) play an essential role in the synthesis of volatile terpenes and are key enzymes for the generation of terpenoid diversity ^47^. Six *TPS* subfamilies, including *TPS-a*, *TPS-b*, *TPS-c*, *TPS-e/f*, and *TPS-g*, were annotated in Lauraceae, among which *TPS-b* was the most abundant, followed by *TPS-a* (**Figure S24**). *TPS-a* was mainly involved in sesquiterpene biosynthesis, and *TPS-b* was mainly involved in monoterpene biosynthesis (**Figure S24**). We found that TD/PD duplication in Lauraceae caused large-scale expansion of the *TPS-a* and *TPS-b* subfamilies (**Figure S24**). The contribution of TD/PD duplication to *TPS* genes was much higher in Lauraceae than in other species, and the *TPS*-TD/PD genes were the most widely distributed in *L. megaphylla* (85.71%), followed by *C. kanehirae* (81.54%) and *L. cubeba* (77.53%) (**Figure S25** and **Table S19**). A large-scale conserved TD/PD TPS gene cluster was found among Lauraceae species (**Figure S26a**). We found that a TD/PD cluster including *TPS-a*, *TPS-b*, and *TPS-g* subfamily genes was shared and conserved among Lauraceae species (**Figure S26a** and **26b**). Subsequently, TD/PD occurred in the *TPS-a* and *TPS-b* branches of Lauraceae, forming different clades (**Figure S26b**). Species-specific TD/PD duplication also occurred in *TPS-a*, *TPS-b*, and *TPS-g* (**Figure S26b**). Microsynteny analysis revealed a Lauraceae-specific cluster (C28) associated with the *TPS-b* subfamily of diterpenes (**Figure S26c**). Moreover, a *TPS* biosynthetic gene cluster (BGC) containing several *TPS-a* genes was identified, including sesquiterpene genes and a mevalonate kinase (*MVK*) gene. In this BGC, we found significant expansions in *TPS-a* genes by TD/PD before and after Lauraceae speciation (**Figure S26d** and **26e**). In terms of terpenoid biosynthesis, we mainly studied the *TPS* genes. Although the *TPS* genes in Lauraceae do not have the obvious specificity of the above-mentioned enzyme genes, they show remarkable TD/PD duplications compared with other species. Therefore, TD/PD duplications play an extremely important role in promoting the biosynthesis and accumulation of terpenoid compounds, and thus may improve the WDR of Lauraceae species.

**References**

1 Koren, S. *et al.* Canu: scalable and accurate long-read assembly via adaptive *K*-mer weighting and repeat separation. *Genome Research* **27**, 722-736. <https://doi.org/10.1101/gr.215087.116> (2017).

2 Marçais, G. & Kingsford, C. A fast, lock-free approach for efficient parallel counting of occurrences of *K*-mers. *Bioinformatics* **27**, 764-770. <https://doi.org/10.1093/bioinformatics/btr011> (2011).

3 Chen, S., Zhou, Y., Chen, Y. & Gu, J. fastp: an ultra-fast all-in-one FASTQ preprocessor. *Bioinformatics* **34**, i884-i890. <https://doi.org/10.1093/bioinformatics/bty560> (2018).

4 Duan, Z. *et al.* A three-dimensional model of the yeast genome. *Nature* **465**, 363-367. <https://doi.org/10.1038/nature08973> (2010).

5 Ruan, J. & Li, H. Fast and accurate long-read assembly with wtdbg2. *Nature Methods* **17**, 155-158. <https://doi.org/10.1038/s41592-019-0669-3> (2020).

6 Walker, B. J. *et al.* Pilon: an integrated tool for comprehensive microbial variant detection and genome assembly improvement. *PloS One* **9**, e112963. <https://doi.org/10.1371/journal.pone.0112963> (2014).

7 Durand, N. C. *et al.* Juicer Provides a One-Click System for Analyzing Loop-Resolution Hi-C Experiments. *Cell Systems* **3**, 95-98. <https://doi.org/10.1016/j.cels.2016.07.002> (2016).

8 Miele, V., Penel, S. & Duret, L. Ultra-fast sequence clustering from similarity networks with SiLiX. *BMC Bioinformatics* **12**, 116. <https://doi.org/10.1186/1471-2105-12-116> (2011).

9 Martin, M. Cutadapt removes adapter sequences from high-throughput sequencing reads. *EMBnet. Journal* **17**, 10-12. <https://doi.org/10.14806/ej.17.1.200> (2011).

10 Grabherr, M. G. *et al.* Full-length transcriptome assembly from RNA-Seq data without a reference genome. *Nature Biotechnology* **29**, 644-652. <https://doi.org/610.1038/nbt.1883>, <https://doi.org/10.1038/nbt.1883> (2011).

11 Pertea, M. *et al.* StringTie enables improved reconstruction of a transcriptome from RNA-seq reads. *Nature Biotechnology* **33**, 290-295. <https://doi.org/10.1038/nbt.3122> (2015).

12 Ou, S., Chen, J. & Jiang, N. Assessing genome assembly quality using the LTR Assembly Index (LAI). *Nucleic Acids Research* **46**, e126. <https://doi.org/10.1093/nar/gky730> (2018).

13 Li, H. Minimap2: pairwise alignment for nucleotide sequences. *Bioinformatics* **34**, 3094-3100., <https://doi.org/10.1093/bioinformatics/bty191> (2018).

14 Simão, F. A., Waterhouse, R. M., Ioannidis, P., Kriventseva, E. V. & Zdobnov, E. M. BUSCO: assessing genome assembly and annotation completeness with single-copy orthologs. *Bioinformatics* **31**, 3210-3212. <https://doi.org/10.1093/bioinformatics/btv351> (2015).

15 Li, H. & Durbin, R. Fast and accurate short read alignment with Burrows-Wheeler transform. *Bioinformatics* **25**, 1754-1760. <https://doi.org/10.1093/bioinformatics/btp324> (2009).

16 Holt, C. & Yandell, M. MAKER2: an annotation pipeline and genome-database management tool for second-generation genome projects. *BMC Bioinformatics* **12**, 491. <https://doi.org/10.1186/1471-2105-12-491> (2011).

17 Fu, L., Niu, B., Zhu, Z., Wu, S. & Li, W. CD-HIT: accelerated for clustering the next-generation sequencing data. *Bioinformatics* **28**, 3150-3152. <https://doi.org/10.1093/bioinformatics/bts565> (2012).

18 Slater, G. S. C. & Birney, E. Automated generation of heuristics for biological sequence comparison. *BMC Bioinformatics* **6**, 31. <https://doi.org/10.1186/1471-2105-6-31> (2005).

19 Keller, O., Kollmar, M., Stanke, M. & Waack, S. A novel hybrid gene prediction method employing protein multiple sequence alignments. *Bioinformatics* **27**, 757-763. <https://doi.org/10.1093/bioinformatics/btr010> (2011).

20 Stanke, M., Diekhans, M., Baertsch, R. & Haussler, D. Using native and syntenically mapped cDNA alignments to improve de novo gene finding. *Bioinformatics* **24**, 637-644. <https://doi.org/10.1093/bioinformatics/btn013> (2008).

21 Lowe, T. M. & Eddy, S. R. tRNAscan-SE: a program for improved detection of transfer RNA genes in genomic sequence. *Nucleic Acids Research* **25**, 955-964. <https://doi.org/10.1093/nar/25.5.955> (1997).

22 Lagesen, K. *et al.* RNAmmer: consistent and rapid annotation of ribosomal RNA genes. *Nucleic Acids Research* **35**, 3100-3108. <https://doi.org/10.1093/nar/gkm160> (2007).

23 Kent, W. J. BLAT-the BLAST-like alignment tool. *Genome Research* **12**, 656-664. <https://doi.org/10.1101/gr.229202> (2002).

24 Bairoch, A. & Apweiler, R. The SWISS-PROT protein sequence database and its supplement TrEMBL in 2000. *Nucleic Acids Research* **28**, 45-48. <https://doi.org/10.1093/nar/28.1.45> (2000).

25 Finn, R. D. *et al.* Pfam: the protein families database. *Nucleic Acids Research* **42**, D222–D230. <https://doi.org/10.1093/nar/gkt1223> (2014).

26 Kanehisa, M. & Goto, S. KEGG: kyoto encyclopedia of genes and genomes. *Nucleic Acids Research* **28**, 27-30. <https://doi.org/10.1093/nar/28.1.27> (2000).

27 Consortium, G. O. The Gene Ontology (GO) database and informatics resource. *Nucleic Acids Research* **32**, D258-D261. <https://doi.org/10.1093/nar/gkh036> (2004).

28 Huerta-Cepas, J. *et al.* Fast genome-wide functional annotation through orthology assignment by eggNOG-mapper. *Molecular Biology and Evolution* **34**, 2115-2122. <https://doi.org/10.1093/molbev/msx148> (2017).

29 Ellinghaus, D., Kurtz, S. & Willhoeft, U. LTRharvest, an efficient and flexible software for de novo detection of LTR retrotransposons. *BMC Bioinformatics* **9**, 18. <https://doi.org/10.1186/1471-2105-9-18> (2008).

30 Steinbiss, S., Willhoeft, U., Gremme, G. & Kurtz, S. Fine-grained annotation and classification of de novo predicted LTR retrotransposons. *Nucleic Acids Research* **37**, 7002-7013. <https://doi.org/10.1093/nar/gkp759> (2009).

31 Llorens, C. *et al.* The Gypsy Database (GyDB) of mobile genetic elements: release 2.0. *Nucleic Acids Research* **39**, D70-D74. <https://doi.org/10.1093/nar/gkq1061> (2011).

32 Chen, J. *et al.* *Liriodendron* genome sheds light on angiosperm phylogeny and species–pair differentiation. *Nature Plants* **5**, 18-25. 10.1038/s41477-018-0323-6 (2019).

33 Chen, Y.-C. *et al.* The *Litsea* genome and the evolution of the laurel family. *Nature Communications* **11**, 1675. <https://doi.org/10.1038/s41467-020-15493-5> (2020).

34 Chaw, S.-M. *et al.* Stout camphor tree genome fills gaps in understanding of flowering plant genome evolution. *Nature Plants* **5**, 63-73. 10.1038/s41477-018-0337-0 (2019).

35 Capella-Gutiérrez, S., Silla-Martínez, J. M. & Gabaldón, T. trimAl: a tool for automated alignment trimming in large-scale phylogenetic analyses. *Bioinformatics* **25**, 1972-1973. <https://doi.org/10.1093/bioinformatics/btp348> (2009).

36 Zhao, T. *et al.* Whole-genome microsynteny-based phylogeny of angiosperms. *Nature Communications* **12**, 3498. <https://doi.org/10.1038/s41467-021-23665-0> (2021).

37 Nguyen, L.-T., Schmidt, H. A., von Haeseler, A. & Minh, B. Q. IQ-TREE: a fast and effective stochastic algorithm for estimating maximum-likelihood phylogenies. *Molecular Biology and Evolution* **32**, 268-274. <https://doi.org/10.1093/molbev/msu300> (2014).

38 Zhang, C., Scornavacca, C., Molloy, E. K. & Mirarab, S. ASTRAL-Pro: quartet-based species-tree inference despite paralogy. *Molecular Biology and Evolution* **37**, 3292-3307. <https://doi.org/10.1093/molbev/msaa139> (2020).

39 Yang, Z. PAML 4: phylogenetic analysis by maximum likelihood. *Molecular Biology and Evolution* **24**, 1586-1591. <https://doi.org/10.1093/molbev/msm088> (2007).

40 De Bie, T., Cristianini, N., Demuth, J. P. & Hahn, M. W. CAFE: a computational tool for the study of gene family evolution. *Bioinformatics* **22**, 1269-1271. 10.1093/bioinformatics/btl097 (2006).

41 Chen, C. *et al.* TBtools: an integrative toolkit developed for interactive analyses of big biological data. *Molecular Plant* **13**, 1194-1202. <https://doi.org/10.1016/j.molp.2020.06.009> (2020).

42 Wang, D., Zhang, Y., Zhang, Z., Zhu, J. & Yu, J. KaKs_Calculator 2.0: a toolkit incorporating gamma-series methods and sliding window strategies. *Genomics Proteomics Bioinformatics* **8**, 77-80. <https://doi.org/10.1016/s1672-0229(10)60008-3> (2010).

43 Dong, S. *et al.* The genome of *Magnolia biondii* Pamp. provides insights into the evolution of Magnoliales and biosynthesis of terpenoids. *Horticulture Research* **8**, 38. <https://doi.org/10.1038/s41438-021-00471-9> (2021).

44 Shang, J. *et al.* The chromosome-level wintersweet (Chimonanthus praecox) genome provides insights into floral scent biosynthesis and flowering in winter. *Genome Biology* **21**, 1-28. <https://doi.org/10.1186/s13059-020-02088-y> (2020).

45 Kawaguchi, H. *et al.* Several antifeedants from *Phellodendron amurense* against *Reticulitermes* *speratus*. *Agricultural and Biological Chemistry* **53**, 2635-2640. <https://doi.org/10.1080/00021369.1989.10869702> (1989).

46 Park, I.-K., Lee, H.-S., Lee, S.-G., Park, J.-D. & Ahn, Y.-J. Antifeeding activity of isoquinoline alkaloids identified in *Coptis japonica* roots against *Hyphantria cunea* (Lepidoptera: Arctiidae) and *Agelastica coerulea* (Coleoptera: Galerucinae). *Journal of Economic Entomology* **93**, 331-335. <https://doi.org/10.1603/0022-0493-93.2.331> (2000).

47 Cheng, A. X. *et al.* Plant terpenoids: biosynthesis and ecological functions. *Journal of Integrative Plant Biology* **49**, 179-186. <https://doi.org/10.1111/j.1744-7909.2007.00395.x> (2007).

**
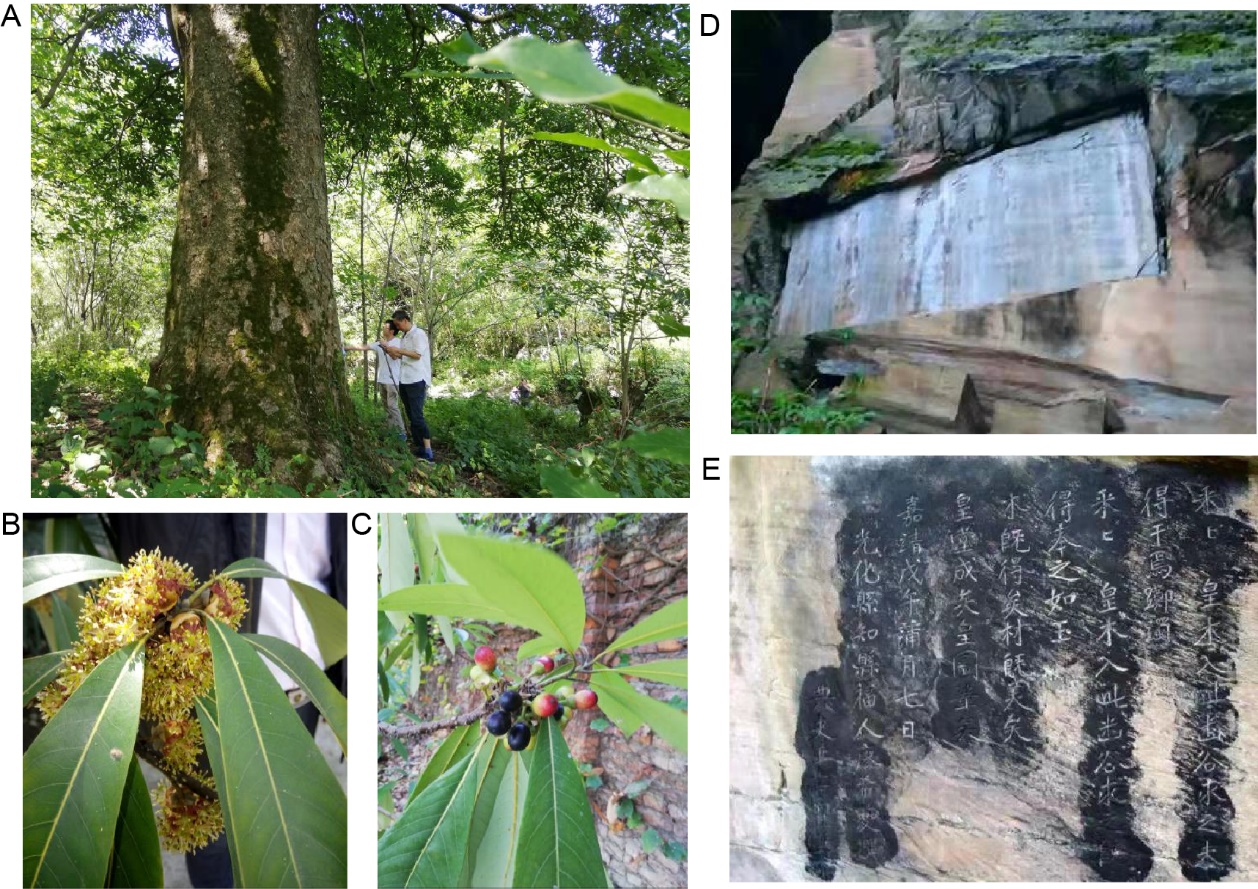
**

**Figure S1.** Images of the tree **(A)**, flowers **(B)**, and fruits **(C)** of the *Lindera megaphylla* and the cliff carvings **(D, E)** (carved at 1522-1566, Qing Dynasty) describing Nanmu (including *Lindera megaphylla* and probably several other Lauraceae trees) were harvested and transported to Beijing (the capital) for royal palace buildings, from the sampling site.

**
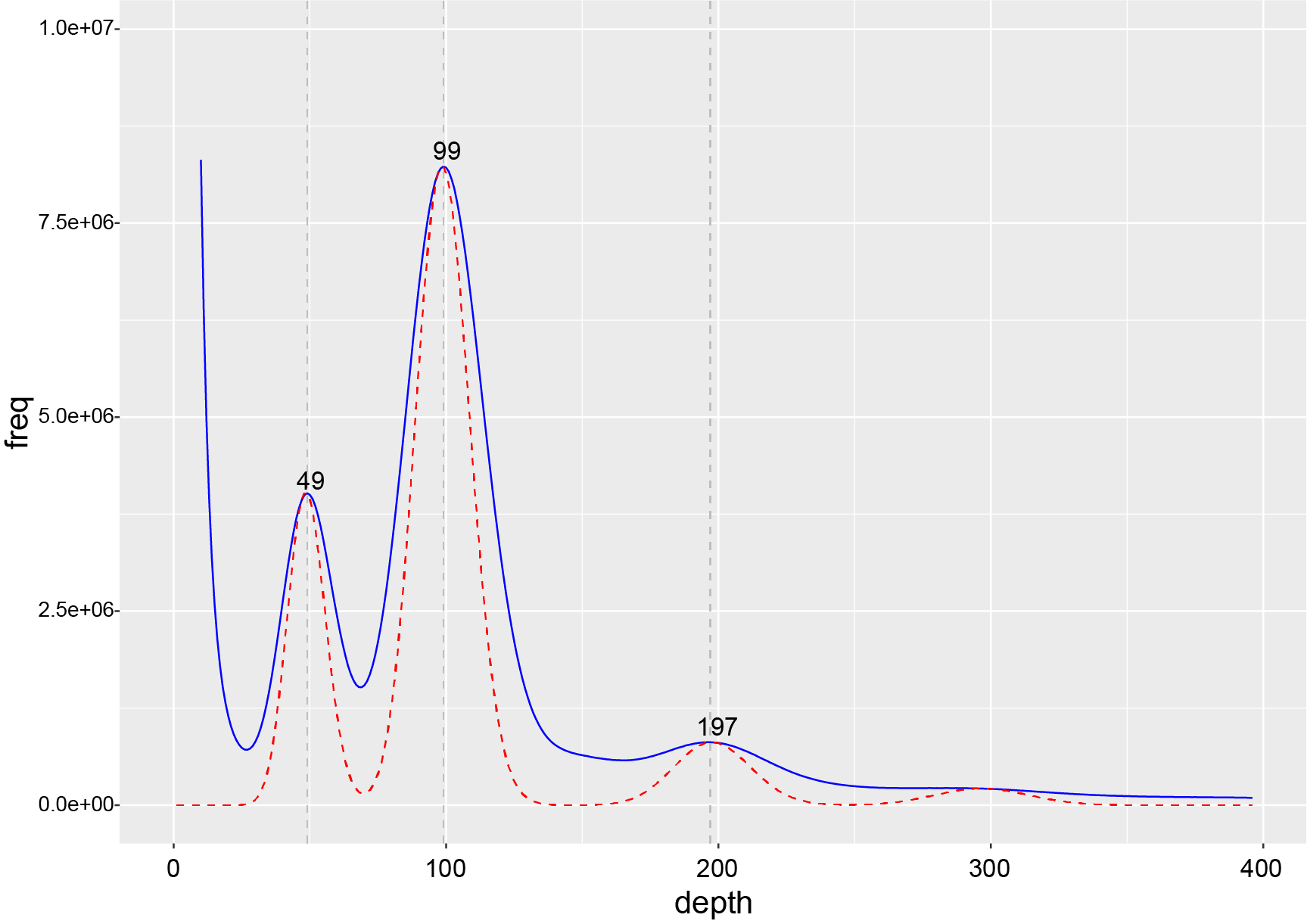
**

**Figure S2. The *k*-mer frequency distribution of sequencing reads at *k*-mer size of 17.** According to the distribution, we estimated the genome size of *L. megaphylla* is about 1.29 Gb, with a heterozygosity rate of 0.5% and a repeat proportion of 60%.


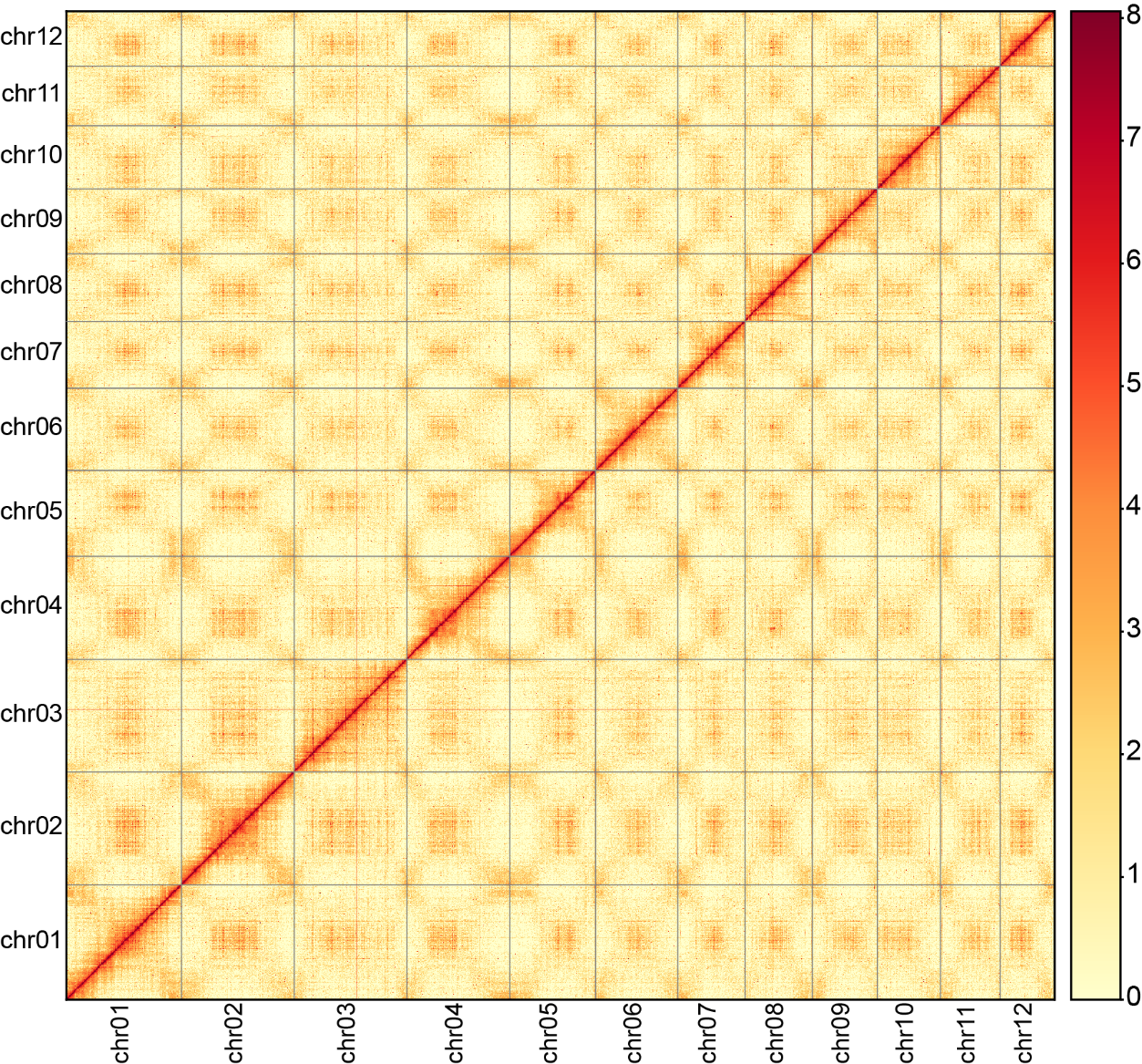


**Figure S3.** **Interchromosomal Hi-C contact map of *L. megaphylla*.** Bright red indicates higher interaction probabilities.


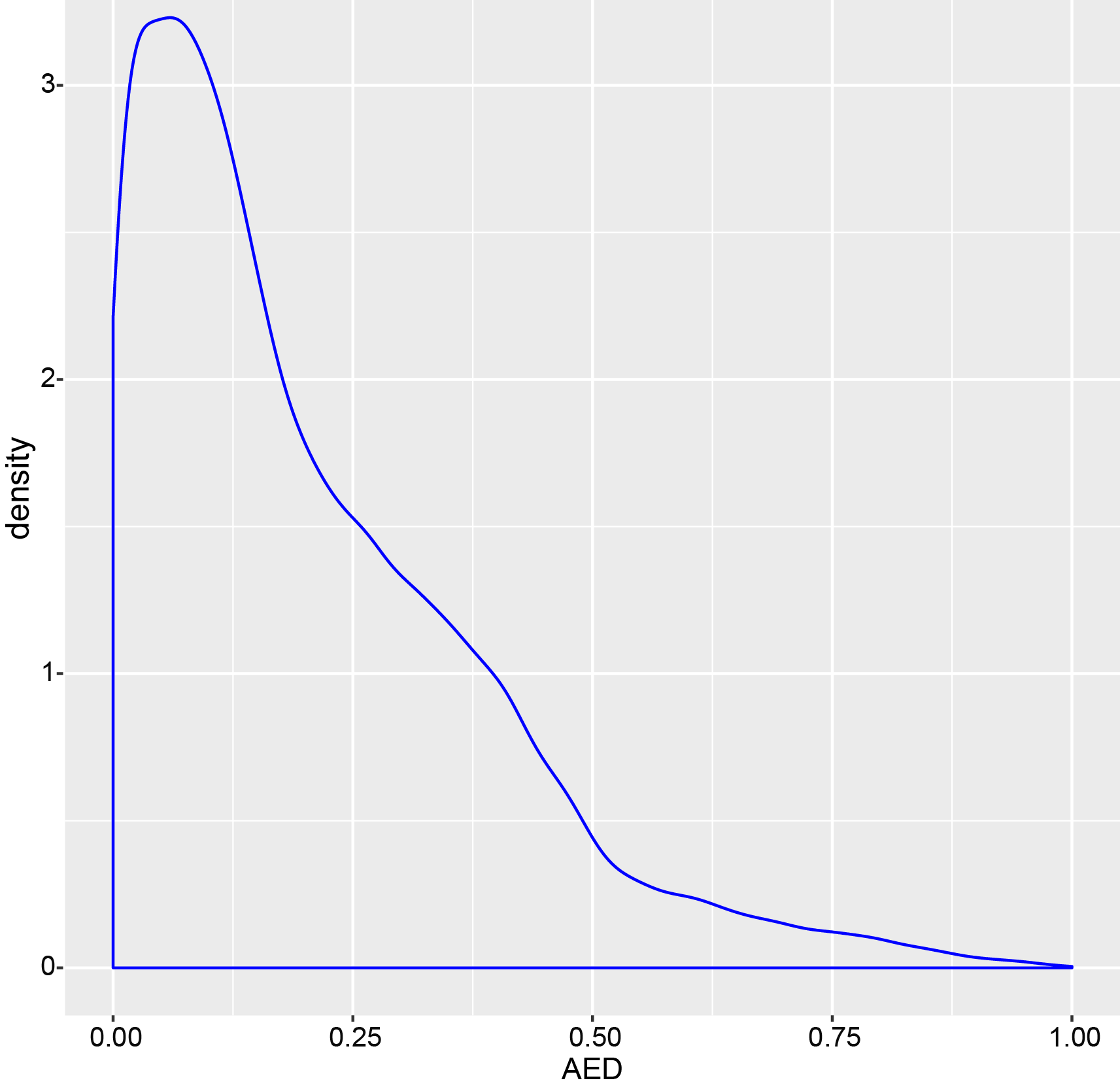


**Figure S4.** **Distribution of AED (annotation edit distance) scores from gene prediction.** AED = 0 indicates perfect agreement between the gene prediction and the transcript and protein evidence; AED = 1 indicates no evidence support for annotation.


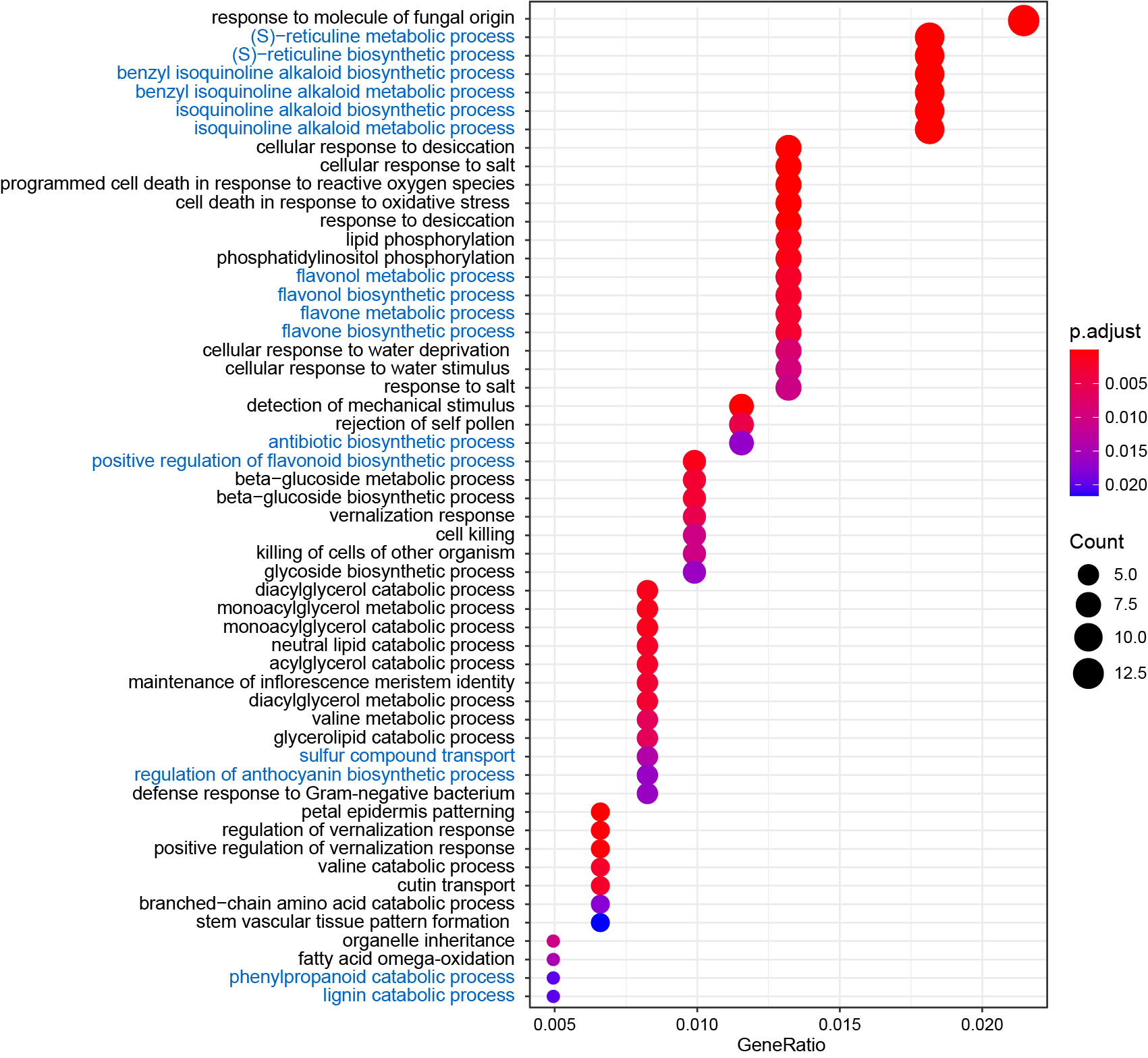


**Figure S5. GO enrichment analysis of expanded gene families in Lauraceae.** GO terms were kept with the adjusted q-values < 0.05. Blue letters indicate terms related to wood decay resistance.


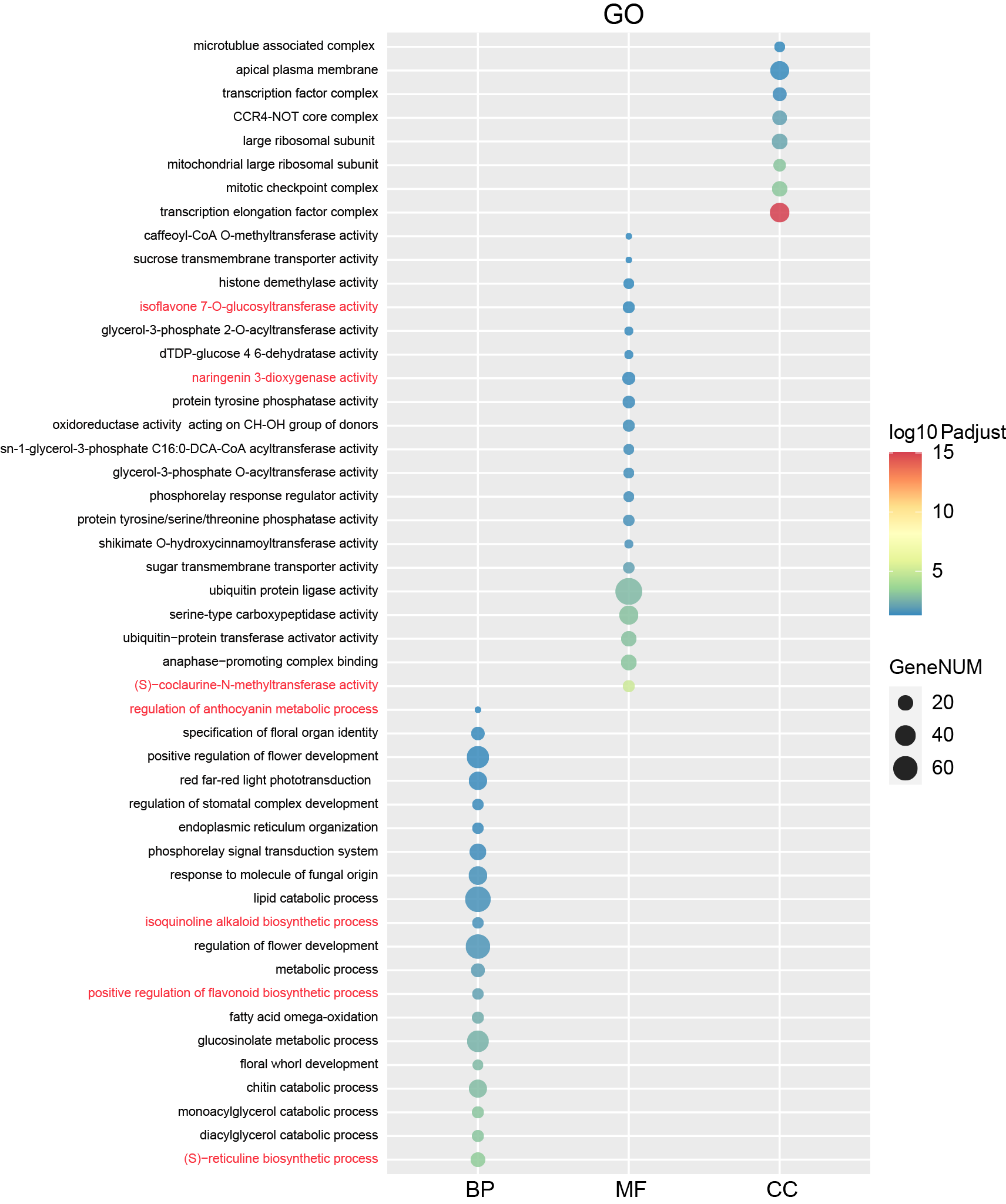


**Figure S6. KEGG and GO enrichment analysis of expanded gene families in *L. megaphylla*.** GO terms were kept with the adjusted q-values < 0.05. Red letters indicate terms related to wood decay resistance.


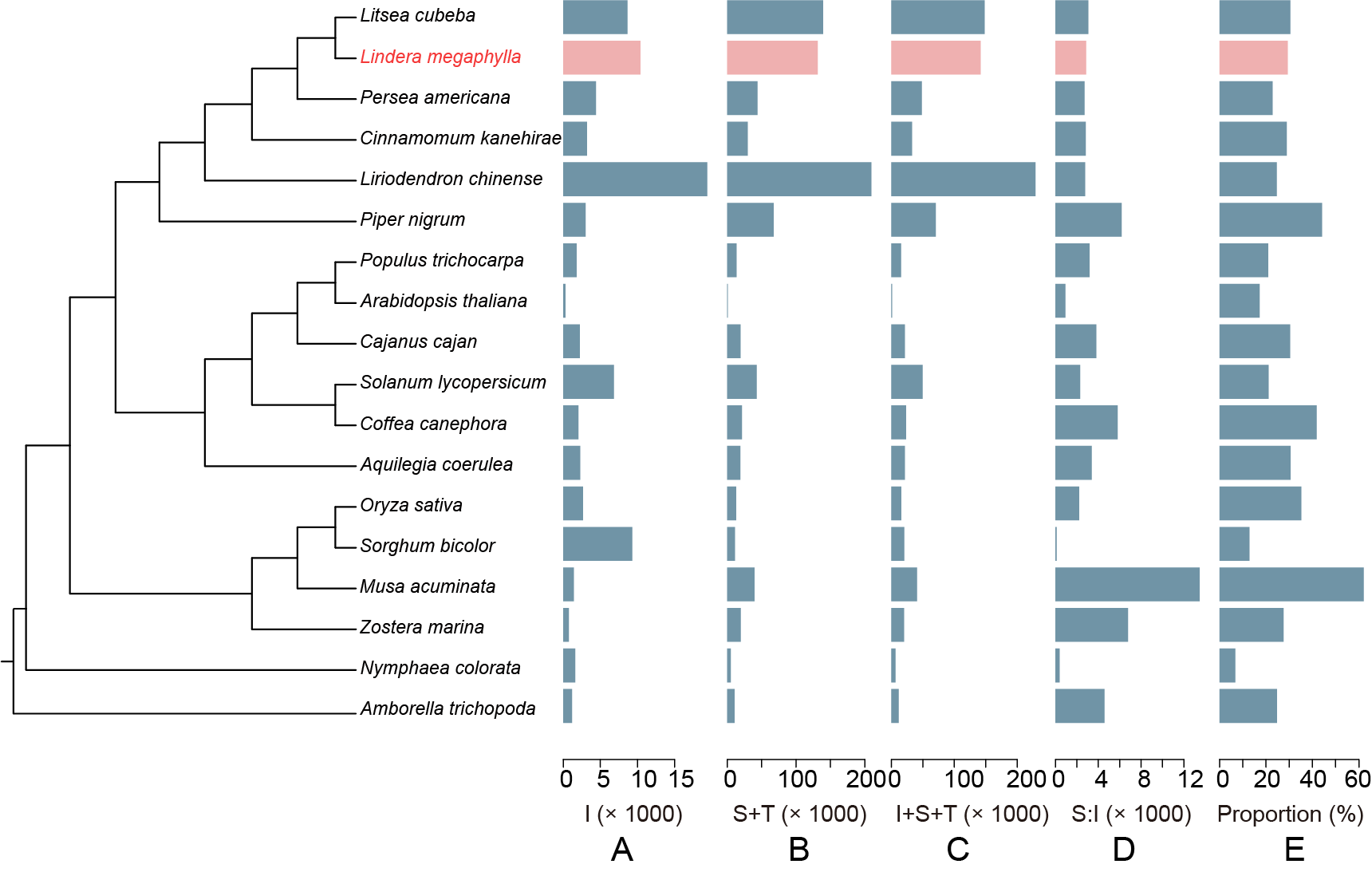


**Figure S7.** **Birth and death of LTR-RTs (long terminal repeat-retrotransposons) in the *L. megaphylla* genome compared to 17 other species. (A)** total numbers of intact LTR-RTs in the genome; **(B)** comparison of *S*+*T* values among these 18 plant species; **(C)** total numbers of intact LTR-RTs and traces of LTR-RT death; **(D)** ratios of solo-LTR to intact LTR-RT (S:I). **(E)** proportions of LTR-RTs found in the clusters with high removal rates (filtered S:I ≥ 3). S, number of solo-LTRs; T, number of truncated LTR-RTs I, number of intact LTR-RTs.


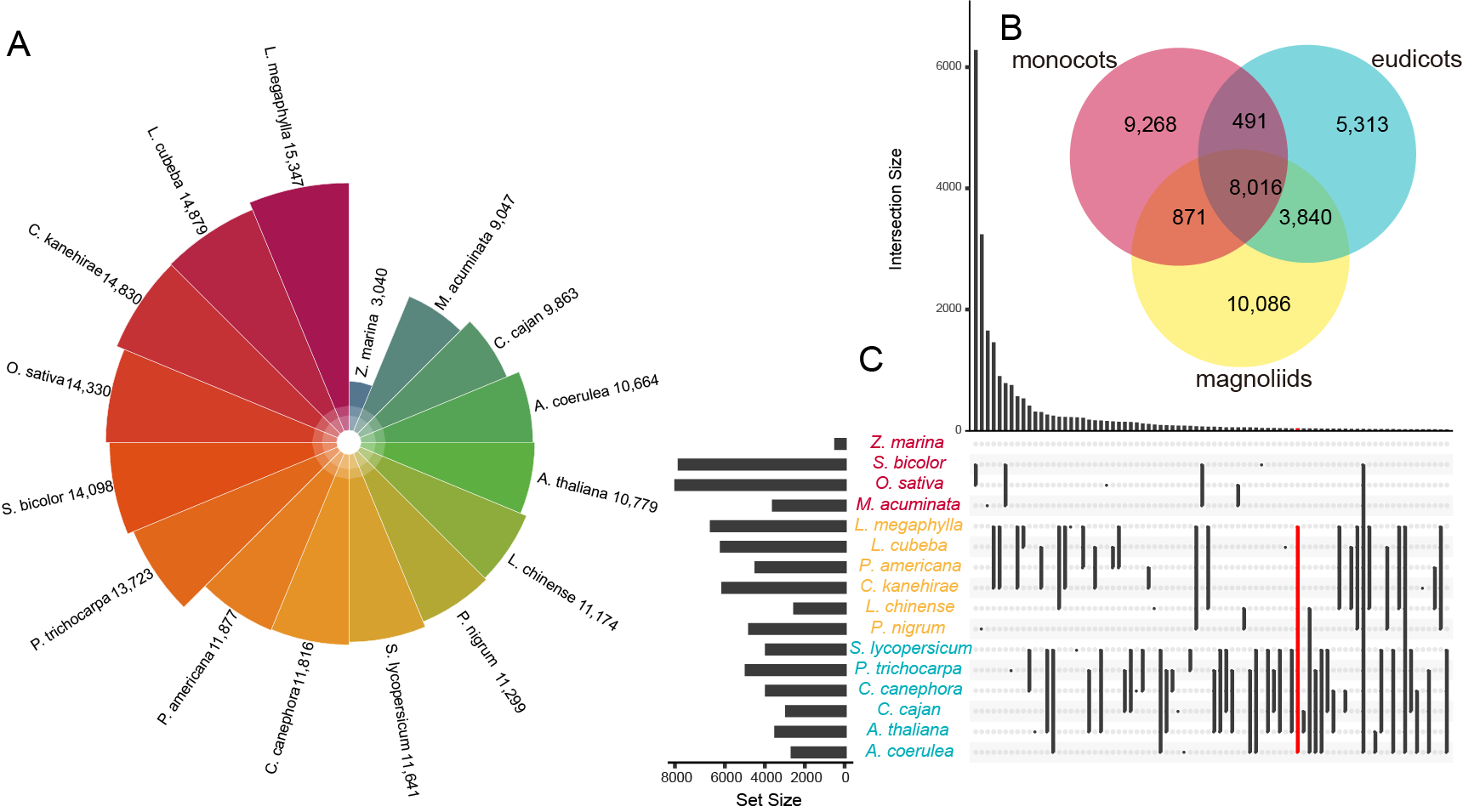


**Figure S8. The gene microsyntenic clusters statistics of 16 species.** **(A)** The polar diagram of clusters in 16 species. **(B)** The Venn diagram represents the synteny clusters of 6 species in magnoliids, 4 species in monocots and 6 species in eudicots. **(C)** The upset plot shows the status of clusters of all 16 species of magnollids, eudicots and monocots. The red line represents the cluster shared among six magnoliids and six eudicots.


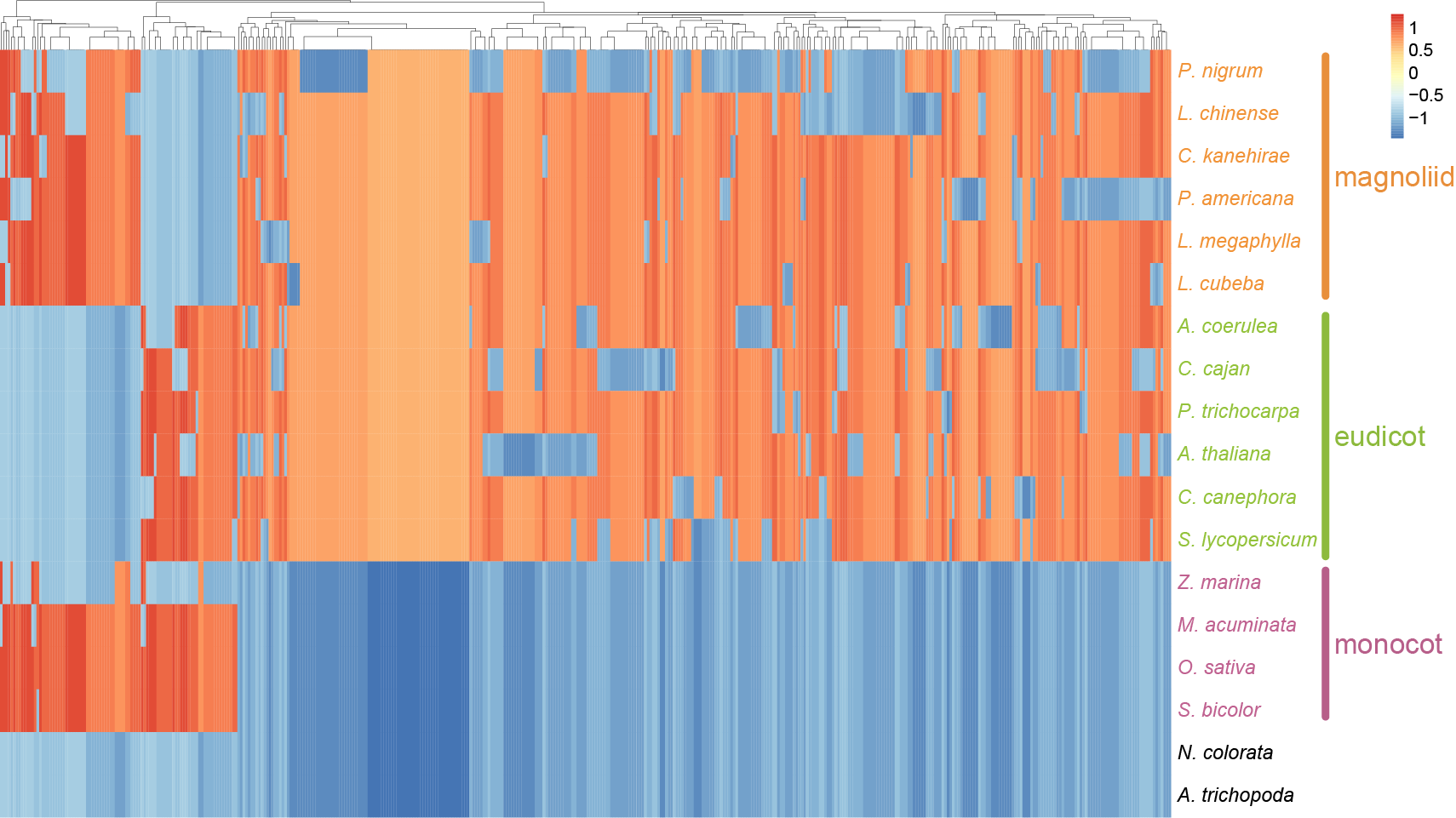


**Figure S9.** **Heatmap of the common clusters of magnoliids-eudicots, magnoliids-monocots and monocots-eudicots.** Low to high values is indicated by a change in color from blue to red.


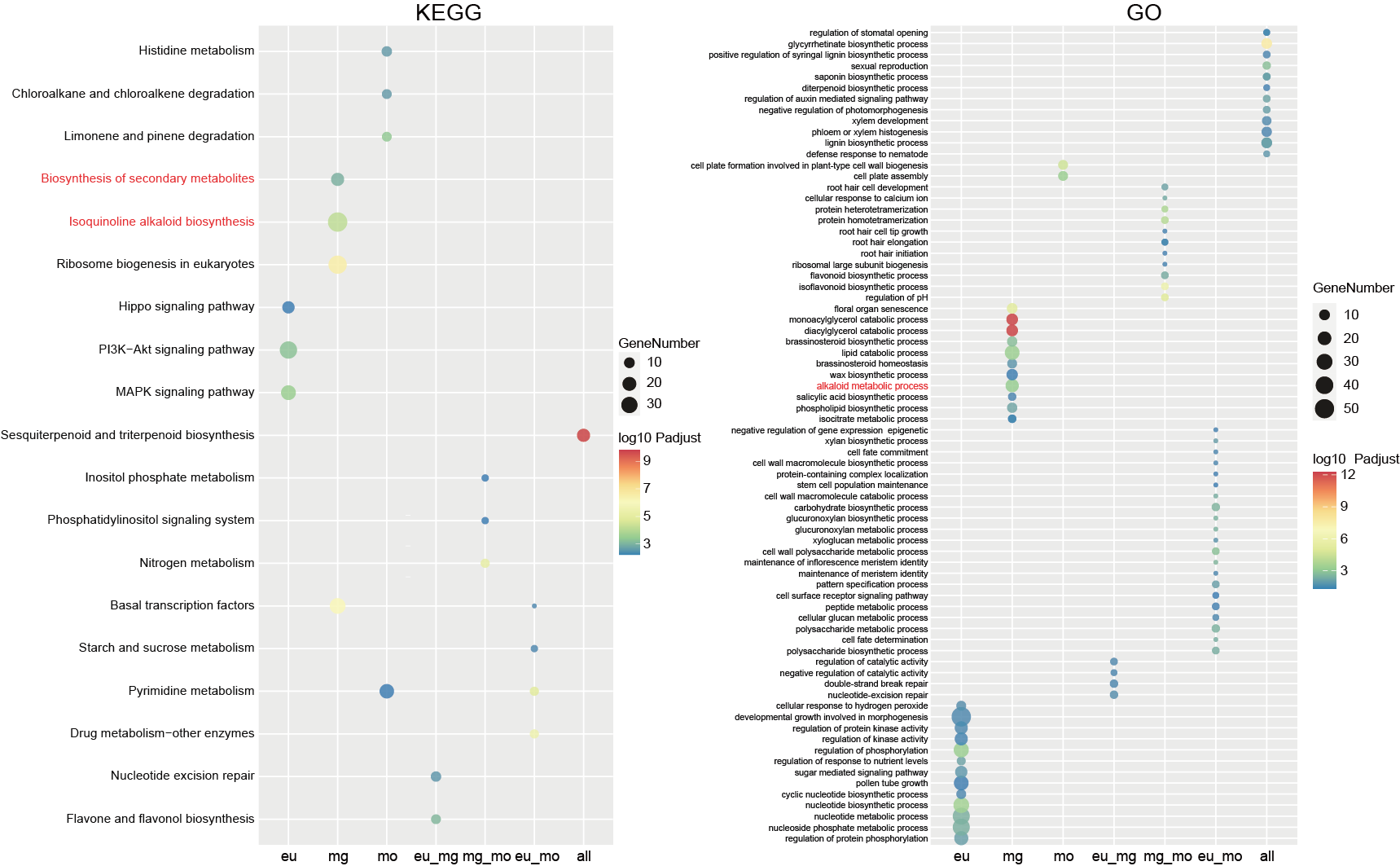


**Figure S10.** **Functional enrichment analysis.** KEGG and GO enrichment analysis of specific synteny clusters in eudicots, magnoliids and monocots, the common clusters shared by eudicots - magnoliids, magnoliids - monocots, eudicots - monocots and magnoliids - eudicots - monocots. GO terms were kept with the adjusted q-values < 0.05.


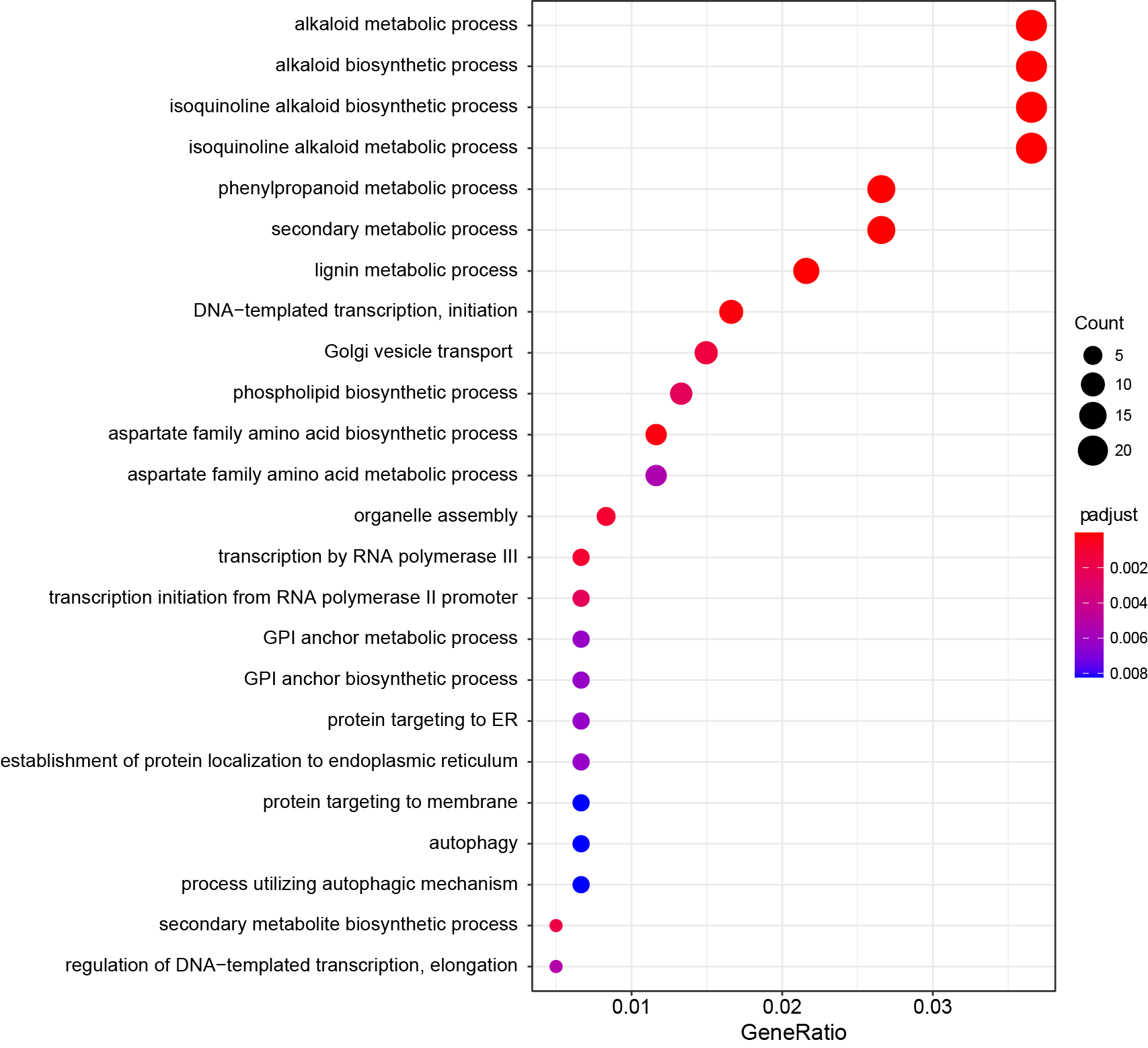


**Figure S11.** **Functional enrichment analysis.** GO enrichment analysis of specific synteny clusters in Lauraceae. GO terms were kept with the adjusted q-values < 0.05.

**
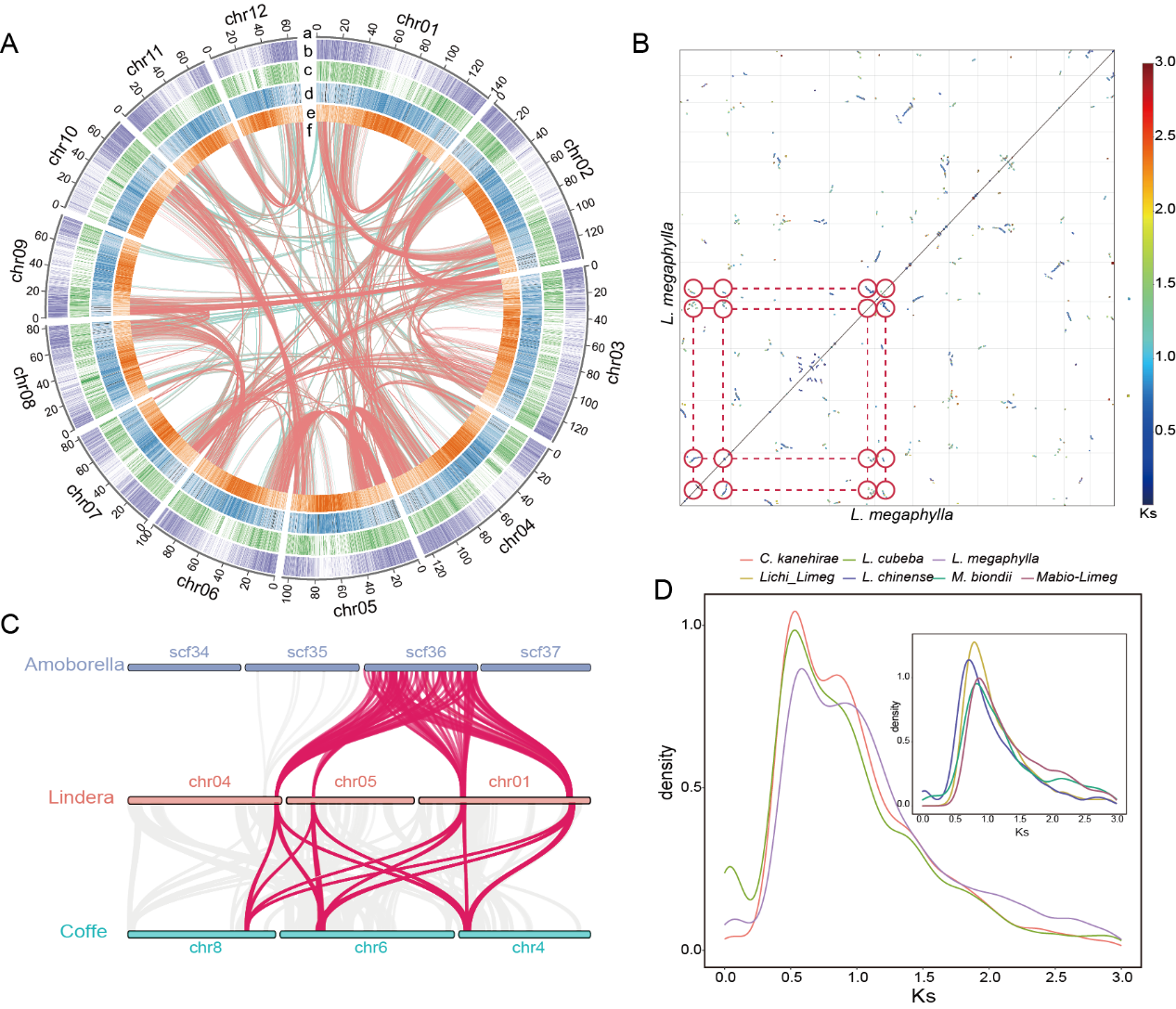
**

**Figure S12.** **(A)** Genomic features of *L. megaphylla*. The features highlighted from innermost to outermost circle are (a) The tracks represent 12 assembled chromosomes. (b-e) The distribution of the Copia density, Gypsy density, LINE1 density and gene density respectively, with densities calculated in 100 Kb windows. (f). The red lines represent syntenic regions for the recent WGD event (57 Mya) and blue lines represent elder WGD event (76 Mya). **(B)** Dot plots show a 4:4 chromosomal relationship within the genome of *L. megaphylla*. The red circles highlight the major whole genome duplication events, the dots represent the synteny gene pairs. **(C)** Synteny patterns between *A. trichopoda*, *L. megaphylla* and *C. canephora*. These patterns show that a typical ancestral region in the basal angiosperm *Amborella* can be tracked to four regions in *L. megaphylla* and a typical ancestral region in *C. canephora* can be tracked to three regions in *L. megaphylla*. These collinear relationships are highlighted by red lines. **(D)** Ks distributions of orthologous (*C. canephora*-*L. megaphylla*) and paralogous genes (*L. megaphylla*, *L. cubeba*, *C. kanehirae* and *L. chinense*).


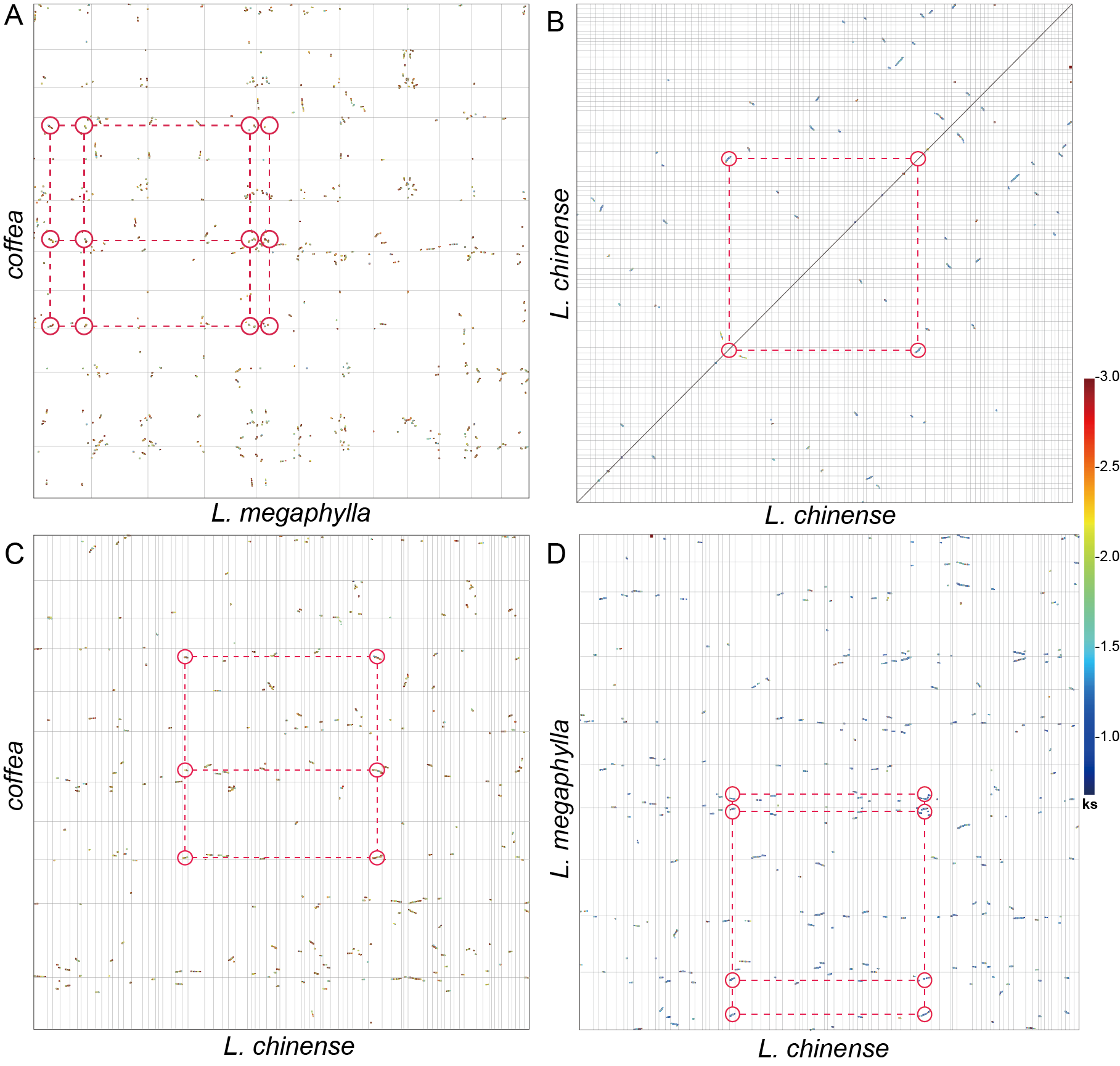


**Figure S13.** **Comparison among *L. megaphylla***, ***C. canephora***, **and *L. chinense* genomes.** **(A)** Dot plots of orthologs between *L. megaphylla* and *C. canephora*, **(B)** paralogues of *L. chinense*, **(C)** orthologs between *L. chinense* and *C. canephora*. **(D)** orthologs between *L. megaphylla* and *L. chinense*.


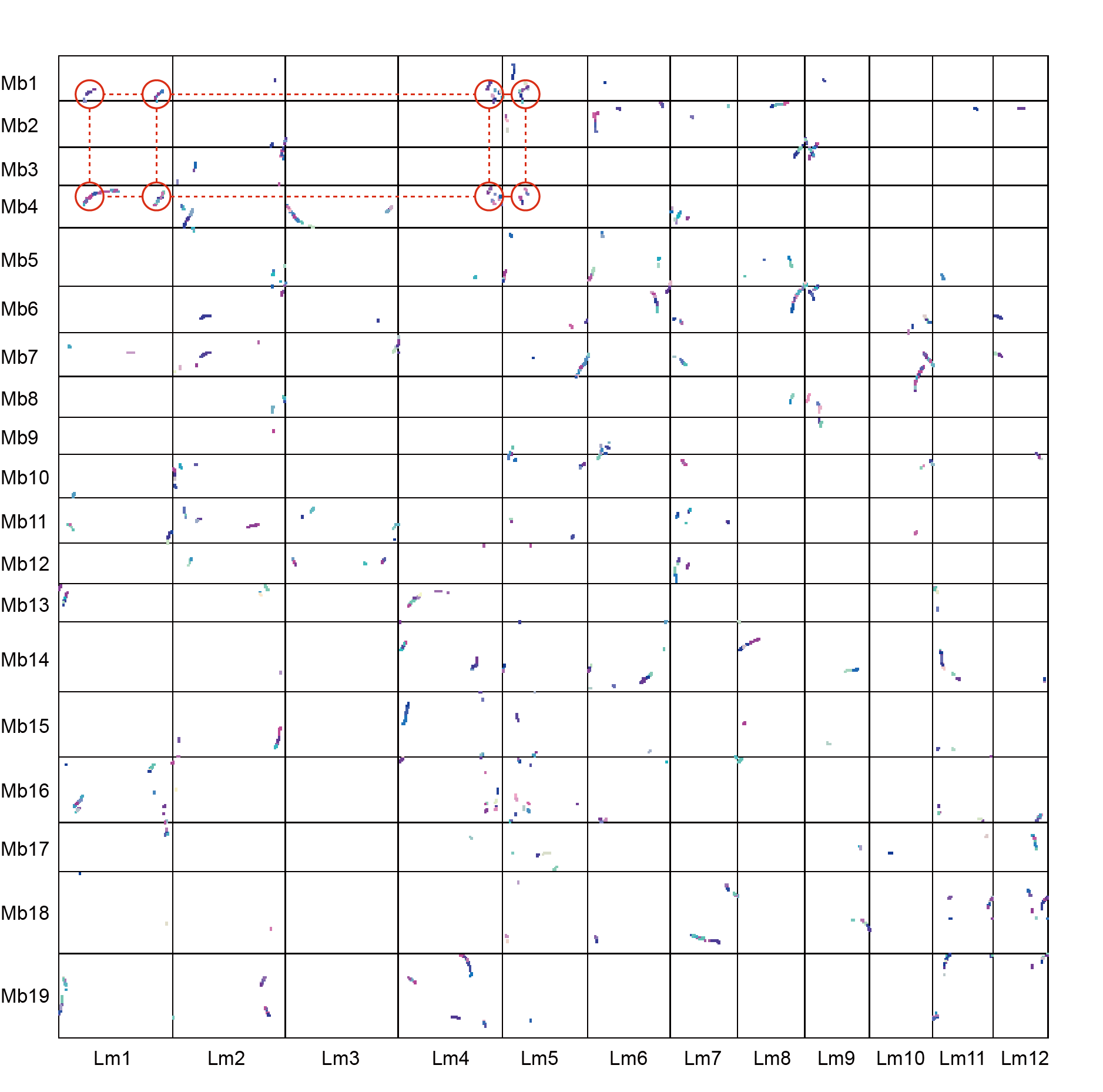


**Figure S14. Dot plots of orthologs between *L. megaphylla* and *M. biondii* genomes.**


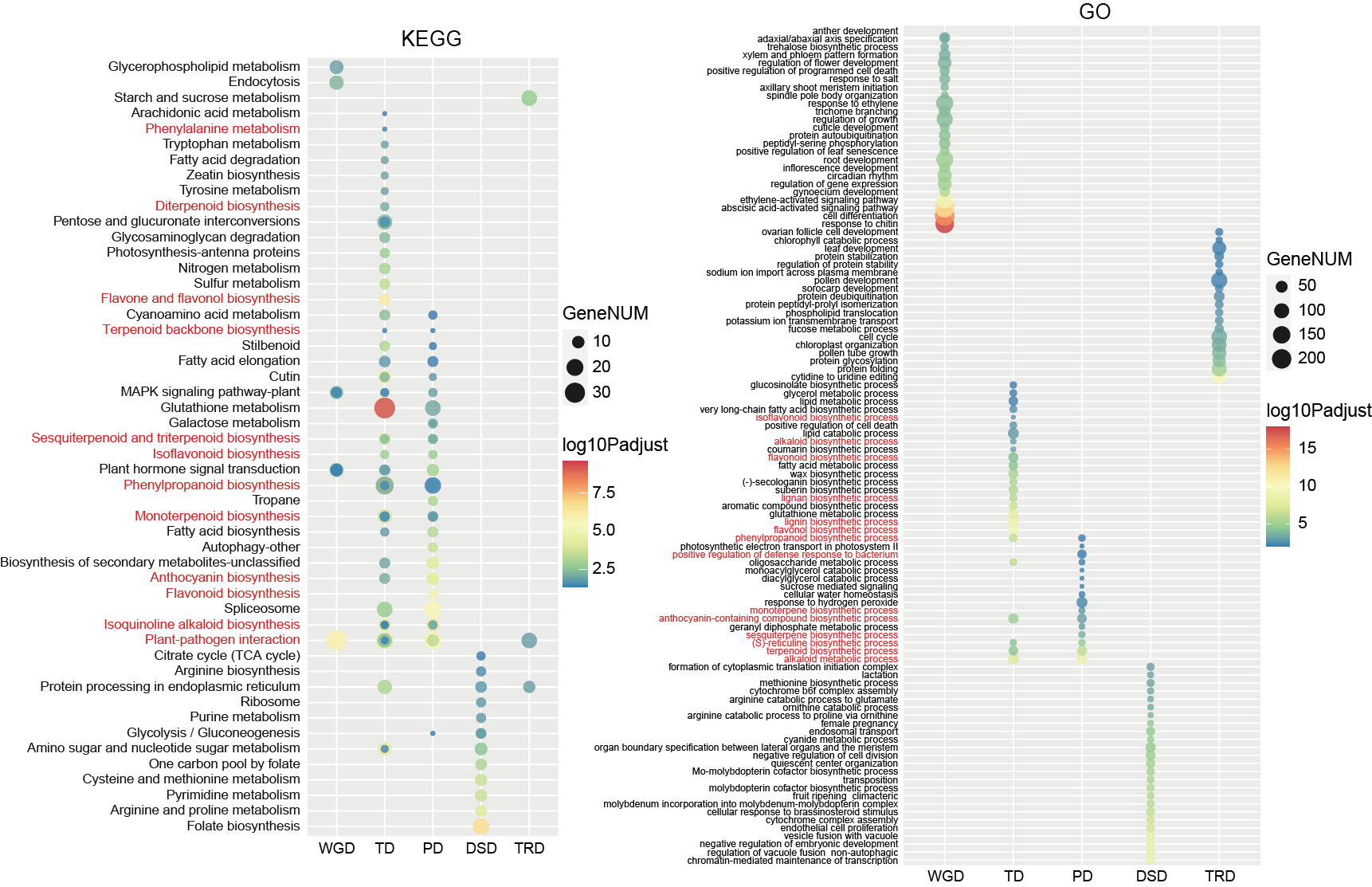


**Figure S15. KEGG and GO enrichment analysis of five duplication patterns in *L. megaphylla*.** The red letter denotes terms relating to the decay-resistance of wood. GO terms were kept with the adjusted q-values < 0.05. Red letters indicate terms related to wood decay resistance.


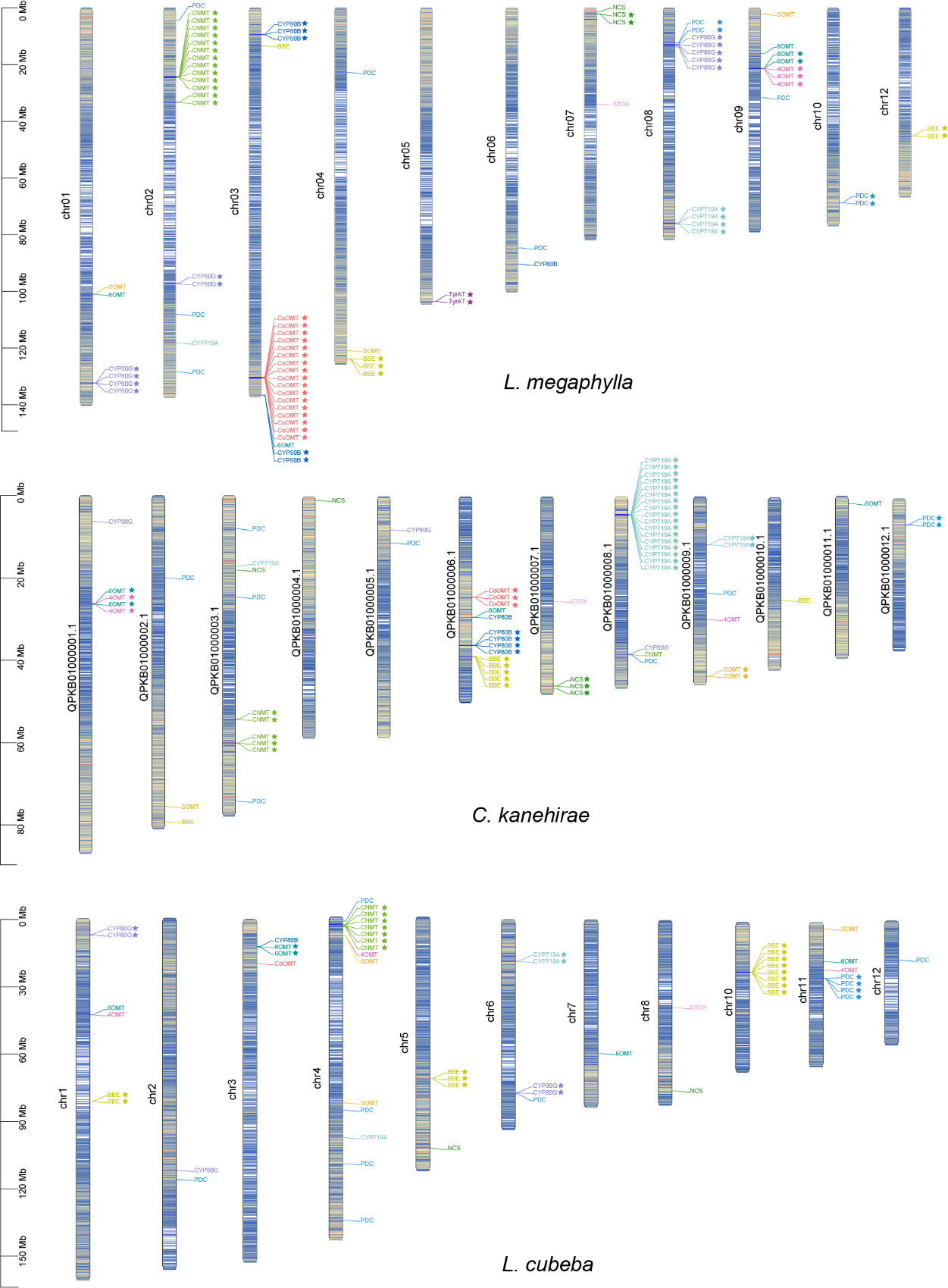


**Figure S16.** Genomic distribution of the TD/PD (tandem or proximal duplications) clusters of the BIA pathway genes (*TyrAT*, *PDC*, *NCS*, *6OMT*, *CNMT*, *CYP80B*, *4OMT*, *CYP80G*, *RNMT*, *BBE*, *SOMT*, *CYP719A*, *STOX* and *CoOMT*) in *L. megaphylla*, *C. kanehirae* and *L. cubeba*. The asterisk indicates the TD/PD cluster genes.


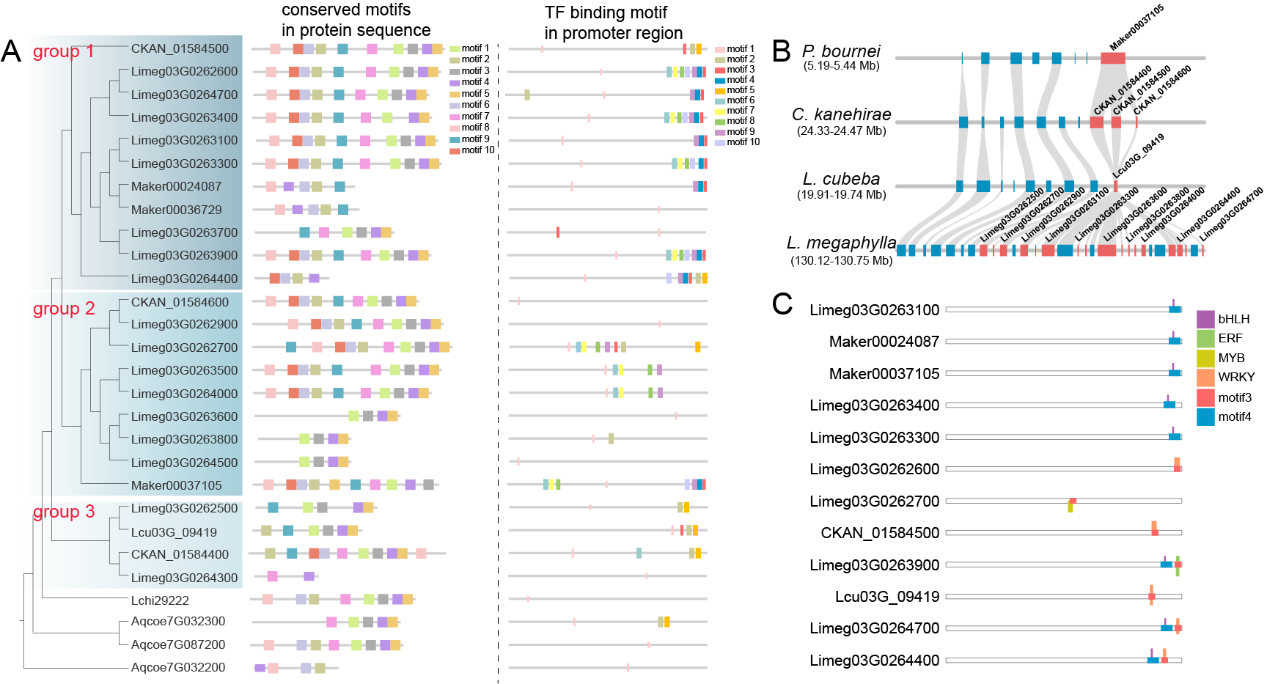


**Figure S17. Characterization of *CoOMT* genes in Lauraceae.** **(A)** Different panels represent the phylogenetic tree of the *CoOMT* gene family (the left panel), the distribution of motifs in protein sequence (the middle), the distribution of motifs in the promoter sequence (the right). **(B)** The syntenic block containing the *CoOMT* genes in *L. chinense*, *P. bournei*, *C. kanehirae*, *L. cubeba* and *L. megaphylla*. Red squares represent the *CoOMT* genes and blue ones represent other genes on the syntenic block. **(C)** The conserved motifs and predicated transcription factor binding sites (TFBSs) on promoter motifs uniquely found among the Lauraceae species. Fat squares represent the motifs and thin ones the TFBSs.


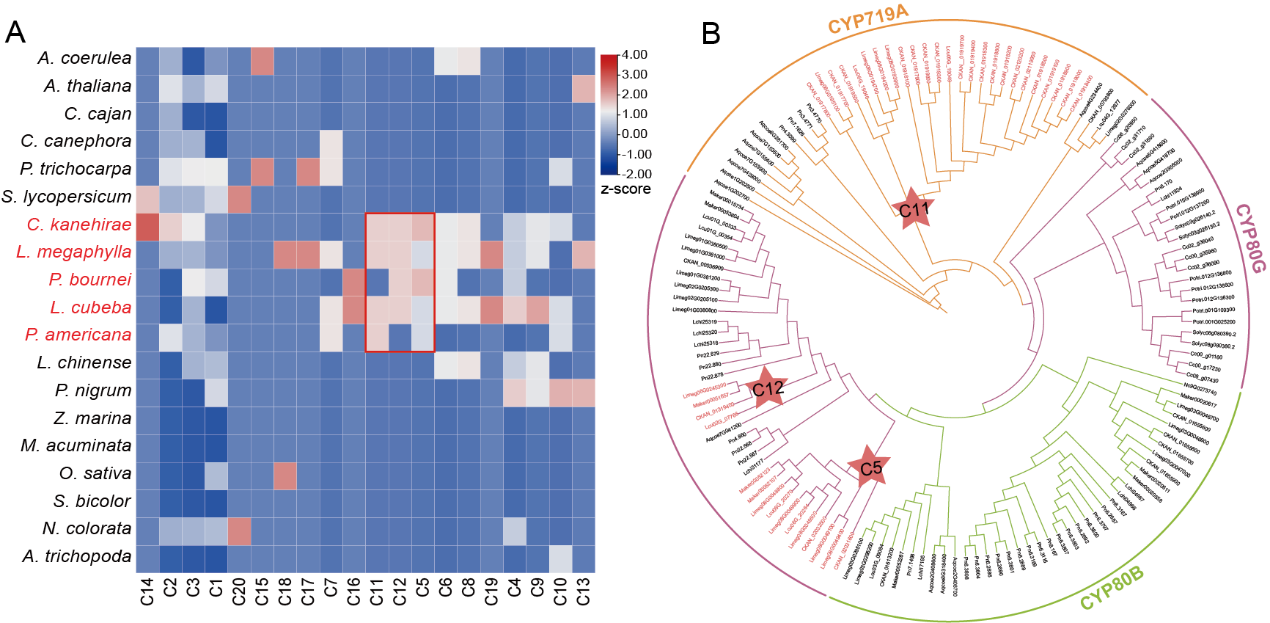


**Figure S18. *CYP* gene families. (A)** Heatmap of 20 microsynteny clusters identified to be related with *CYP* gene families (here *CYP80B*, *CYP80G*, and *CYP719A*), three of which specific to Lauraceae were highlighted in a red square. Color in the heatmap was determined by the gene number found in each cluster each species. **(B)** Phylogenetic analysis of *CYP* gene families. The gene names of Lauraceae species are shown in red, and the red stars represent genes inside the Lauraceae-specific gene clusters identified in Figure S18A.


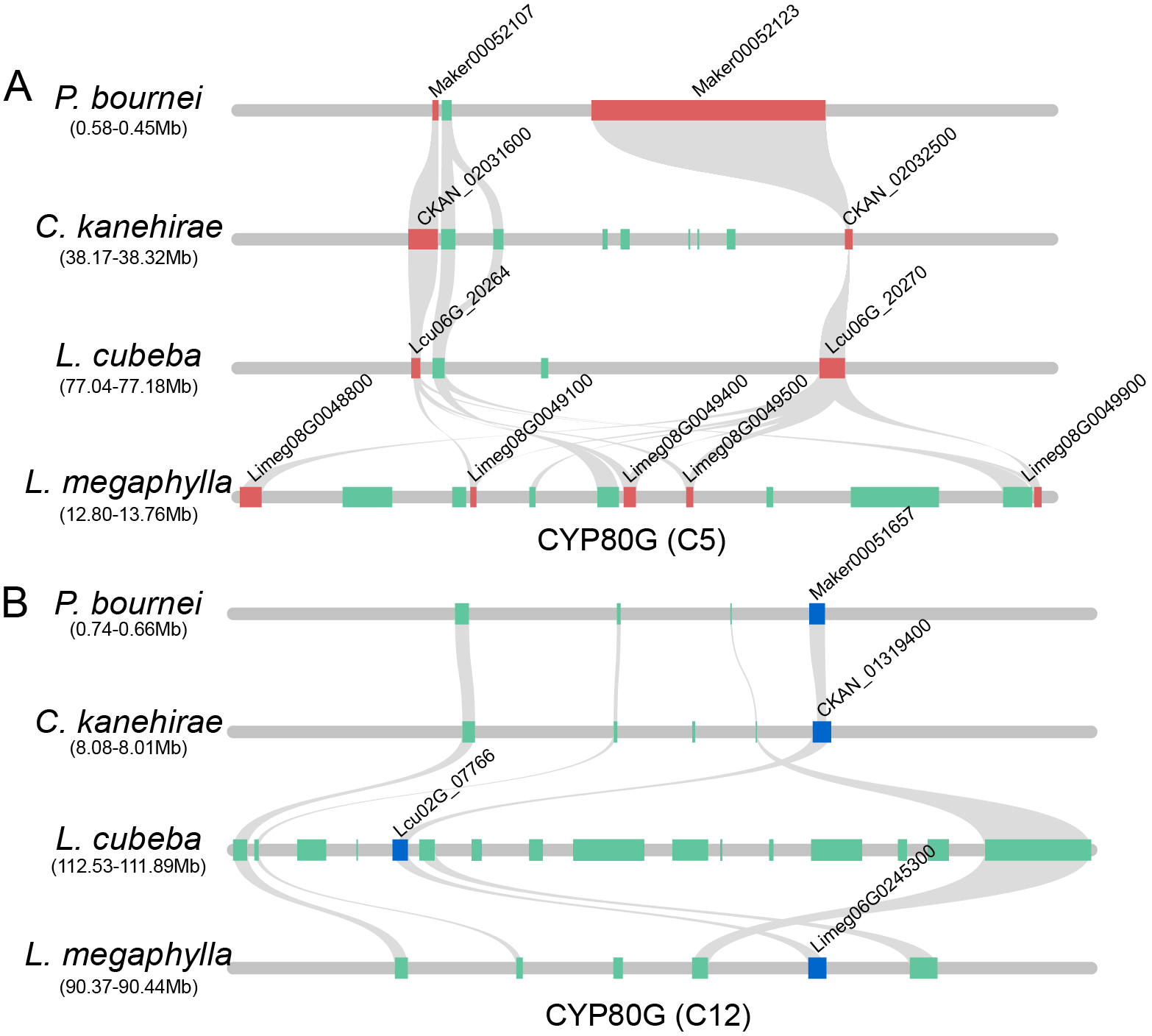


**Figure S19. Synteny blocks of *CYP80G* genes in Lauraceae.** The syntenic block containing of *CYP80G* gene family inside the Lauraceae-specific microsynteny gene cluster, C5 (**Figure S19A**) and C12 (**Figure S19B**) genes, and these two clusters were identified in Figure S18A and 18B. Here this syntenic block was compared among *P. bournei*, *C. kanehirae*, *L. cubeba* and *L. megaphylla*. Red represents the *CYP80G* genes and aquamarine represents other genes on the syntenic block.


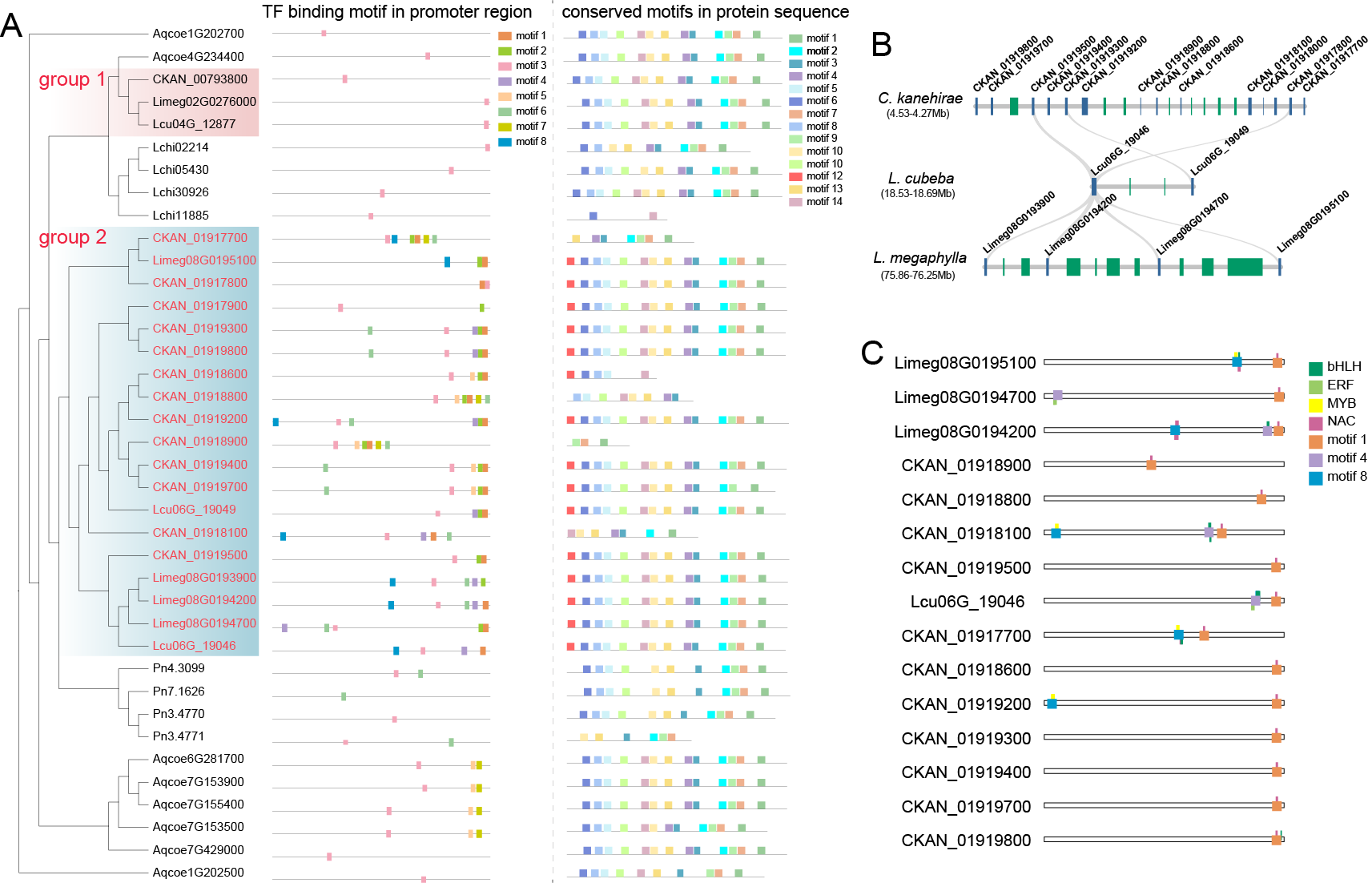


**Figure S20. Characterization of *CYP719A* genes in Lauraceae.** **(A)** Different panels represent the phylogenetic tree of the *CYP719A* gene family (the left), the distribution of motifs in the promoter sequence (the middle) and the distribution of motifs in protein sequence (the right). Dashed boxes highlight the gene and promoter motifs uniquely found among the Lauraceae species. **(B)** The syntenic block containing of *CYP719A* gene family inside the Lauraceae-specific microsynteny gene cluster, C11 genes, and this cluster was identified in Figure S18A and 18B. Here this syntenic block was compared among *P. bournei*, *C. kanehirae*, *L. cubeba* and *L. megaphylla*. Blue squares represent the *CYP719A* genes and green ones represent other genes on the syntenic block. **(C)** The conserved motifs and predicated transcription factor binding sites (TFBSs) on promoter motifs uniquely found among the Lauraceae species. Fat squares represent the motifs and thin ones the TFBSs.

**
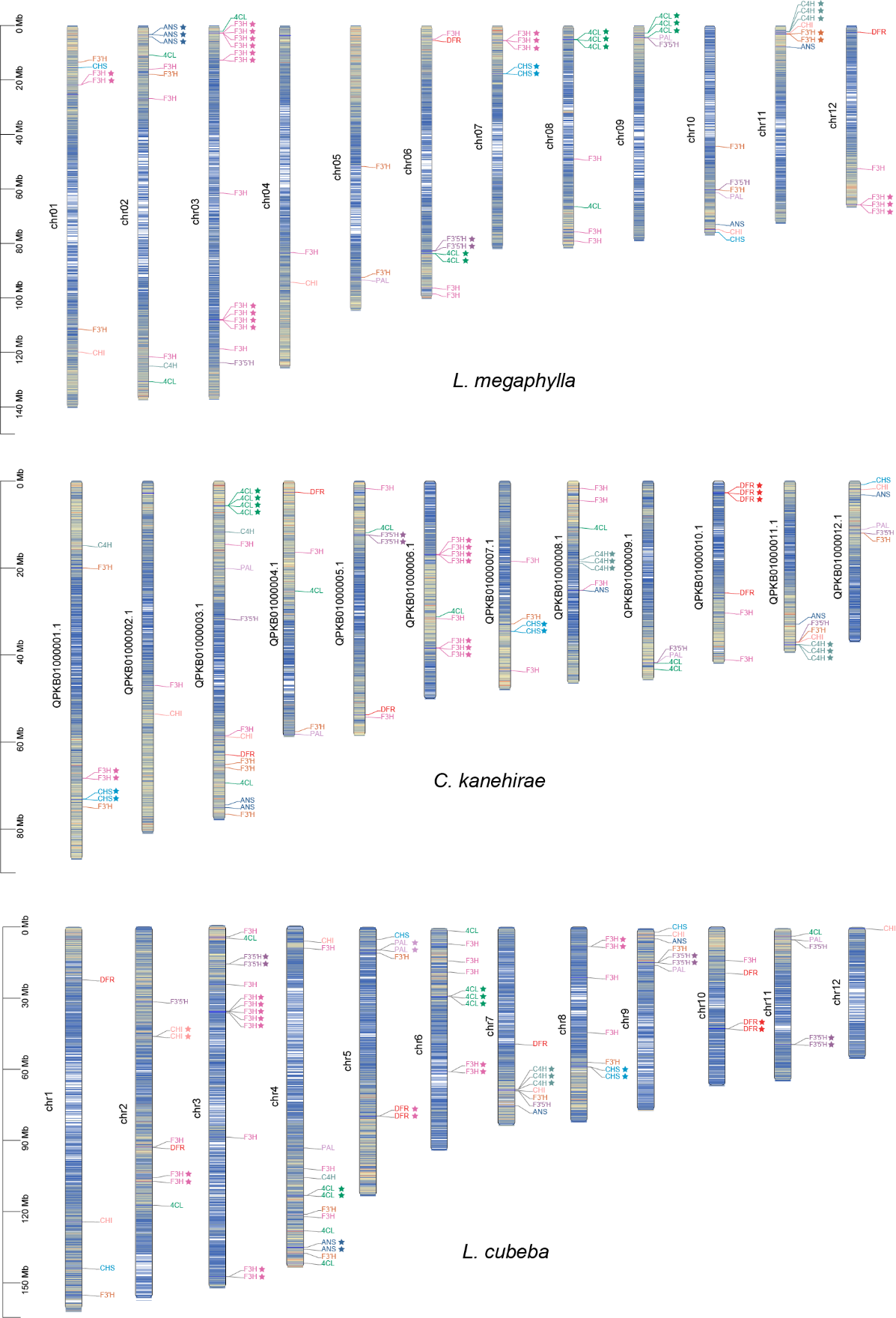
**

**Figure S21.** Genomic distribution of the TD/PD (tandem or proximal duplications) clusters of the general phenylpropanoid (*PAL*, *C4H* and *4CL*) and flavonoid pathway genes (*CHS*, *CHI*, *F3H*, *FLS*, *F3'H*, *F3'5'H*, *DFR* and *ANS*) in *L. megaphylla*, *C. kanehirae* and *L. cubeba*. The asterisk indicates the TD/PD cluster genes.

**
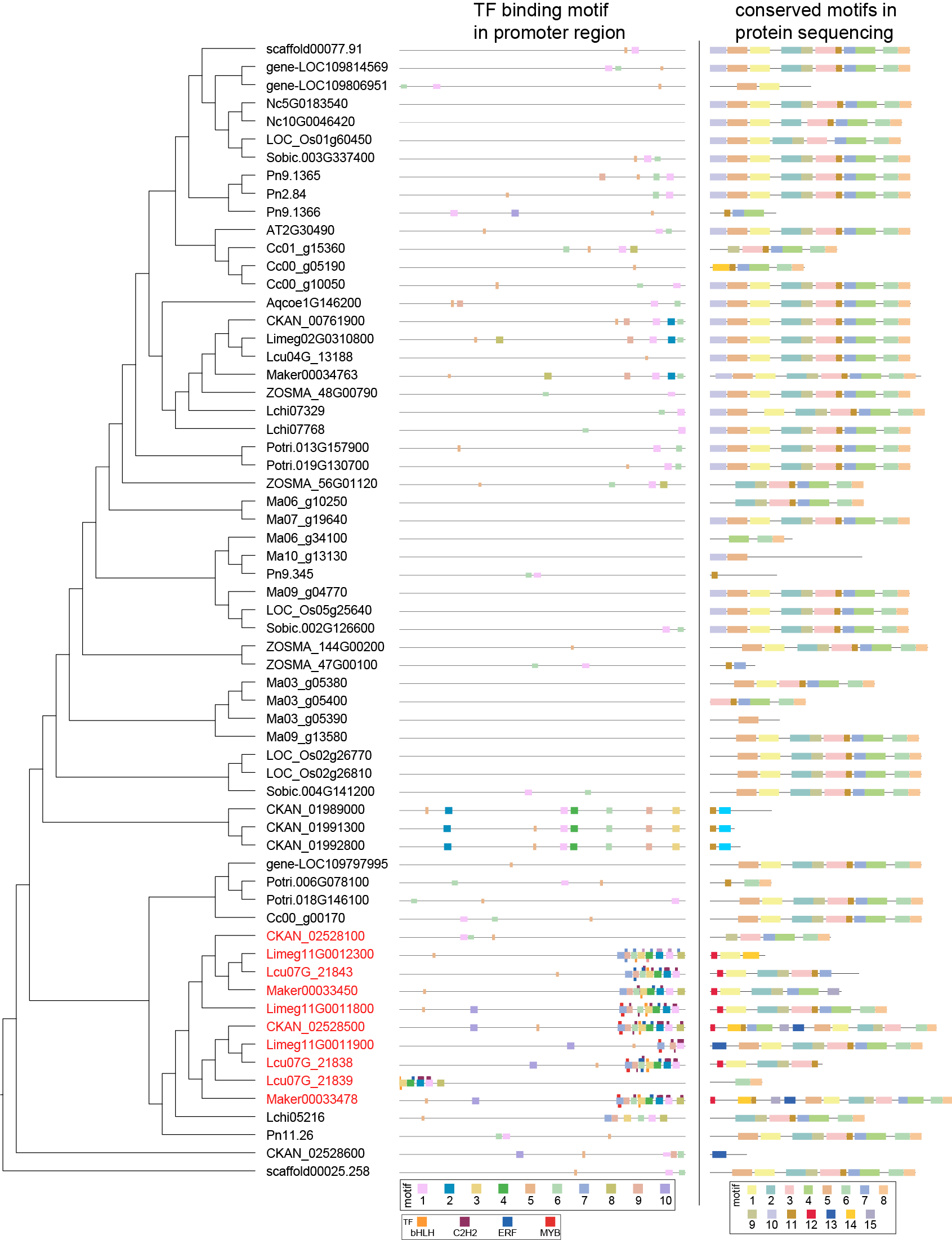
**

**Figure S22.** **Characterization of *C4H* genes in Lauraceae.** Different panels represent the phylogenetic tree of the *C4H* gene family (the left), the distribution of motifs in the promoter sequence and the predicated transcription factor binding sites (TFBS) (the middle) and the distribution of motifs in protein sequence (the right), Fat squares represent the motifs and thin ones the TFBSs.


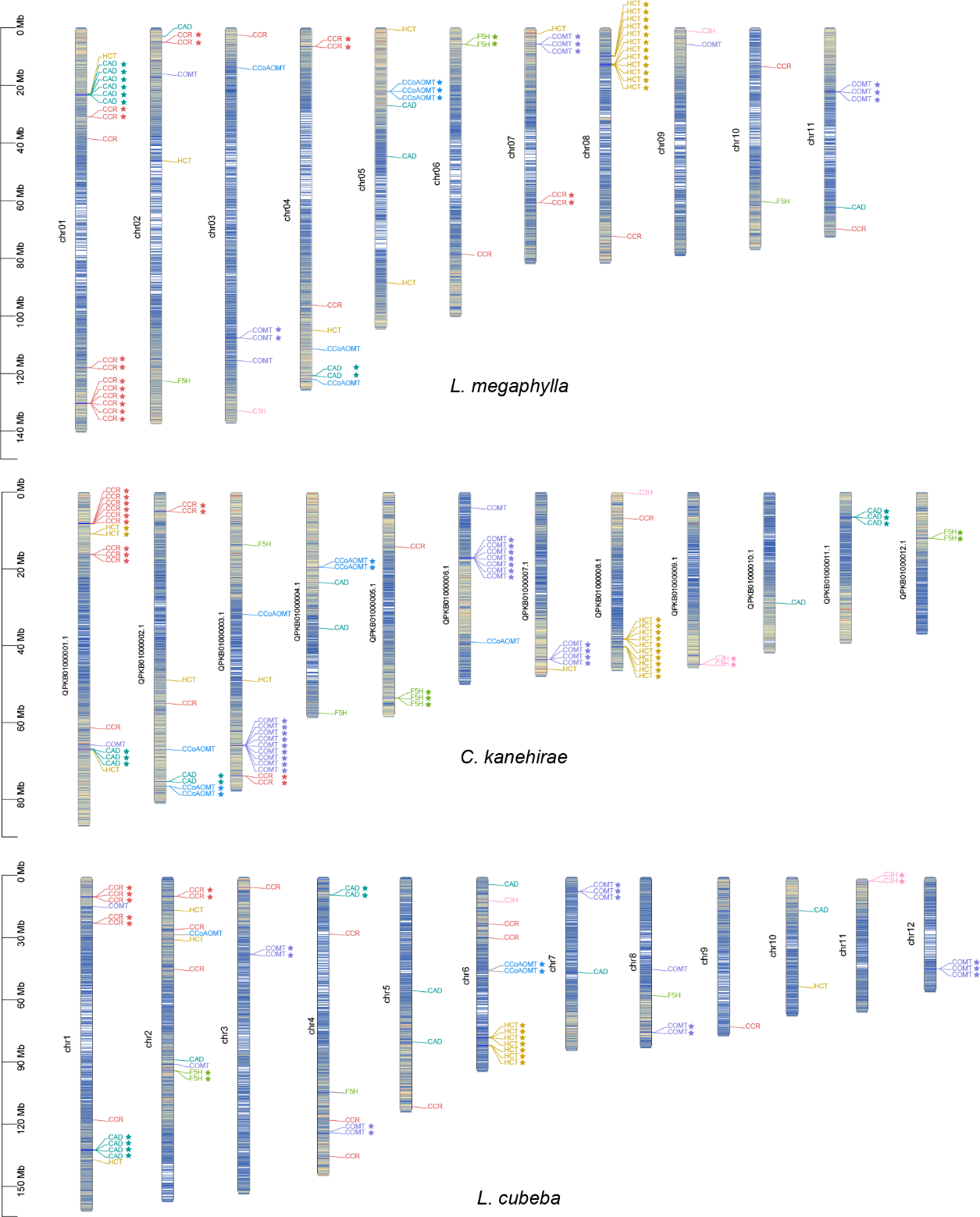


**Figure S23.** Genomic distribution of the TD/PD (tandem or proximal duplications) clusters of the lignin pathway genes (*C3H*, *HCT*, *CCoAOMT*, *CCR*, *F5H*, *COMT* and *CAD*) in *L. megaphylla*, *C. kanehirae* and *L. cubeba*. The asterisk indicates the TD/PD cluster genes.


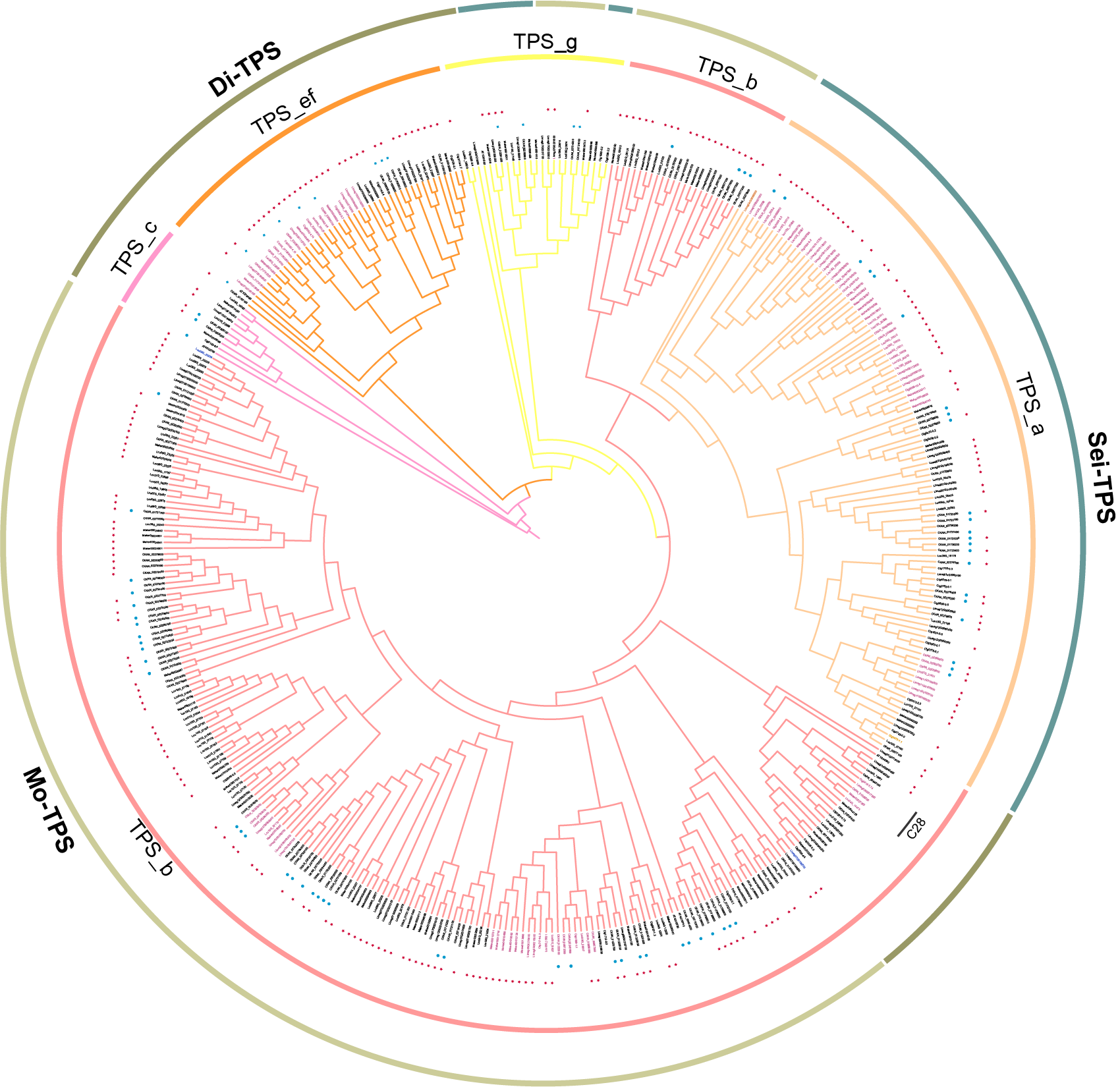


**Figure S24.** **Phylogenetic analysis of *TPS* gene family.** Blue dots indicate *TPS* genes that have been reported in the genomes of *C. kanehirae* and *A. thaliana*. Red stars showed the tandem and proximal duplication (TD/PD) genes.


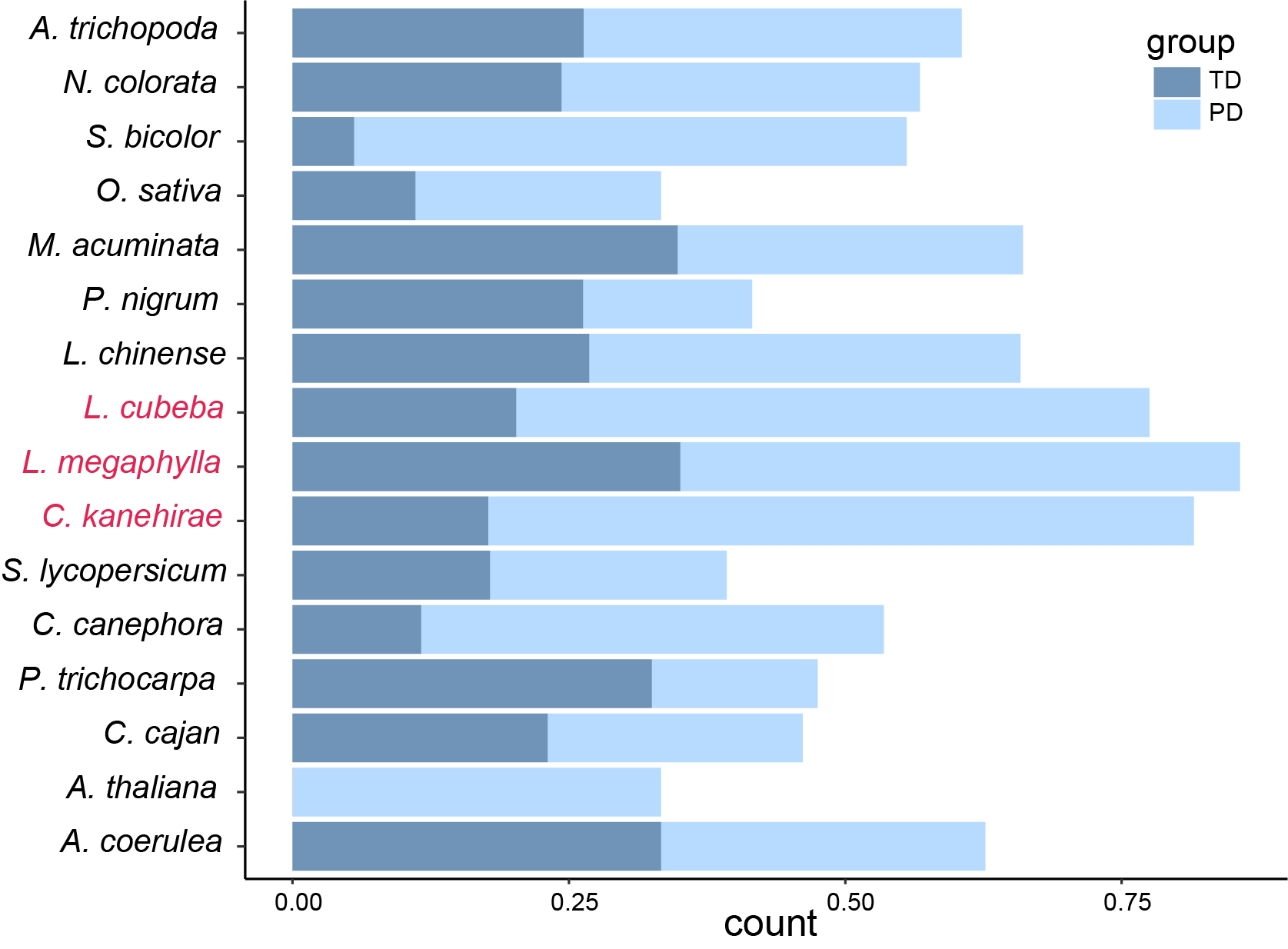


**Figure S25.** Proportion of the contribution of tandem and proximal duplications (TD/PD) in *TPS* gene famly in each species. Red represents the Lauraceae species.


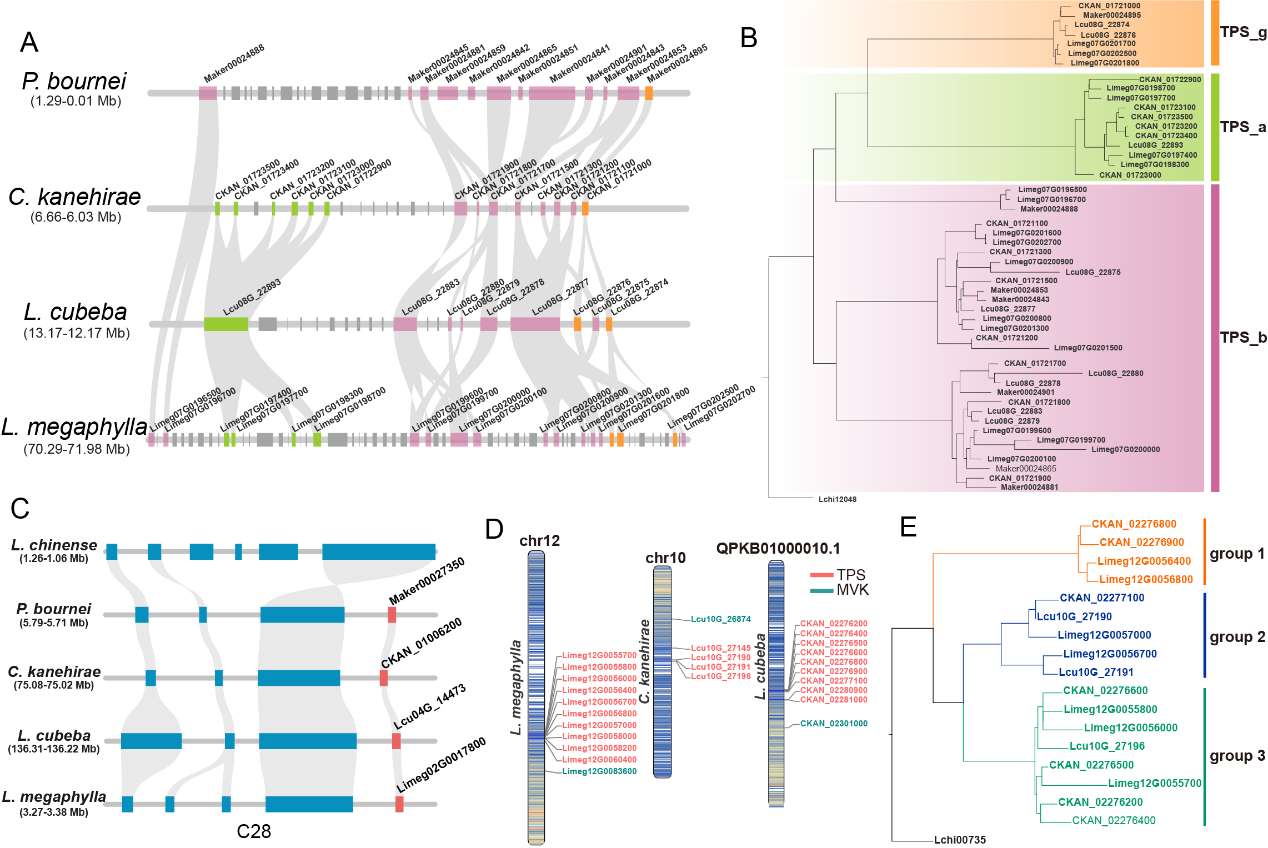


**Figure S26.** **Characteristics of terpene synthase (*TPS*) genes in Lauraceae. (A)** Syntenic relationships of the tandem/proximal cluster of *TPS* genes in four Lauraceae species. **(B)** Phylogenetic analysis of the genes in Figure S26A. **(C)** The syntenic block containing *TPS* gene family members within the Lauraceae-specific microsynteny gene cluster (C28). This syntenic block was compared among *L. chinense*, *P. bournei*, *C. kanehirae*, *L. cubeba*, and *L. megaphylla*. Red squares represent *TPS* genes and blue squares represent other genes in the syntenic block. **(D)** Chromosomal distribution of terpenoid biosynthesis gene clusters in three Lauraceae species. MVK: mevalonate kinase. **e:** Phylogenetic analysis of terpenoid biosynthesis genes in Figure S26D.

| **Library_stratey** | **accession** | **study** | **bioproject_accession** | **biosample_accession** | **Platform** | **Raw Reads (Million)** | **Raw Bases (Gb)** | **Clean Reads (Million)** | **Clean Bases (Gb)** |
| --- | --- | --- | --- | --- | --- | --- | --- | --- | --- |
| **WGS** | SRR19759931 | SRP382804 | PRJNA850554 | SAMN29213659 | ONT PromethION | 10.336 | 178.78 | NA | NA |
| **WGS** | SRR19759930 | SRP382804 | PRJNA850554 | SAMN29213660 | ILLUMINA | 356.038 | 53.41 | 351.001 (98.59%) | 52.540 (98.38%) |
| **WGS** | SRR19759919 | SRP382804 | PRJNA850554 | SAMN29213661 | ILLUMINA | 712.464 | 106.87 | 704.940 (98.94%) | 105.541 (98.76%) |
| **Hi-C** | SRR19759913 | SRP382804 | PRJNA850554 | SAMN29213659 | ILLUMINA | 1,488.194 | 223.23 | NA | NA |
| **RNA-Seq** | SRR19759912 | SRP382804 | PRJNA850554 | SAMN29213662 | ILLUMINA | 47.352 | 7.10 | 45.771 (96.66%) | 6.823 (96.06%) |
| **RNA-Seq** | SRR19759911 | SRP382804 | PRJNA850554 | SAMN29213663 | ILLUMINA | 47.877 | 7.18 | 46.732 (97.61%) | 6.971 (97.07%) |
| **RNA-Seq** | SRR19759910 | SRP382804 | PRJNA850554 | SAMN29213664 | ILLUMINA | 36.295 | 5.44 | 35.436 (97.63%) | 5.281 (97.01%) |
| **RNA-Seq** | SRR19759909 | SRP382804 | PRJNA850554 | SAMN29213665 | ILLUMINA | 46.785 | 7.02 | 46.479 (99.35%) | 6.924 (98.66%) |
| **RNA-Seq** | SRR19759908 | SRP382804 | PRJNA850554 | SAMN29213666 | ILLUMINA | 49.446 | 7.42 | 49.150 (99.40%) | 7.338 (98.93%) |
| **RNA-Seq** | SRR19759907 | SRP382804 | PRJNA850554 | SAMN29213667 | ILLUMINA | 46.445 | 6.97 | 46.140 (99.34%) | 6.889 (98.88%) |
| **RNA-Seq** | SRR19759929 | SRP382804 | PRJNA850554 | SAMN29213668 | ILLUMINA | 51.740 | 7.76 | 51.427 (99.40%) | 7.681 (98.97%) |
| **RNA-Seq** | SRR19759928 | SRP382804 | PRJNA850554 | SAMN29213669 | ILLUMINA | 49.739 | 7.46 | 49.440 (99.40%) | 7.380 (98.91%) |
| **RNA-Seq** | SRR19759927 | SRP382804 | PRJNA850554 | SAMN29213670 | ILLUMINA | 39.469 | 5.92 | 39.269 (99.49%) | 5.864 (99.06%) |
| **RNA-Seq** | SRR19759926 | SRP382804 | PRJNA850554 | SAMN29213671 | ILLUMINA | 36.696 | 5.50 | 35.662 (97.18%) | 5.317 (96.59%) |
| **RNA-Seq** | SRR19759925 | SRP382804 | PRJNA850554 | SAMN29213672 | ILLUMINA | 40.801 | 6.12 | 39.746 (97.41%) | 5.926 (96.83%) |
| **RNA-Seq** | SRR19759924 | SRP382804 | PRJNA850554 | SAMN29213673 | ILLUMINA | 36.913 | 5.54 | 35.937 (97.36%) | 5.358 (96.77%) |
| **RNA-Seq** | SRR19759923 | SRP382804 | PRJNA850554 | SAMN29213674 | ILLUMINA | 47.982 | 7.20 | 47.754 (99.52%) | 7.130 (99.07%) |
| **RNA-Seq** | SRR19759922 | SRP382804 | PRJNA850554 | SAMN29213675 | ILLUMINA | 54.446 | 8.17 | 54.094 (99.35%) | 8.054 (98.61%) |
| **RNA-Seq** | SRR19759921 | SRP382804 | PRJNA850554 | SAMN29213676 | ILLUMINA | 51.046 | 7.66 | 50.708 (99.34%) | 7.559 (98.73%) |
| **RNA-Seq** | SRR19759920 | SRP382804 | PRJNA850554 | SAMN29213677 | ILLUMINA | 56.772 | 8.52 | 56.512 (99.54%) | 8.420 (98.88%) |
| **RNA-Seq** | SRR19759918 | SRP382804 | PRJNA850554 | SAMN29213678 | ILLUMINA | 49.114 | 7.37 | 48.911 (99.59%) | 7.303 (99.13%) |
| **RNA-Seq** | SRR19759917 | SRP382804 | PRJNA850554 | SAMN29213679 | ILLUMINA | 54.897 | 8.24 | 54.617 (99.49%) | 8.159 (99.08%) |
| **RNA-Seq** | SRR19759916 | SRP382804 | PRJNA850554 | SAMN29213680 | ILLUMINA | 39.240 | 5.89 | 38.290 (97.58%) | 5.713 (97.06%) |
| **RNA-Seq** | SRR19759915 | SRP382804 | PRJNA850554 | SAMN29213681 | ILLUMINA | 40.317 | 6.05 | 39.404 (97.74%) | 5.876 (97.16%) |
| **RNA-Seq** | SRR19759914 | SRP382804 | PRJNA850554 | SAMN29213682 | ILLUMINA | 42.648 | 6.40 | 41.373 (97.01%) | 6.173 (96.50%) |

**Table S1**. Summary of Nanopore and Illumina sequencing data (Hi-C and RNA sequencing) generated in the present study.

| **Versions of assembly** | **Strategy** | **Assemblied genome size (Gb)** | **Sequence number** | **N50 (Mbp)** | **Max length (Mbp)** | **Gene completeness (%)** |
| --- | --- | --- | --- | --- | --- | --- |
| **v0.1** | SMARTDENOVO | 1.44 | 4635 | 0.675 | 4.3 | NA |
| **v0.2** | WTDBG | 1.30 | 26337 | 0.112 | 2.4 | NA |
| **v0.3** | Corrected by CANU + SMARTDENOVO (-k 23) | 1.28 | 2169 | 1.37 | 11.9 | NA |
| **v0.4** | Corrected by CANU + WTDBG (-p 19) | 1.25 | 10389 | 0.629 | 6.3 | NA |
| **v1.0** | V0.3 + pilon×3 | 1.31 | 2169 | 1.39 | 12.1 | 90.80% |
| **v1.1** | V1.0 + Hi-C + gapclose + pilon×4 | 1.27 | 1407 | 2.6 | 13.5 | 90.70% |

**Table S2.** Statistics of the different versions of *L. megaphylla* genome assembly.

| **Type** | **Number** | **Length (bp)** | **Percent** |
| --- | --- | --- | --- |
| **chromsome-scale scaffold** | 12 | 481,946,564 | 95.10% |
| **mitochondrial** | 1 | 1,110,254 | 0.09% |
| **chloroplast** | 1 | 152,763 | 0.01% |
| **contig-scale scaffold** | 472 | 60,944,077 | 4.80% |
| **genome size** | NA | 1,268,611,172 | NA |
| **genome size without N** | NA | 1,268,527,555 | NA |
| **GCcontent** | NA | NA | 39.44% |
| **A** | 384,066,627 | NA | 30.27% |
| **T** | 384,123,405 | NA | 30.28% |
| **G** | 250,101,397 | NA | 19.71% |
| **C** | 250,236,126 | NA | 19.73% |
| **N** | 83,617 | NA | 0.01% |
| **Others** | 0 | NA | 0.00% |
| **contig** | 1,407 | NA | NA |
| **contig Max** | NA | 13,524,544 | NA |
| **contig Mean** | NA | 901,583 | NA |
| **contig N10** | NA | 9,005,691 | NA |
| **contig N50** | NA | 2,612,587 | NA |
| **contig N90** | NA | 388,899 | NA |
| **contig Min** | NA | 25,002 | NA |
| **contig Median** | NA | 293,116 | NA |
| **contig L10** | 13 | NA | NA |
| **contig L50** | 125 | NA | NA |
| **contig L90** | 611 | NA | NA |
| **scaffold** | 486 | NA | NA |
| **scaffold Max** | NA | 140,386,692 | NA |
| **scaffold Mean** | NA | 2,610,311 | NA |
| **scaffold N10** | NA | 140,386,692 | NA |
| **scaffold N50** | NA | 104,721,408 | NA |
| **scaffold N90** | NA | 66,885,416 | NA |
| **scaffold Min** | NA | 25,002 | NA |
| **scaffold Median** | NA | 110,581 | NA |
| **scaffold L10** | 1 | NA | NA |
| **scaffold L50** | 5 | NA | NA |
| **scaffold L90** | 12 | NA | NA |

**Table S3.** Statistics of the *L. megaphylla* genome.

|  | **Genome assembly** | **Genome assembly** | **Protein-coding genes** | **Protein-coding genes** |
| --- | --- | --- | --- | --- |
|  | **BUSCO groups** | **Percentage (%)** | **BUSCO groups** | **Percentage (%)** |
| **Complete BUSCOs** | 1306 | 90.69 | 1320 | 91.67 |
| **Complete and single-copy BUSCOs** | 1225 | 85.07 | 1208 | 83.89 |
| **Complete and duplicated BUSCOs** | 81 | 5.63 | 112 | 7.78 |
| **Fragmented BUSCOs** | 36 | 2.50 | 48 | 3.33 |
| **Missing BUSCOs** | 98 | 6.81 | 72 | 5.00 |
| **Total BUSCO groups searched** | 1440 | 100.00 | 1440 | 100.00 |

**Table S4.** Evaluation of genome assembly using BUSCO.

| **Class** | **Feature** |
| --- | --- |
| **Gene number** | 34,216 |
| **Protein coding gene number** | 32,586 |
| **Transcript number** | 34,216 |
| **Transcript number (AED<0.5)** | 29,400 |
| **Average gene region length (bp)** | 7693.79 |
| **Average transcript length (bp)** | 1,410.11 |
| **Average coding sequence length (bp)** | 1,250.89 |
| **Average exons per transcript** | 5.22 |
| **Average exon length (bp)** | 270.00 |
| **Average intron length (bp)** | 1,094.68 |

**Table S5.** Statistics of the predicted protein-coding genes in *L. megaphylla* genome.

| **Source** | **Gene Category** | **Gene Number** |
| --- | --- | --- |
| **maker** | mRNA | 32,586 |
| **Rfam** | ncRNA | 803 |
| **RNAmmer-1.2** | rRNA | 248 |
| **RNAmmer-1.2** | 28S rRNA | 5 |
| **RNAmmer-1.2** | 18S rRNA | 6 |
| **RNAmmer-1.2** | 5S rRNA | 237 |
| **tRNAScan-SE** | tRNA | 579 |

**Table S6**. Statistics of the non-coding RNA genes in *L. megaphylla* genome.

|  | **Databases** | **Count** | **Percentage (%)** |
| --- | --- | --- | --- |
| **Total genes** |  | 32,586 | 100.00 |
| **Blat** | Swiss_Prot | 19,677 | 60.40 |
| **Blat** | TrEMBL | 28,641 | 87.90 |
| **Blat** | NR | 28,996 | 89.00 |
| **Blat** | Pfam | 24,936 | 76.50 |
| **Blat** | eggNOG | 27,647 | 84.80 |
| **Blat** | GO | 19,462 | 59.70 |
| **Blat** | KO | 12,075 | 37.10 |
| **Blat** | Unannotated | 3,554 | 10.90 |
| **interProScan** | TIGRFAM | 2,743 | 8.42 |
| **interProScan** | PANTHER | 28,938 | 88.81 |
| **interProScan** | Gene3D | 20,755 | 63.69 |
| **interProScan** | PRINTS | 3,908 | 11.99 |
| **interProScan** | SignalP_EUK | 2,699 | 8.28 |
| **interProScan** | ProSitePatterns | 5,353 | 16.43 |
| **interProScan** | Coils | 5,005 | 15.36 |
| **interProScan** | Pfam | 24,770 | 76.01 |
| **interProScan** | SUPERFAMILY | 19,245 | 59.06 |
| **interProScan** | SignalP_GRAM_POSITIVE | 1,901 | 5.83 |
| **interProScan** | SFLD | 222 | 0.68 |
| **interProScan** | MobiDBLite | 13,340 | 40.94 |
| **interProScan** | GO | 19,124 | 58.69 |
| **interProScan** | ProSiteProfiles | 10,656 | 32.70 |
| **interProScan** | TMHMM | 6,945 | 21.31 |
| **interProScan** | ProDom | 323 | 0.99 |
| **interProScan** | SMART | 8,203 | 25.17 |
| **interProScan** | CDD | 9,099 | 27.92 |
| **interProScan** | SignalP_GRAM_NEGATIVE | 887 | 2.72 |
| **interProScan** | Phobius | 11,037 | 33.87 |
| **interProScan** | PIRSF | 1,515 | 4.65 |
| **interProScan** | Hamap | 714 | 2.19 |
| **interProScan** | IPR | 26,747 | 82.08 |
| **interProScan** | Reactome | 3,166 | 9.72 |
| **interProScan** | KEGG | 2,172 | 6.67 |
| **interProScan** | MetaCyc | 1,584 | 4.86 |
| **interProScan** | Unannotated | 1,175 | 3.21 |

**Table S7.** Statistics functional annotation of protein-coding genes in *L. megaphylla* genome.

| **Species** | **Genes** | **Genome size (Mb)** | **Scaffold N50** | **References (DOI)** |
| --- | --- | --- | --- | --- |
| ***Amorella trichopoda*** | 26,846 | 706 M | 4.9 Mb | 10.1126/science.1241089 |
| ***Nymphaea colorata*** | 31,589 | 409 M | 255 Mb | 10.1038/s41586-019-1852-5 |
| ***Piper nigrum*** | 63,466 | 761 M | 29 Mb | 10.1038/s41467-019-12607-6 |
| ***Liriodendron chinense*** | 35,535 | 1.7 G | 3.5 Mb | 10.1038/s41477-018-0323-6 |
| ***Persea americana*** | 24,616 | 912 M | 296 Kb | 10.1073/pnas.1822129116 |
| ***Cinnamomum kanehirae*** | 26,531 | 730 M | 50 Mb | 10.1038/s41477-018-0337-0 |
| ***‎Litsea cubeba*** | 31,329 | 1370.14Mb | 607.34kb | 10.1038/s41467-020-15493-5 |
| ***Zostera marina*** | 20,762 | 203 M | 485 Kb | 10.1038/nature16548 |
| ***Sorghum bicolor*** | 32,223 | 818M | 33.28 Mb | 10.1038/s41467-018-07271-1 |
| ***Oryza sativa*** | 42,189 | 372 M | 30 Mb | 10.1038/s41467-018-07669-x |
| ***Musa acuminata*** | 35,276 | 451 M | 37.6 Mb | 10.1038/nature11241 |
| ***Aquilegia coerulea*** | 30,023 | 292 M | 43.6 Mb | 10.7554/eLife.36426 |
| ***Populus trichocarpa*** | 41,335 | 422.9 M | 20 Mb | 10.1126/science.1128691 |
| ***Arabidopsis thaliana*** | 27,416 | 135 M | 24 Mb | 10.1038/s41467-018-03016-2 |
| ***Coffea canephora*** | 25,574 | 568.6 M | 1.26 Mb | 10.1126/science.1255274 |
| ***Solanum lycopersicum*** | 34,727 | 900M | 66.5Mb | 10.1038/nature11119 |
| ***Cajanus cajan*** | 48,680 | 833.07 M | 516.06 Kb | 10.1038/nbt.2022 |

**Table S8.** Statistics of the genomic data used for phylogenomic and gene family analyses.

| **Order** | **Superfamily** | **Number** | **Length (bp)** | **Percent (%)** | **Mean_length (bp)** |
| --- | --- | --- | --- | --- | --- |
| LTR |  | 396,218 | 510,562,817 | 40.25 | 1,288.59 |
| LTR | Cassandra | 1,110 | 108,401 | 0.01 | 97.65855856 |
| LTR | Caulimovirus | 894 | 602,558 | 0.05 | 674.0022371 |
| LTR | Copia | 135,365 | 164,465,538 | 12.96 | 1,214.98 |
| LTR | ERV1 | 502 | 311,892 | 0.02 | 621.2988048 |
| LTR | Gypsy | 238,838 | 338,717,729 | 26.70 | 1418.190275 |
| LTR | Pao | 7,199 | 4,131,560 | 0.33 | 573.9074872 |
| LINE |  | 92,039 | 34,972,781 | 2.76 | 379.9778463 |
| LINE | CRE | 798 | 949,752 | 0.07 | 1,190.17 |
| LINE | I-Jockey | 2,623 | 709,930 | 0.06 | 270.6557377 |
| LINE | L1 | 29,048 | 15,621,706 | 1.23 | 537.7893831 |
| LINE | L1-DRE | 7,131 | 959,347 | 0.08 | 134.531903 |
| LINE | L1-Tx1 | 6,235 | 2,133,278 | 0.17 | 342.1456295 |
| LINE | L2 | 4,338 | 3,093,170 | 0.24 | 713.0405717 |
| LINE | R1 | 1,440 | 340,991 | 0.03 | 236.7993056 |
| LINE | RTE-BovB | 40,426 | 11,164,607 | 0.88 | 276.1739227 |
| SINE |  | 12,071 | 1,736,624 | 0.14 | 143.8674509 |
| SINE | Alu | 5,665 | 1,103,514 | 0.09 | 194.7950574 |
| SINE | tRNA | 4,799 | 430,249 | 0.03 | 89.65388623 |
| SINE | tRNA-Meta | 222 | 21,845 | 0.00 | 98.4009009 |
| SINE | tRNA-RTE | 1,385 | 181,016 | 0.01 | 130.6974729 |
| DNA |  | 170,434 | 75,930,781 | 5.99 | 445.5142812 |
| DNA | CMC-EnSpm | 33,002 | 21,776,339 | 1.72 | 659.8490698 |
| DNA | CMC-Transib | 3,761 | 3,222,406 | 0.25 | 856.7950013 |
| DNA | Crypton-S | 752 | 124,533 | 0.01 | 165.6023936 |
| DNA | Kolobok-T2 | 3,572 | 4,408,794 | 0.35 | 1234.264838 |
| DNA | MULE-MuDR | 16,210 | 7,758,923 | 0.61 | 478.650401 |
| DNA | Maverick | 486 | 42,698 | 0.00 | 87.85596708 |
| DNA | MuLE-MuDR | 5,146 | 5,728,950 | 0.45 | 1,113.28 |
| DNA | Novosib | 544 | 234,117 | 0.02 | 430.3621324 |
| DNA | P | 1,875 | 229,742 | 0.02 | 122.5290667 |
| DNA | PIF-Harbinger | 3,668 | 962,659 | 0.08 | 262.447928 |
| DNA | hAT | 2,398 | 404,075 | 0.03 | 168.5050042 |
| DNA | hAT-Ac | 65,743 | 16,679,465 | 1.31 | 253.7070867 |
| DNA | hAT-Tag1 | 17,644 | 9,110,907 | 0.72 | 516.3742349 |
| DNA | hAT-Tip100 | 15,087 | 5,043,819 | 0.40 | 334.3155697 |
| RC |  | 9,779 | 4,845,115 | 0.38 | 495.4611924 |
| RC | Helitron | 9,779 | 4,845,115 | 0.38 | 495.4611924 |
| Unknown |  | 622,289 | 201,625,959 | 15.89 | 324.0069469 |
| Satellite |  | 14,106 | 6,251,741 | 0.49 | 443.1972919 |
| Simple_repeat |  | 260,067 | 11,327,626 | 0.89 | 43.55656812 |
| Low_complexity |  | 46,382 | 2,403,026 | 0.19 | 51.80945194 |
| total |  | 1,623,385 | 849,656,470 | 66.98 | 523.3856849 |

**Table S9.** Annotation of the repeat sequences in the genome assembly of *L. megaphylla*.

| **Species** | **Intact LTR-RT (*I*)** | **Cluster number** | **Solo-LTR (*S*)** | **Truncated LTR (*T*)** | ***S*+*T*** | ***I*+*S*+*T*** | **Filtered scaffold length (kb)** | **Filtered *I*** | **Filtered *S*** | **Filtered *T*** | **Filtered *S*/*I*** | **Filter *T*/*I*** | **Filter (*S*+*T*)/*I*** |
| --- | --- | --- | --- | --- | --- | --- | --- | --- | --- | --- | --- | --- | --- |
| ***Lindera megaphylla*** | 10,407 | 1,355 | 30,304 | 101,153 | 131,457 | 141,864 | 300 | 9,958 | 28,612 | 95,872 | 2.873267724 | 9.627636072 | 12.5009038 |
| ***Nymphaea colorata*** | 1,632 | 453 | 704 | 4,275 | 4,979 | 6,611 | 60 | 1,618 | 621 | 4,117 | 0.383807169 | 2.544499382 | 2.928306551 |
| ***Persea americana*** | 4,415 | 1,061 | 16,571 | 27,455 | 44,026 | 48,441 | 140 | 3,154 | 8,563 | 17,213 | 2.714965124 | 5.457514268 | 8.172479391 |
| ***Piper nigrum*** | 3,010 | 491 | 18,598 | 48,948 | 67,546 | 70,556 | 40 | 3,010 | 18,584 | 48,940 | 6.174086379 | 16.25913621 | 22.43322259 |
| ***Cajanus cajan*** | 2,233 | 1,194 | 10,701 | 8,641 | 19,342 | 21,575 | 125 | 1,790 | 6,844 | 6,014 | 3.823463687 | 3.359776536 | 7.183240223 |
| ***Cinnamomum kanehirae*** | 3,205 | 977 | 11,465 | 18,267 | 29,732 | 32,937 | 275 | 3,045 | 8,632 | 14,331 | 2.834811166 | 4.706403941 | 7.541215107 |
| ***Coffea canephora*** | 2,055 | 765 | 11,938 | 9,291 | 21,229 | 23,284 | 0 | 2,055 | 11,938 | 9,291 | 5.809245742 | 4.521167883 | 10.33041363 |
| ***Liriodendron chinense*** | 19,401 | 2,104 | 55,886 | 153,679 | 209,565 | 228,966 | 900 | 16,686 | 46,301 | 132,982 | 2.774841184 | 7.969675177 | 10.74451636 |
| ***Musa acuminata*** | 1,420 | 326 | 19,120 | 20,380 | 39,500 | 40,920 | 0 | 1,420 | 19,120 | 20,380 | 13.46478873 | 14.35211268 | 27.81690141 |
| ***Oryza sativa*** | 2,672 | 489 | 5,936 | 7,029 | 12,965 | 15,637 | 0 | 2,672 | 5,936 | 7,029 | 2.221556886 | 2.630613772 | 4.852170659 |
| ***Populus trichocarpa*** | 1,797 | 806 | 6,249 | 7,300 | 13,549 | 15,346 | 215 | 1,684 | 5,361 | 6,763 | 3.183491686 | 4.016033254 | 7.199524941 |
| ***Solanum lycopersicum*** | 6,826 | 1,895 | 15,686 | 27,135 | 42,821 | 49,647 | 0 | 6,826 | 15,686 | 27,135 | 2.297978318 | 3.975241723 | 6.273220041 |
| ***Sorghum bicolor*** | 9,313 | 1,114 | 4,161 | 6,909 | 11,070 | 20,383 | 1,175 | 9,123 | 918 | 3,876 | 0.100624794 | 0.424860243 | 0.525485038 |
| ***Vitis vinifera*** | 4,116 | 915 | 7,734 | 15,339 | 23,073 | 27,189 | 0 | 4,116 | 7,734 | 15,339 | 1.879008746 | 3.726676385 | 5.605685131 |
| ***Zostera marina*** | 755 | 325 | 7,386 | 12,116 | 19,502 | 20,257 | 110 | 695 | 4,718 | 10,229 | 6.788489209 | 14.71798561 | 21.50647482 |
| ***Amorella trichopoda*** | 1,190 | 721 | 6,009 | 4,539 | 10,548 | 11,738 | 55 | 1,181 | 5,402 | 4,408 | 4.574089754 | 3.732430144 | 8.306519898 |
| ***Aquilegia coerulea*** | 2,288 | 370 | 8,213 | 10,905 | 19,118 | 21,406 | 225 | 2,255 | 7,630 | 10,670 | 3.383592018 | 4.731707317 | 8.115299335 |
| ***Arabidopsis thaliana*** | 299 | 205 | 275 | 450 | 725 | 1,024 | 0 | 299 | 275 | 450 | 0.919732441 | 1.505016722 | 2.424749164 |

**Table S10.** Summary of superfamilies of the Gypsy and Copia LTR-RTs in *L. megaphylla* genome.

|  | ***A. trichopoda*** | ***N. colorata*** | ***Z. marina*** | ***O. sativa*** | ***S. bicolor*** | ***M. acuminata*** | ***P. nigrum*** | ***L. chinense*** | ***L. cubeba*** |
| --- | --- | --- | --- | --- | --- | --- | --- | --- | --- |
| **WGD** | 176 (0.70%) | 4,478 (15.70%) | 136 ( 0.70%) | 5,947 (14.10%) | 5,308 (15.60%) | 15,999 (45.40%) | 32,557 (51.30%) | 3,833 (10.90%) | 6,417 (20.90%) |
| **TD** | 2,126 (7.90%) | 1,741 (6.10%) | 1,062 (5.10%) | 3,838 (9.10%) | 3,903 (11.40%) | 1,396 (4.00%) | 2,235 (3.50%) | 3,178 (9.00%) | 2,533 (8.27%) |
| **PD** | 1,824 (6.80%) | 1,955 (6.90%) | 660 (3.20%) | 3,146 (7.50%) | 1,883 (5.50%) | 783 (2.20%) | 4,651 (7.30%) | 2,366 (6.70%) | 3,093 (10.09%) |
| **TRD** | 5,916 (22.00%) | 6,067 (21.30%) | 4,843 (23.5%) | 6,795 (16.10%) | 6,627 (19.40%) | 7,918 (22.40%) | 7,637 (12.00%) | 6,902 (19.60%) | 7,802 (25.50%) |
| **DSD** | 7,463 (27.80%) | 6,953 (24.40%) | 8,802 (42.6%) | 10,189 (24.20%) | 7,134 (20.90%) | 3,565 (10.10%) | 4,415 (7.00%) | 14,689 (41.60%) | 6,106 (19.90%) |
| **SL** | 9,341 (34.80%) | 7,244 (25.50%) | 5,137 (24.90%) | 12,274 (29.10%) | 9,274 (27.20%) | 5,615 (15.90%) | 11,971 (18.9%) | 4,301 (12.20%) | 4,688 (15.30%) |

|  | ***L. megaphylla*** | ***C. kanehirae*** | ***A. coerulea*** | ***S. lycopersicum*** | ***C. canephora*** | ***C. cajan*** | ***P. trichocarpa*** | ***A. thaliana*** |
| --- | --- | --- | --- | --- | --- | --- | --- | --- |
| **WGD** | 7,364 (22.60%) | 7,246 (27.30%) | 1,888 (6.30%) | 6,530 (18.80%) | 2,742 (10.70%) | 3,894 (13.40%) | 21,881 (52.90%) | 7,458 (27.20%) |
| **TD** | 3,313 (10.20%) | 3,562 (13.40%) | 3,400 (11.30%) | 3,510 (10.11) | 2,872 (11.20%) | 3,236 (11.10%) | 3,162 (7.60%) | 3,003 (11.00%) |
| **PD** | 3,195 (9.80%) | 2,394 (9.00%) | 2,121 (7.10%) | 1,912 (5.51%) | 1,688 (6.60%) | 1,418 (4.90%) | 2,569 (6.20%) | 1,057 (3.90%) |
| **TRD** | 6,539 (20.10%) | 4,847 (18.30%) | 7,095 (23.60%) | 6,822 (19.6%) | 6,390 (25.00%) | 8,780 (30.20%) | 4,282 (10.40%) | 5,474 (20.00%) |
| **DSD** | 8,427 (25.90%) | 4,569 (17.20%) | 8,619 (28.70%) | 7,705 (22.2%) | 6,306 (24.70%) | 8,017 (27.50%) | 3,738 (9.00%) | 4,930 (18.00%) |
| **SL** | 3,748 (11.50%) | 3,913 (14.70%) | 6,900 (23.00%) | 8,246(23.70%) | 5,576 (21.80%) | 3,774 (13.00%) | 5,703 (13.80%) | 5,494 (20.00%) |

**Table S11.** Number and proportion of whole-genome duplicated (WGD), tandem duplicated (TD), proximal duplicated (PD), dispersed duplicated (DSD), transposed duplicated (TRD) and singleton genes in the genomes of 17 species.

|  | ***A. trichopoda*** | ***N. colorata*** | ***Z. marina*** | ***O. sativa*** | ***S. bicolor*** | ***M. acuminata*** | ***P. nigrum*** | ***L. chinense*** | ***L. cubeba*** |
| --- | --- | --- | --- | --- | --- | --- | --- | --- | --- |
| **TD** | 2126 (7.92%) | 1741 (6.12%) | 1062 (5.14%) | 3838 (9.10%) | 3903 (11.44%) | 1396 (3.96%) | 2235 (3.52%) | 3178 (9.01%) | 2533 (8.27%) |
| **PD** | 1824 (6.79%) | 1955 (6.87%) | 660 (3.20) | 3146 (7.46%) | 1883 (5.52%) | 783 (2.22%) | 4651 (7.33%) | 2366 (6.71%) | 3093 (10.09%) |
| **TD\|PD** | 3950 (14.71%) | 3696 (12.99%) | 1722 (8.34%) | 6984 (16.56%) | 5786 (16.96%) | 2179 (6.18%) | 6886 (10.85%) | 5544 (15.72%) | 5626 (18.36%) |
| **ALL** | 26,846 | 28438 | 20641 | 42,190 | 34130 | 35,277 | 63,467 | 35,270 | 30,640 |

|  | ***L. megaphylla*** | ***C. kanehirae*** | ***A. coerulea*** | ***S. lycopersicum*** | ***C. canephora*** | ***C. cajan*** | ***P. trichocarpa*** | ***A. thaliana*** |
| --- | --- | --- | --- | --- | --- | --- | --- | --- |
| **TD** | 3313 (10.17%) | 3562 (13.43%) | 3400 (11.32%) | 3510 (10.11%) | 2872 (11.23%) | 3236 (11.11%) | 3162 (7.65%) | 3003 (10.95%) |
| **PD** | 3195 (9.80%) | 2394 (9.02%) | 2121 (7.06%) | 1912 (5.51%) | 1688 (6.60%) | 1418 (4.87%) | 2569 (6.21%) | 1057 (3.86%) |
| **TD\|PD** | 6508 (19.97%) | 5956 (22.45%) | 5521 (18.39%) | 5422 (15.61%) | 4560 (17.83%) | 4654 (15.98%) | 5731 (13.86%) | 4060 (14.81%) |
| **ALL** | 32,587 | 26532 | 30,023 | 34726 | 25,575 | 29,120 | 41,336 | 27417 |

**Table S12.** Number and proportion of tandem (TD) or proximal (PD) duplicated genes in the genomes of 17 species. All, all identified genes; TD, tandem duplicated genes; PD, proximal duplicated genes.

| **Biosynthesis** | **Enzyme** | **Species** | **Genes** |
| --- | --- | --- | --- |
| **(S)-reticuline biosynthesis I** | **TyrAT** | *L. megaphylla* | Limeg01G0028700,Limeg12G0123900,Limeg12G0124000,Limeg12G0124100 |
| **(S)-reticuline biosynthesis I** | **PDC** | *L. megaphylla* | Limeg02G0023900,Limeg02G0235000,Limeg02G0327500,Limeg04G0092500,  Limeg06G0214200,Limeg08G0046800,Limeg08G0047000,Limeg09G0118400,  Limeg10G0123400,Limeg10G0123500 |
| **(S)-reticuline biosynthesis I** | **NCS** | *L. megaphylla* | Limeg07G0008300,Limeg07G0015200,Limeg07G0015300 |
| **(S)-reticuline biosynthesis I** | **6OMT** | *L. megaphylla* | Limeg01G0266300,Limeg01G0267000,Limeg03G0295000,Limeg09G0081700,  Limeg09G0098800,Limeg09G0098900,LimegUnG0068200,LimegUnG0068500 |
| **(S)-reticuline biosynthesis I** | **CNMT** | *L. megaphylla* | Limeg02G0119000,Limeg02G0119100,Limeg02G0119300,Limeg02G0119500,  Limeg02G0119800,Limeg02G0120000,Limeg02G0120100,Limeg02G0120200,  Limeg02G0120300,Limeg02G0120400,Limeg02G0152700,Limeg02G0152900 |
| **(S)-reticuline biosynthesis I** | **CYP80B** | *L. megaphylla* | Limeg03G0046700,Limeg03G0046900,Limeg03G0047000,Limeg03G0295100,  Limeg03G0295200 |
| **(S)-reticuline biosynthesis I** | **4OMT** | *L. megaphylla* | Limeg09G0099100,Limeg09G0099200,Limeg09G0099300,LimegUnG0067900,  LimegUnG0077000 |
| **magnoflorine biosynthesis** | **CYP80G** | *L. megaphylla* | Limeg01G0380500,Limeg01G0380800,Limeg01G0381000,Limeg01G0381200,  Limeg02G0205100,Limeg02G0205300,Limeg06G0245300,Limeg08G0048800,  Limeg08G0049100,Limeg08G0049400,Limeg08G0049500,Limeg08G0049900 |
| **magnoflorine biosynthesis** | **RNMT** | *L. megaphylla* | Limeg02G0119000,Limeg02G0119100,Limeg02G0119300,Limeg02G0119500,  Limeg02G0119800,Limeg02G0120000,Limeg02G0120100,Limeg02G0120200,  Limeg02G0120300,Limeg02G0120400,Limeg02G0152700,Limeg02G0152900 |
| **berberine biosynthesis** | **BBE** | *L. megaphylla* | Limeg03G0061500,Limeg04G0350000,Limeg04G0350200,Limeg12G0065500,  Limeg12G0065700 |
| **berberine biosynthesis** | **SOMT** | *L. megaphylla* | Limeg09G0012400,Limeg04G0334700 |
| **berberine biosynthesis** | **CYP719A** | *L. megaphylla* | Limeg02G0276000,Limeg08G0194200,Limeg08G0194700,Limeg08G0193900,  Limeg08G0195100 |
| **berberine biosynthesis** | **STOX** | *L. megaphylla* | Limeg07G0139500,LimegUnG0108100 |
| **palmatine biosynthesis** | **CoOMT** | *L. megaphylla* | Limeg03G0262500,Limeg03G0262600,Limeg03G0262700,Limeg03G0262900,  Limeg03G0263100,Limeg03G0263300,Limeg03G0263400,Limeg03G0263600,  Limeg03G0263700,Limeg03G0263800,Limeg03G0263900,Limeg03G0264000,  Limeg03G0264300,Limeg03G0264400,Limeg03G0264500,Limeg03G0264700 |
| **(S)-reticuline biosynthesis I** | **TyrAT** | *C. kanehirae* | CKAN_02334600,CKAN_02334700,CKAN_02334800 |
| **(S)-reticuline biosynthesis I** | **PDC** | *C. kanehirae* | CKAN_00445400,CKAN_00742500,CKAN_00832900,CKAN_01001900,  CKAN_01339500,CKAN_02032900,CKAN_02135600,CKAN_02574700,  CKAN_02575300 |
| **(S)-reticuline biosynthesis I** | **NCS** | *C. kanehirae* | CKAN_00799000,CKAN_01029100,CKAN_01875800,CKAN_01875900,  CKAN_01881600 |
| **(S)-reticuline biosynthesis I** | **6OMT** | *C. kanehirae* | CKAN_00132100,CKAN_00132600,CKAN_01613100,CKAN_02386100 |
| **(S)-reticuline biosynthesis I** | **CNMT** | *C. kanehirae* | CKAN_00893900,CKAN_00894100,CKAN_00919600,CKAN_00919700,  CKAN_00919900 |
| **(S)-reticuline biosynthesis I** | **CYP80B** | *C. kanehirae* | CKAN_01613200,CKAN_01655600,CKAN_01655700,CKAN_01655800,  CKAN_01655900 |
| **(S)-reticuline biosynthesis I** | **4OMT** | *C. kanehirae* | CKAN_00132300,CKAN_00132700,CKAN_02149300 |
| **magnoflorine biosynthesis** | **CYP80G** | *C. kanehirae* | CKAN_00036900,CKAN_01319400,CKAN_02031600,CKAN_02032500 |
| **magnoflorine biosynthesis** | **RNMT** | *C. kanehirae* | CKAN_00893900,CKAN_00894100,CKAN_00919600,CKAN_00919700,  CKAN_00919900 |
| **berberine biosynthesis** | **BBE** | *C. kanehirae* | CKAN_00678300,CKAN_01669000,CKAN_01669300,CKAN_01669400,  CKAN_01669700,CKAN_01669800,CKAN_02285700 |
| **berberine biosynthesis** | **SOMT** | *C. kanehirae* | CKAN_00657200,CKAN_02222700,CKAN_02222800 |
| **berberine biosynthesis** | **CYP719A** | *C. kanehirae* | CKAN_00793800,CKAN_01917700,CKAN_01917800,CKAN_01917900,  CKAN_01918100,CKAN_01918200,CKAN_01918300,CKAN_01918400,  CKAN_01918500,CKAN_01918800,CKAN_01918900,CKAN_01919000,  CKAN_01919100,CKAN_01919300,CKAN_01919400,CKAN_01919500,  CKAN_01919700,CKAN_01919800,CKAN_02119900,CKAN_02120200 |
| **berberine biosynthesis** | **STOX** | *C. kanehirae* | CKAN_01769600 |
| **palmatine biosynthesis** | **CoOMT** | *C. kanehirae* | CKAN_01584400,CKAN_01584500,CKAN_01584600 |
| **(S)-reticuline biosynthesis I** | **TyrAT** | *L. cubeba* | Lcu10G_26511 |
| **(S)-reticuline biosynthesis I** | **PDC** | *L. cubeba* | Lcu02G_07976,Lcu04G_11283,Lcu04G_12462,Lcu04G_13368,Lcu04G_14423,  Lcu06G_20277,Lcu11G_28716,Lcu11G_28723,Lcu11G_28724,Lcu11G_28725,  Lcu12G_29947 |
| **(S)-reticuline biosynthesis I** | **NCS** | *L. cubeba* | Lcu05G_17830,Lcu08G_24436 |
| **(S)-reticuline biosynthesis I** | **6OMT** | *L. cubeba* | Lcu01G_01319,Lcu03G_09085,Lcu03G_09086,Lcu07G_21485,Lcu11G_28564 |
| **(S)-reticuline biosynthesis I** | **CNMT** | *L. cubeba* | Lcu04G_11326,Lcu04G_11327,Lcu04G_11328,Lcu04G_11340,Lcu04G_11341,  Lcu04G_11346,Lcu04G_11355 |
| **(S)-reticuline biosynthesis I** | **CYP80B** | *L. cubeba* | Lcu03G_09084 |
| **(S)-reticuline biosynthesis I** | **4OMT** | *L. cubeba* | Lcu01G_01324,Lcu11G_28647 |
| **magnoflorine biosynthesis** | **CYP80G** | *L. cubeba* | Lcu01G_00333,Lcu01G_00354,Lcu02G_07766,Lcu06G_20264,Lcu06G_20270 |
| **magnoflorine biosynthesis** | **RNMT** | *L. cubeba* | Lcu04G_11326,Lcu04G_11327,Lcu04G_11328,Lcu04G_11340,Lcu04G_11341,  Lcu04G_11346,Lcu04G_11355 |
| **berberine biosynthesis** | **BBE** | *L. cubeba* | Lcu01G_01586,Lcu01G_01587,Lcu05G_16417,Lcu05G_16420,Lcu05G_16421,  Lcu10G_27072,Lcu10G_27074,Lcu10G_27075,Lcu10G_27076,Lcu10G_27077,  Lcu10G_27078,Lcu10G_27079,Lcu10G_27080 |
| **berberine biosynthesis** | **SOMT** | *L. cubeba* | Lcu04G_11518Lcu04G_11519,Lcu04G_12352,Lcu11G_27843 |
| **berberine biosynthesis** | **CYP719A** | *L. cubeba* | Lcu04G_12877,Lcu06G_19046,Lcu06G_19049 |
| **berberine biosynthesis** | **STOX** | *L. cubeba* | Lcu08G_23184 |
| **palmatine biosynthesis** | **CoOMT** | *L. cubeba* | Lcu03G_09419 |
| **(S)-reticuline biosynthesis I** | **TyrAT** | *P. bournei* | Maker00017468,Maker00038763,Maker00044596 |
| **(S)-reticuline biosynthesis I** | **PDC** | *P. bournei* | Maker00001272,Maker00007497,Maker00012427,Maker00018082,Maker00018942,  Maker00022733,Maker00027458,Maker00044810 |
| **(S)-reticuline biosynthesis I** | **NCS** | *P. bournei* | Maker00048129,Maker00048133 |
| **(S)-reticuline biosynthesis I** | **6OMT** | *P. bournei* | Maker00005376,Maker00012679,Maker00032691,Maker00040996,Maker00047217,  Maker00051970,Maker00052296,Maker00053537 |
| **(S)-reticuline biosynthesis I** | **CNMT** | *P. bournei* | Maker00014537 |
| **(S)-reticuline biosynthesis I** | **CYP80B** | *P. bournei* | Maker00020558,Maker00020611,Maker00020617,Maker00052287 |
| **(S)-reticuline biosynthesis I** | **4OMT** | *P. bournei* | Maker00005399,Maker00011599,Maker00020947 |
| **magnoflorine biosynthesis** | **CYP80G** | *P. bournei* | Maker00015734,Maker00051657,Maker00052107,Maker00052123,Maker00053604 |
| **magnoflorine biosynthesis** | **RNMT** | *P. bournei* | Maker00014537 |
| **berberine biosynthesis** | **BBE** | *P. bournei* | Maker00016818,Maker00021925,Maker00021960,Maker00022025,Maker00022058,  Maker00022069 |
| **berberine biosynthesis** | **SOMT** | *P. bournei* | Maker00031517,Maker00031567,Maker00052956 |
| **berberine biosynthesis** | **CYP719A** | *P. bournei* | . |
| **berberine biosynthesis** | **STOX** | *P. bournei* | Maker00033930,Maker00033955 |
| **palmatine biosynthesis** | **CoOMT** | *P. bournei* | Maker00024087,Maker00036729,Maker00037105 |

**Table S13**. Gene annotation of three isoquinoline alkaloid biosynthesis pathways in four Lauraceae species.

| **Enzyme** | **TDPD** | ***A. trichopoda*** | ***N. colorata*** | ***P. nigrum*** | ***L. chinense*** | ***C. kanehirae*** | ***L. megaphylla*** | ***L. cubeba*** | ***A. coerulea*** | ***P. trichocarpa*** |
| --- | --- | --- | --- | --- | --- | --- | --- | --- | --- | --- |
| **TyrAT** | **All** | 2 | 2 | 2 | 5 | 3 | 4 | 1 | 2 | 6 |
|  | **TD** | 0 (0.00%) | 0 (0.00%) | 0 (0.00%) | 2 （40.00%） | 3 (100.00%) | 3 (75.00%) | 0 (0.00%) | 0 (0.00%) | 2 (33.33%) |
|  | **PD** | 0 (0.00%) | 0 (0.00%) | 0 (0.00%) | 1 （20.00%） | 0 (0.00%) | 0 (0.00%) | 0 (0.00%) | 2 (100.00%) | 0 (0.00%) |
|  | **TDPD** | 0 (0.00%) | 0 (0.00%) | 0 (0.00%) | 3 （60.00%） | 3 (100.00%) | 3 (75.00%) | 0 (0.00%) | 2 (100.00%) | 2 (33.33%) |
| **NCS** | **All** | 2 | 0 | 37 | 10 | 5 | 3 | 2 | 21 | 5 |
|  | **TD** | 2 (100.00%) | 0 (0.00%) | 21 (56.76%) | 5 (50.00%) | 2 (40.00%) | 2 (66.67%) | 0 (0.00%) | 8 (38.10%) | 2 (40.00%) |
|  | **PD** | 0 (0.00%) | 0 (0.00%) | 8 (21.62%) | 2 (20.00%) | 0 (0.00%) | 0 (0.00%) | 0 (0.00%) | 5 (23.81%) | 0 (0.00%) |
|  | **TDPD** | 2 (100.00%) | 0 (0.00%) | 29 (78.38%) | 7 (70.00%) | 2 (40.00%) | 2 (66.67%) | 0 (0.00%) | 13 (61.90%) | 2 (40.00%) |
| **6OMT** | **All** | 1 | 0 | 7 | 7 | 4 | 8 | 5 | 2 | 1 |
|  | **TD** | 0 (0.00%) | 0 (0.00%) | 4 (57.14%) | 3 (42.86%) | 0 (0.00%) | 2 (25.00%) | 2 (40.00%) | 0 (0.00%) | 0 (0.00%) |
|  | **PD** | 0 (0.00%) | 0 (0.00%) | 0 (0.00%) | 0 (0.00%) | 2 (50.00%) | 4 (50.00%) | 0 (0.00%) | 0 (0.00%) | 0 (0.00%) |
|  | **TDPD** | 0 (0.00%) | 0 (0.00%) | 4 (57.14%) | 3 (42.86%) | 2 (50.00%) | 2 (75.00%) | 2 (40.00%) | 0 (0.00%) | 0 (0.00%) |
| **CNMT** | **All** | 0 | 1 | 32 | 6 | 5 | 13 | 7 | 3 | 2 |
|  | **TD** | 0 (0.00%) | 0 (0.00%) | 15 (46.88%) | 2 (33.33%) | 2 (40.00%) | 10 (76.92%) | 5 (71.43%) | 0 (0.00%) | 0 (0.00%) |
|  | **PD** | 0 (0.00%) | 0 (0.00%) | 13 (40.63%) | 2 (33.33%) | 3 (60.00%) | 3 (23.08%) | 2 (28.57%) | 2 (66.67%) | 0 (0.00%) |
|  | **TDPD** | 0 (0.00%) | 0 (0.00%) | 28 (87.50%) | 4 (66.67%) | 5 (100.00%) | 13 (100.00%) | 7 (100.00%) | 2 (66.67%) | 0 (0.00%) |
| **CYP80B** | **All** | 0 | 3 | 17 | 3 | 6 | 5 | 1 | 4 | 2 |
|  | **TD** | 0 (0.00%) | 2 (66.67%) | 10 (58.82%) | 2 (66.67%) | 4 (66.67%) | 4 (80.00%) | 0 (0.00%) | 2 (50.00%) | 0 (0.00%) |
|  | **PD** | 0 (0.00%) | 0 (0.00%) | 2 (11.76%) | 0 (0.00%) | 0 (0.00%) | 1 (20.00%) | 0 (0.00%) | 0 (0.00%) | 0 (0.00%) |
|  | **TDPD** | 0 (0.00%) | 2 (66.67%) | 12 (70.59%) | 2 (66.67%) | 4 (66.67%) | 5 (100.00%) | 0 (0.00%) | 2 (50.00%) | 0 (0.00%) |
| **4OMT** | **All** | 1 | 0 | 3 | 2 | 3 | 17 | 3 | 1 | 0 |
|  | **TD** | 0 (0.00%) | 0 (0.00%) | 0 (0.00%) | 0 (0.00%) | 0 (0.00%) | 15 (88.24%) | 0 (0.00%) | 0 (0.00%) | 0 (0.00%) |
|  | **PD** | 0 (0.00%) | 0 (0.00%) | 0 (0.00%) | 0 (0.00%) | 2 (66.67%) | 2 (11.76%) | 0 (0.00%) | 0 (0.00%) | 0 (0.00%) |
|  | **TDPD** | 0 (0.00%) | 0 (0.00%) | 0 (0.00%) | 0 (0.00%) | 2 (66.67%) | 17 (100.00%) | 0 (0.00%) | 0 (0.00%) | 0 (0.00%) |
| **CYP80G** | **All** | 0 | 3 | 17 | 3 | 6 | 5 | 1 | 4 | 2 |
|  | **TD** | 0 (0.00%) | 2 (66.67%) | 10 (58.52%) | 2 (66.67%) | 4 (66.67%) | 4 (80.00%) | 0 (0.00%) | 2 (50.00%) | 0 (0.00%) |
|  | **PD** | 0 (0.00%) | 0 (0.00%) | 2 (11.76%) | 0 (0.00%) | 0 (0.00%) | 1 (20.00%) | 0 (0.00%) | 0 (0.00%) | 0 (0.00%) |
|  | **TDPD** | 0 (0.00%) | 2 (66.67%) | 12 (70.59%) | 2 (66.67%) | 4 (66.67%) | 5 (100.00%) | 0 (0.00%) | 2 (50.00%) | 0 (0.00%) |
| **BBE** | **All** | 3 | 1 | 4 | 13 | 7 | 6 | 13 | 4 | 1 |
|  | **TD** | 2 (66.67%) | 0 (0.00%) | 0 (0.00%) | 6 (46.15%) | 4 (57.14%) | 0 (0.00%) | 11 (84.62%) | 0 (0.00%) | 0 (0.00%) |
|  | **PD** | 0 (0.00%) | 0 (0.00%) | 0 (0.00%) | 4 (30.77%) | 1 (14.29%) | 5 (83.33%) | 2 (15.38%) | 0 (0.00%) | 0 (0.00%) |
|  | **TDPD** | 2 (66.67%) | 0 (0.00%) | 0 (0.00%) | 10 (76.92%) | 5 (71.43%) | 5 (83.33%) | 13 (100.00%) | 0 (0.00%) | 0 (0.00%) |
| **SOMT** | **All** | 0 | 0 | 0 | 6 | 3 | 4 | 3 | 2 | 0 |
|  | **TD** | 0 (0.00%) | 0 (0.00%) | 0 (0.00%) | 3 (50.00%) | 2 (66.67%) | 0 (0.00%) | 0 (0.00%) | 0 (0.00%) | 0 (0.00%) |
|  | **PD** | 0 (0.00%) | 0 (0.00%) | 0 (0.00%) | 2 (33.33%) | 0 (0.00%) | 0 (0.00%) | 0 (0.00%) | 0 (0.00%) | 0 (0.00%) |
|  | **TDPD** | 0 (0.00%) | 0 (0.00%) | 0 (0.00%) | 5 (83.33%) | 2 (66.67%) | 0 (0.00%) | 0 (0.00%) | 0 (0.00%) | 0 (0.00%) |
| **CYP719A** | **All** | 0 | 0 | 4 | 0 | 20 | 5 | 3 | 8 | 0 |
|  | **TD** | 0 (0.00%) | 0 (0.00%) | 2 (50.00%) | 0 (0.00%) | 19 (95.00%) | 0 (0.00%) | 0 (0.00%) | 0 (0.00%) | 0 (0.00%) |
|  | **PD** | 0 (0.00%) | 0 (0.00%) | 0 (0.00%) | 0 (0.00%) | 0 (0.00%) | 4 (80.00%) | 2 (66.67%) | 4 (50.00%) | 0 (0.00%) |
|  | **TDPD** | 0 (0.00%) | 0 (0.00%) | 2 (50.00%) | 0 (0.00%) | 19 (95.00%) | 4 (80.00%) | 2 (66.67%) | 4 (50.00%) | 0 (0.00%) |
| **CoOMT** | **All** | 0 | 0 | 0 | 1 | 3 | 17 | 1 | 3 | 0 |
|  | **TD** | 0 (0.00%) | 0 (0.00%) | 0 (0.00%) | 0 (0.00%) | 3 (100.00%) | 15 (88.24%) | 0 (0.00%) | 2 (66.67%) | 0 (0.00%) |
|  | **PD** | 0 (0.00%) | 0 (0.00%) | 0 (0.00%) | 0 (0.00%) | 0 (0.00%) | 2 (11.76%) | 0 (0.00%) | 0 (0.00%) | 0 (0.00%) |
|  | **TDPD** | 0 (0.00%) | 0 (0.00%) | 0 (0.00%) | 0 (0.00%) | 3 (100.00%) | 17 (100.00%) | 0 (0.00%) | 2 (66.67%) | 0 (0.00%) |

| **Enzyme** | **TDPD** | ***A. thaliana*** | ***C. cajan*** | ***S. lycopersicum*** | ***C. canephora*** | ***O. sativa*** | ***S. bicolor*** | ***M. acuminata*** | ***Z. marina*** |
| --- | --- | --- | --- | --- | --- | --- | --- | --- | --- |
| **TyrAT** | **All** | 3 | 3 | 3 | 2 | 2 | 3 | 7 | 2 |
|  | **TD** | 0 (0.00%) | 0 (0.00%) | 0 (0.00%) | 0 (0.00%) | 0 (0.00%) | 2 (66.67%) | 0 (0.00%) | 0 (0.00%) |
|  | **PD** | 0 (0.00%) | 0 (0.00%) | 0 (0.00%) | 0 (0.00%) | 0 (0.00%) | 0 (0.00%) | 0 (0.00%) | 0 (0.00%) |
|  | **TDPD** | 0 (0.00%) | 0 (0.00%) | 0 (0.00%) | 0 (0.00%) | 0 (0.00%) | 2 (66.67%) | 0 (0.00%) | 0 (0.00%) |
| **NCS** | **All** | 0 | 3 | 3 | 14 | 14 | 22 | 14 | 0 |
|  | **TD** | 0 (0.00%) | 0 (0.00%) | 2 (66.67%) | 2 (14.29%) | 7 (50.00%) | 7 (31.82%) | 8 (57.14%) | 0 (0.00%) |
|  | **PD** | 0 (0.00%) | 0 (0.00%) | 0 (0.00%) | 4 (28.57%) | 5 (35.71%) | 6 (27.27%) | 2 (14.29%) | 0 (0.00%) |
|  | **TDPD** | 0 (0.00%) | 0 (0.00%) | 2 (66.67%) | 6 (42.86%) | 12 (85.71%) | 13 (59.09%) | 10 (71.43%) | 0 (0.00%) |
| **6OMT** | **All** | 0 | 1 | 0 | 0 | 0 | 0 | 0 | 0 |
|  | **TD** | 0 (0.00%) | 0 (0.00%) | 0 (0.00%) | 0 (0.00%) | 0 (0.00%) | 0 (0.00%) | 0 (0.00%) | 0 (0.00%) |
|  | **PD** | 0 (0.00%) | 0 (0.00%) | 0 (0.00%) | 0 (0.00%) | 0 (0.00%) | 0 (0.00%) | 0 (0.00%) | 0 (0.00%) |
|  | **TDPD** | 0 (0.00%) | 0 (0.00%) | 0 (0.00%) | 0 (0.00%) | 0 (0.00%) | 0 (0.00%) | 0 (0.00%) | 0 (0.00%) |
| **CNMT** | **All** | 2 | 1 | 1 | 1 | 1 | 1 | 1 | 0 |
|  | **TD** | 2 (100.00%) | 0 (0.00%) | 0 (0.00%) | 0 (0.00%) | 0 (0.00%) | 0 (0.00%) | 0 (0.00%) | 0 (0.00%) |
|  | **PD** | 0 (0.00%) | 0 (0.00%) | 0 (0.00%) | 0 (0.00%) | 0 (0.00%) | 0 (0.00%) | 0 (0.00%) | 0 (0.00%) |
|  | **TDPD** | 2 (100.00%) | 0 (0.00%) | 0 (0.00%) | 0 (0.00%) | 0 (0.00%) | 0 (0.00%) | 0 (0.00%) | 0 (0.00%) |
| **CYP80B** | **All** | 0 | 0 | 0 | 1 | 0 | 0 | 0 | 0 |
|  | **TD** | 0 (0.00%) | 0 (0.00%) | 0 (0.00%) | 0 (0.00%) | 0 (0.00%) | 0 (0.00%) | 0 (0.00%) | 0 (0.00%) |
|  | **PD** | 0 (0.00%) | 0 (0.00%) | 0 (0.00%) | 0 (0.00%) | 0 (0.00%) | 0 (0.00%) | 0 (0.00%) | 0 (0.00%) |
|  | **TDPD** | 0 (0.00%) | 0 (0.00%) | 0 (0.00%) | 0 (0.00%) | 0 (0.00%) | 0 (0.00%) | 0 (0.00%) | 0 (0.00%) |
| **4OMT** | **All** | 0 | 0 | 0 | 0 | 0 | 0 | 0 | 0 |
|  | **TD** | 0 (0.00%) | 0 (0.00%) | 0 (0.00%) | 0 (0.00%) | 0 (0.00%) | 0 (0.00%) | 0 (0.00%) | 0 (0.00%) |
|  | **PD** | 0 (0.00%) | 0 (0.00%) | 0 (0.00%) | 0 (0.00%) | 0 (0.00%) | 0 (0.00%) | 0 (0.00%) | 0 (0.00%) |
|  | **TDPD** | 0 (0.00%) | 0 (0.00%) | 0 (0.00%) | 0 (0.00%) | 0 (0.00%) | 0 (0.00%) | 0 (0.00%) | 0 (0.00%) |
| **CYP80G** | **All** | 0 | 0 | 0 | 1 | 0 | 0 | 0 | 0 |
|  | **TD** | 0 (0.00%) | 0 (0.00%) | 0 (0.00%) | 0 (0.00%) | 0 (0.00%) | 0 (0.00%) | 0 (0.00%) | 0 (0.00%) |
|  | **PD** | 0 (0.00%) | 0 (0.00%) | 0 (0.00%) | 0 (0.00%) | 0 (0.00%) | 0 (0.00%) | 0 (0.00%) | 0 (0.00%) |
|  | **TDPD** | 0 (0.00%) | 0 (0.00%) | 0 (0.00%) | 0 (0.00%) | 0 (0.00%) | 0 (0.00%) | 0 (0.00%) | 0 (0.00%) |
| **BBE** | **All** | 0 | 1 | 1 | 2 | 4 | 4 | 2 | 0 |
|  | **TD** | 0 (0.00%) | 0 (0.00%) | 0 (0.00%) | 0 (0.00%) | 3 (75.00%) | 3 (75.00%) | 0 (0.00%) | 0 (0.00%) |
|  | **PD** | 0 (0.00%) | 0 (0.00%) | 0 (0.00%) | 0 (0.00%) | 0 (0.00%) | 1 (25.00%) | 0 (0.00%) | 0 (0.00%) |
|  | **TDPD** | 0 (0.00%) | 0 (0.00%) | 0 (0.00%) | 0 (0.00%) | 3 (75.00%) | 4 (100.00%) | 0 (0.00%) | 0 (0.00%) |
| **SOMT** | **All** | 0 | 0 | 0 | 0 | 0 | 0 | 0 | 0 |
|  | **TD** | 0 (0.00%) | 0 (0.00%) | 0 (0.00%) | 0 (0.00%) | 0 (0.00%) | 0 (0.00%) | 0 (0.00%) | 0 (0.00%) |
|  | **PD** | 0 (0.00%) | 0 (0.00%) | 0 (0.00%) | 0 (0.00%) | 0 (0.00%) | 0 (0.00%) | 0 (0.00%) | 0 (0.00%) |
|  | **TDPD** | 0 (0.00%) | 0 (0.00%) | 0 (0.00%) | 0 (0.00%) | 0 (0.00%) | 0 (0.00%) | 0 (0.00%) | 0 (0.00%) |
| **CYP719A** | **All** | 0 | 0 | 0 | 0 | 0 | 0 | 0 | 0 |
|  | **TD** | 0 (0.00%) | 0 (0.00%) | 0 (0.00%) | 0 (0.00%) | 0 (0.00%) | 0 (0.00%) | 0 (0.00%) | 0 (0.00%) |
|  | **PD** | 0 (0.00%) | 0 (0.00%) | 0 (0.00%) | 0 (0.00%) | 0 (0.00%) | 0 (0.00%) | 0 (0.00%) | 0 (0.00%) |
|  | **TDPD** | 0 (0.00%) | 0 (0.00%) | 0 (0.00%) | 0 (0.00%) | 0 (0.00%) | 0 (0.00%) | 0 (0.00%) | 0 (0.00%) |
| **CoOMT** | **All** | 0 | 0 | 0 | 0 | 0 | 0 | 0 | 0 |
|  | **TD** | 0 (0.00%) | 0 (0.00%) | 0 (0.00%) | 0 (0.00%) | 0 (0.00%) | 0 (0.00%) | 0 (0.00%) | 0 (0.00%) |
|  | **PD** | 0 (0.00%) | 0 (0.00%) | 0 (0.00%) | 0 (0.00%) | 0 (0.00%) | 0 (0.00%) | 0 (0.00%) | 0 (0.00%) |
|  | **TDPD** | 0 (0.00%) | 0 (0.00%) | 0 (0.00%) | 0 (0.00%) | 0 (0.00%) | 0 (0.00%) | 0 (0.00%) | 0 (0.00%) |

**Table S14**. Number and proportion of tandem duplicated (TD) or proximal duplicated (PD) genes in isoquinoline alkaloid biosynthesis pathways. All, all genes of the relevant gene families in the isoquinoline alkaloid biosynthetic pathways.

| **Biosynthesis** | **Enzyme** | **Species** | **Genes** |
| --- | --- | --- | --- |
| General phenylpropanoid biosynthesis | PAL | *L. megaphylla* | Limeg09G0024400,Limeg05G0278500,Limeg10G0088700 |
| General phenylpropanoid biosynthesis | C4H | *L. megaphylla* | Limeg02G0310800,Limeg11G0011800,Limeg11G0011900,Limeg11G0012300 |
| General phenylpropanoid biosynthesis | 4CL | *L. megaphylla* | Limeg02G0059700,Limeg02G0339400,Limeg03G0010100,Limeg06G0209300,  Limeg06G0209800,Limeg08G0022600,Limeg08G0022700,Limeg08G0022900,  Limeg08G0145000,Limeg09G0015500,Limeg09G0015600,Limeg09G0023400 |
| flavonoid biosynthesis | CHS | *L. megaphylla* | Limeg01G0087300,Limeg07G0094500,Limeg07G0094600,Limeg10G0160900 |
| flavonoid biosynthesis | CHI | *L. megaphylla* | Limeg01G0321000,Limeg04G0206100,Limeg10G0154400,Limeg11G0015400 |
| flavonoid biosynthesis | F3H | *L. megaphylla* | Limeg03G0013000,Limeg02G0084900,Limeg12G0096400,Limeg02G0294100,  Limeg03G0193100,Limeg07G0034600,Limeg03G0013600,Limeg03G0059100,  Limeg03G0193900,Limeg08G0193600,Limeg02G0129600,Limeg03G0059300,  Limeg12G0170100,Limeg08G0210300,LimegUnG0049000,Limeg07G0034700,  Limeg04G0163900,Limeg03G0013700,Limeg08G0098500,Limeg03G0193500,  Limeg06G0281700,Limeg01G0118300,Limeg03G0013500,Limeg12G0170300,  Limeg06G0276100,LimegUnG0089000,Limeg06G0027500,Limeg03G0218000,  Limeg12G0170000,Limeg03G0126900,Limeg07G0034500,Limeg01G0118200,  Limeg03G0193800 |
| flavonoid biosynthesis | FLS | *L. megaphylla* | Limeg01G0317000,Limeg08G0193600,Limeg09G0058700 |
| flavonoid biosynthesis | F3'H | *L. megaphylla* | Limeg02G0090800,Limeg05G0275600,Limeg01G0077600,Limeg10G0083600,  Limeg10G0040700,Limeg11G0016600,Limeg05G0225400,Limeg01G0292700,  Limeg11G0016800 |
| flavonoid biosynthesis | F3'5'H | *L. megaphylla* | Limeg03G0232700,Limeg06G0205600,Limeg10G0083800,LimegUnG0050400,  Limeg06G0206100,Limeg09G0024900 |
| flavonoid biosynthesis | DFR | *L. megaphylla* | Limeg06G0030900,Limeg12G0006200 |
| flavonoid biosynthesis | ANS | *L. megaphylla* | Limeg02G0017500,Limeg02G0017600,Limeg02G0023100,Limeg10G0144200,  Limeg11G0046200 |
| lignin biosynthesis | C3H | *L. megaphylla* | Limeg03G0276700,Limeg09G0005900 |
| lignin biosynthesis | HCT | *L. megaphylla* | Limeg01G0122700,Limeg02G0170900,Limeg04G0251400,Limeg05G0001900,  Limeg05G0263200,Limeg07G0014000,Limeg08G0038400,Limeg08G0038800,  Limeg08G0038900,Limeg08G0039000,Limeg08G0039800,Limeg08G0047400,  Limeg08G0047500,Limeg08G0048200,Limeg08G0048300,Limeg08G0048700,  Limeg08G0049000,Limeg08G0049300,LimegUnG0067300,LimegUnG0067500 |
| lignin biosynthesis | CCoAOMT | *L. megaphylla* | Limeg03G0063000,Limeg04G0283100,Limeg04G0341500,Limeg05G0130100,  Limeg05G0130200,Limeg05G0130300 |
| lignin biosynthesis | CCR | *L. megaphylla* | Limeg01G0155000,Limeg01G0155100,Limeg01G0187700,Limeg01G0312400,  Limeg01G0312500,Limeg01G0369000,Limeg01G0369200,Limeg01G0369300,  Limeg01G0369400,Limeg01G0369500,Limeg01G0369600,Limeg02G0027400,  Limeg02G0027600,Limeg03G0012000,Limeg04G0031700,Limeg04G0031900,  Limeg04G0213200,Limeg06G0188800,Limeg07G0168400,Limeg07G0168500,  Limeg08G0174500,Limeg10G0014700,Limeg11G0189600 |
| lignin biosynthesis | F5H | *L. megaphylla* | Limeg02G0299300,Limeg06G0032300,Limeg06G0032600,Limeg10G0083700 |
| lignin biosynthesis | COMT | *L. megaphylla* | Limeg02G0084800,Limeg03G0192700,Limeg03G0192900,Limeg03G0210100,  Limeg07G0034800,Limeg07G0034900,Limeg07G0035000,Limeg09G0032800,  Limeg11G0108100,Limeg11G0108200,Limeg11G0108500 |
| lignin biosynthesis | CAD | *L. megaphylla* | Limeg01G0125000,Limeg01G0125300,Limeg01G0125400,Limeg01G0125600,  Limeg01G0126000,Limeg01G0126300,Limeg02G0015200,Limeg04G0333300,  Limeg04G0333500,Limeg05G0156300,Limeg05G0212200,Limeg11G0162400,  LimegUnG0019200,LimegUnG0043100,LimegUnG0079300 |
| General phenylpropanoid biosynthesis | PAL | *C. kanehirae* | CKAN_02604300,CKAN_00809200,CKAN_01274900,CKAN_02211300 |
| General phenylpropanoid biosynthesis | C4H | *C. kanehirae* | CKAN_00761900,CKAN_01989000,CKAN_01991300,CKAN_01992800,  CKAN_02528100,CKAN_02528500,CKAN_02528600 |
| General phenylpropanoid biosynthesis | 4CL | *C. kanehirae* | CKAN_00728200,CKAN_00728400,CKAN_00728500,CKAN_00728700,  CKAN_00971400,CKAN_01181000,CKAN_01343200,CKAN_01623400,  CKAN_01960000,CKAN_02212400,CKAN_02219900 |
| flavonoid biosynthesis | CHS | *C. kanehirae* | CKAN_00275500,CKAN_00275700,CKAN_01802600,CKAN_01802700,  CKAN_02543700,CKAN_02671900 |
| flavonoid biosynthesis | CHI | *C. kanehirae* | CKAN_00089600,CKAN_00532200,CKAN_00912300,CKAN_02525300,  CKAN_02550500 |
| flavonoid biosynthesis | F3H | *C. kanehirae* | CKAN_01289000,CKAN_01542900,CKAN_00248800,CKAN_01542500,  CKAN_00248700,CKAN_01126600,CKAN_00910700,CKAN_01756900,  CKAN_02311500,CKAN_01920000,CKAN_01667200,CKAN_01626900,  CKAN_02006500,CKAN_01667100,CKAN_01542400,CKAN_01542600,  CKAN_01483000,CKAN_02731200,CKAN_01667300,CKAN_02370700,  CKAN_01859600,CKAN_00499400,CKAN_01903800,CKAN_00950800,  CKAN_00777600 |
| flavonoid biosynthesis | FLS | *C. kanehirae* | CKAN_02081600,CKAN_01920000,CKAN_01115500,CKAN_02179200,C  KAN_00093500 |
| flavonoid biosynthesis | F3'H | *C. kanehirae* | CKAN_01289000,CKAN_01542900,CKAN_00248800,CKAN_01542500,  CKAN_00248700,CKAN_01126600,CKAN_00910700,CKAN_01756900,  CKAN_02311500,CKAN_01920000,CKAN_01667200,CKAN_01626900,  CKAN_02006500,CKAN_01667100,CKAN_01542400,CKAN_01542600,  CKAN_01483000,CKAN_02731200,CKAN_01667300,CKAN_02370700,  CKAN_01859600,CKAN_00499400,CKAN_01903800,CKAN_00950800,  CKAN_00777600 |
| flavonoid biosynthesis | F3'5'H | *C. kanehirae* | CKAN_01346300,CKAN_02210900,CKAN_02524600,CKAN_00858500,  CKAN_01346000,CKAN_0260870 |
| flavonoid biosynthesis | DFR | *C. kanehirae* | CKAN_02241700,CKAN_02241800,CKAN_01479400,CKAN_02288700,  CKAN_00933800,CKAN_01042500,CKAN_02241900 |
| flavonoid biosynthesis | ANS | *C. kanehirae* | CKAN_02558900,CKAN_02006400,CKAN_01002600,CKAN_02496600,  CKAN_01006400 |
| lignin biosynthesis | C3H | *C. kanehirae* | CKAN_01892100,CKAN_02230700,CKAN_02230900 |
| lignin biosynthesis | HCT | *C. kanehirae* | CKAN_00066200,CKAN_00066300,CKAN_00243700,CKAN_00508700,  CKAN_00881500,CKAN_01877200,CKAN_02031700,CKAN_02031800,  CKAN_02031900,CKAN_02032000,CKAN_02032300,CKAN_02032800,  CKAN_02040700,CKAN_02040800,CKAN_02041400,CKAN_02041500 |
| lignin biosynthesis | CCoAOMT | *C. kanehirae* | CKAN_00606200,CKAN_00663300,CKAN_00663500,CKAN_00859200,  CKAN_01146900,CKAN_01147100,CKAN_01670800 |
| lignin biosynthesis | CCR | *C. kanehirae* | CKAN_00049000,CKAN_00049100,CKAN_00049200,CKAN_00049300,  CKAN_00049400,CKAN_00049500,CKAN_00098500,CKAN_00098600,  CKAN_00098700,CKAN_00213700,CKAN_00384100,CKAN_00384400,  CKAN_00540800,CKAN_00999300,CKAN_00999500,CKAN_01355700,  CKAN_01935700 |
| lignin biosynthesis | F5H | *C. kanehirae* | CKAN_00772700,CKAN_01271900,CKAN_01478200,CKAN_01478500,  CKAN_01478700,CKAN_02608800,CKAN_02609000 |
| lignin biosynthesis | COMT | *C. kanehirae* | CKAN_00235500,CKAN_00950600,CKAN_00950900,CKAN_00951000,  CKAN_00951100,CKAN_00951200,CKAN_00951300,CKAN_00951400,  CKAN_00951500,CKAN_00951600,CKAN_01512900,CKAN_01542800,  CKAN_01543200,CKAN_01543400,CKAN_01543500,CKAN_01543700,  CKAN_01543800,CKAN_01543900,CKAN_01859000,CKAN_01859100,  CKAN_01859200,CKAN_01859500 |
| lignin biosynthesis | CAD | *C. kanehirae* | CKAN_00240600,CKAN_00240700,CKAN_00240800,CKAN_00656000,  CKAN_00656300,CKAN_01171800,CKAN_01220800,CKAN_02303500,  CKAN_02407900,CKAN_02408000,CKAN_02408100 |
| General phenylpropanoid biosynthesis | PAL | *L. cubeba* | Lcu05G_15240,Lcu04G_12697,Lcu05G_15239,Lcu09G_25299,Lcu11G_27950 |
| General phenylpropanoid biosynthesis | C4H | *L. cubeba* | Lcu04G_13188,Lcu07G_21838,Lcu07G_21839,Lcu07G_21843 |
| General phenylpropanoid biosynthesis | 4CL | *L. cubeba* | Lcu02G_08012,Lcu03G_08858,Lcu04G_13515,Lcu04G_13517,Lcu04G_14101,  Lcu04G_14794,Lcu06G_18405,Lcu06G_19527,Lcu06G_19538,Lcu06G_19539,  Lcu11G_27869 |
| flavonoid biosynthesis | CHS | *L. cubeba* | Lcu01G_03002,Lcu05G_15108,Lcu08G_23607,Lcu08G_23608,Lcu09G_24673 |
| flavonoid biosynthesis | CHI | *L. cubeba* | Lcu01G_02298,Lcu02G_05610,Lcu02G_05611,Lcu04G_11411,Lcu07G_21874,  Lcu09G_24751,Lcu12G_29511 |
| flavonoid biosynthesis | F3H | *L. cubeba* | Lcu04G_13048,Lcu10G_26763,Lcu02G_06918,Lcu03G_11114,Lcu03G_11113,  Lcu08G_23034,Lcu03G_09828,Lcu06G_19044,Lcu06G_20139,Lcu08G_22787,  Lcu08G_22786,Lcu06G_18901,Lcu03G_10479,Lcu08G_23251,Lcu03G_09594,  Lcu02G_07563,Lcu04G_13872,Lcu03G_09819,Lcu03G_09818,Lcu03G_09811,  Lcu03G_09817,Lcu04G_11572,Lcu06G_20138,Lcu06G_18589,Lcu02G_07467,  Lcu03G_08823 |
| flavonoid biosynthesis | FLS | *L. cubeba* | Lcu05G_17339,Lcu06G_19044,Lcu01G_00828,Lcu02G_07564,Lcu11G_28296 |
| flavonoid biosynthesis | F3'H | *L. cubeba* | Lcu04G_13048,Lcu10G_26763,Lcu02G_06918,Lcu03G_11114,Lcu03G_11113,  Lcu08G_23034,Lcu03G_09828,Lcu06G_19044,Lcu06G_20139,Lcu08G_22787,  Lcu08G_22786,Lcu06G_18901,Lcu03G_10479,Lcu08G_23251,Lcu03G_09594,  Lcu02G_07563,Lcu04G_13872,Lcu03G_09819,Lcu03G_09818,Lcu03G_09811,  Lcu03G_09817,Lcu04G_11572,Lcu06G_20138,Lcu06G_18589,Lcu02G_07467,  Lcu03G_08823 |
| flavonoid biosynthesis | F3'5'H | *L. cubeba* | Lcu11G_29056,Lcu11G_29055,Lcu02G_05013,Lcu03G_09273,Lcu03G_09274,  Lcu09G_25251,Lcu09G_25250,Lcu07G_21885,Lcu11G_27954 |
| flavonoid biosynthesis | DFR | *L. cubeba* | Lcu01G_00898,Lcu02G_06951,Lcu05G_16686,Lcu05G_16688,Lcu07G_21223,  Lcu10G_26990,Lcu10G_27392,Lcu10G_27394 |
| flavonoid biosynthesis | ANS | *L. cubeba* | Lcu04G_14475,Lcu07G_22187,Lcu09G_24845,Lcu04G_14431 |
| lignin biosynthesis | C3H | *L. cubeba* | Lcu06G_18776,Lcu11G_27761,Lcu11G_27767 |
| lignin biosynthesis | HCT | *L. cubeba* | Lcu01G_02735,Lcu02G_04290,Lcu02G_04934,Lcu06G_20265,Lcu06G_20271,  Lcu06G_20274,Lcu06G_20276,Lcu06G_20363,Lcu06G_20364,Lcu06G_20366,  Lcu10G_27548 |
| lignin biosynthesis | CCoAOMT | *L. cubeba* | Lcu02G_04824,Lcu06G_19936,Lcu06G_19949 |
| lignin biosynthesis | CCR | *L. cubeba* | Lcu01G_00464,Lcu01G_00465,Lcu01G_00466,Lcu01G_00897,Lcu01G_00899,  Lcu01G_02041,Lcu02G_04008,Lcu02G_04010,Lcu02G_04720,Lcu02G_05540,  Lcu03G_08837,Lcu04G_11929,Lcu04G_13681,Lcu04G_14400,Lcu05G_18243,  Lcu06G_19263,Lcu06G_19544,Lcu09G_25985 |
| lignin biosynthesis | F5H | *L. cubeba* | Lcu02G_06960,Lcu02G_06964,Lcu04G_13097,Lcu08G_23537 |
| lignin biosynthesis | COMT | *L. cubeba* | Lcu01G_00700,Lcu02G_06792,Lcu03G_09829,Lcu03G_09830,Lcu04G_13873,  Lcu04G_13905,Lcu07G_20730,Lcu07G_20731,Lcu07G_20734,Lcu08G_23250,  Lcu08G_24363,Lcu08G_24364,Lcu12G_30118,Lcu12G_30119,Lcu12G_30120 |
| lignin biosynthesis | CAD | *L. cubeba* | Lcu01G_02545,Lcu01G_02546,Lcu01G_02548,Lcu01G_02550,Lcu02G_06692,  Lcu04G_11528,Lcu04G_11530,Lcu05G_15871,Lcu05G_16681,Lcu06G_18486,  Lcu07G_21198,Lcu10G_26844 |
| General phenylpropanoid biosynthesis | PAL | *P. bournei* | Maker00018015,Maker00029283,Maker00017961,Maker00051382,Maker00029233,  Maker00042631 |
| General phenylpropanoid biosynthesis | C4H | *P. bournei* | Maker00033450,Maker00033478,Maker00034763 |
| General phenylpropanoid biosynthesis | 4CL | *P. bournei* | Maker00001273,Maker00003155,Maker00007568,Maker00009753,Maker00023519,  Maker00025765,Maker00029897,Maker00031597,Maker00055329 |
| flavonoid biosynthesis | CHS | *P. bournei* | . |
| flavonoid biosynthesis | CHI | *P. bournei* | Maker00033396,Maker00046330 |
| flavonoid biosynthesis | F3H | *P. bournei* | Maker00028418,Maker00054773,Maker00038906,Maker00043950,Maker00032782,  Maker00034514,Maker00023523,Maker00037150,Maker00013995,Maker00034420,  Maker00032665,Maker00004286 |
| flavonoid biosynthesis | FLS | *P. bournei* | Maker00026127,Maker00015688,Maker00044968,Maker00043950,Maker00006181 |
| flavonoid biosynthesis | F3'H | *P. bournei* | Maker00049000,Maker00033657,Maker00030298,Maker00033456,Maker00026740 |
| flavonoid biosynthesis | F3'5'H | *P. bournei* | . |
| flavonoid biosynthesis | DFR | *P. bournei* | Maker00016215,Maker00016801 |
| flavonoid biosynthesis | ANS | *P. bournei* | Maker00040105,Maker00027670,Maker00027401 |
| lignin biosynthesis | C3H | *P. bournei* | Maker00031478,Maker00031591 |
| lignin biosynthesis | HCT | *P. bournei* | Maker00004914,Maker00017783,Maker00021660,Maker00021767,Maker00043321,  Maker00046804,Maker00048170,Maker00051586,Maker00052055,Maker00052094,  Maker00052095,Maker00052098,Maker00052100 |
| lignin biosynthesis | CCoAOMT | *P. bournei* | Maker00010460,Maker00022157,Maker00024457,Maker00024481 |
| lignin biosynthesis | CCR | *P. bournei* | Maker00003053,Maker00005850,Maker00011058,Maker00011956,Maker00012462,  Maker00012468,Maker00012469,Maker00014049,Maker00021717,Maker00023346,  Maker00027758,Maker00028284,Maker00028452,Maker00034060,Maker00036546,  Maker00040395,Maker00040439,Maker00045294,Maker00045413,Maker00055525 |
| lignin biosynthesis | F5H | *P. bournei* | Maker00036411 |
| lignin biosynthesis | COMT | *P. bournei* | Maker00031901,Maker00033335,Maker00033354,Maker00033356,Maker00033369,  Maker00033380,Maker00034351,Maker00034368,Maker00041925,Maker00043699,  Maker00043865,Maker00043874,Maker00043875,Maker00043886,Maker00049298 |
| lignin biosynthesis | CAD | *P. bournei* | Maker00014940,Maker00020743,Maker00038119,Maker00043745,Maker00048770,  Maker00053108 |

**Table S15.** Gene annotation of the general phenylpropanoid, flavonoid and lignin biosynthesis pathways in four Lauraceae species.

| **Enzyme** | **TDPD** | ***A. trichopoda*** | ***N. colorata*** | ***P. nigrum*** | ***L. chinense*** | ***C. kanehirae*** | ***L. megaphylla*** | ***L. cubeba*** | ***A. coerulea*** |
| --- | --- | --- | --- | --- | --- | --- | --- | --- | --- |
| **4CL** | **All** | 6 | 10 | 26 | 17 | 11 | 12 | 11 | 11 |
|  | **TD** | 0 (0.00%) | 3 (30.00%) | 4 (15.38%) | 0 (0.00%) | 2 (18.18%) | 4 (33.33%) | 2 (18.18%) | 4 (36.36%) |
|  | **PD** | 0 (0.00%) | 1 (10.00%) | 2 (7.69%) | 4 (23.53%) | 2 (18.18%) | 3 (25.00%) | 2 (18.18%) | 0 (0.00%) |
|  | **TDPD** | (0.00%)0 | 4 (40.00%) | 6 (23.08%) | 4 (23.53%) | 4 (36.36%) | 7 (58.33%) | 4 (36.36%) | 4 (36.36%) |
| **4CH** | **All** | 2 | 2 | 5 | 3 | 7 | 4 | 4 | 1 |
|  | **TD** | 0 (0.00%) | 0 (0.00%) | 2 (40.00%) | 0 (0.00%) | 2 (28.57%) | 2 (50.00%) | 2 (50.00%) | 0 (0.00%) |
|  | **PD** | 0 (0.00%) | 0 (0.00%) | 0 (0.00%) | 0 (0.00%) | 1 (14.29%) | 1 (25.00%) | 1 (25.00%) | 0 (0.00%) |
|  | **TDPD** | 0 (0.00%) | 0 (0.00%) | 2 (40.00%) | 0 (0.00%) | 3 (42.86%) | 3 (75.00%) | 3 (75.00%) | 0 (0.00%) |

| **Enzyme** | **TDPD** | ***P. trichocarpa*** | ***A. thaliana*** | ***C. cajan*** | ***S. lycopersicum*** | ***C. canephora*** | ***O. sativa*** | ***S. bicolor*** | ***M. acuminata*** | ***Z. marina*** |
| --- | --- | --- | --- | --- | --- | --- | --- | --- | --- | --- |
| **4CL** | **All** | 9 | 10 | 16 | 23 | 8 | 12 | 15 | 15 | 9 |
|  | **TD** | 0 (0.00%) | 4 (40.00%) | 0 (0.00%) | 9 (39.13%) | 2 (25.00%) | 2 (16.67%) | 2 (13.33%) | 0 (0.00%) | 0 (0.00%) |
|  | **PD** | 0 (0.00%) | 2 (20.00%) | 6 (37.50%) | 2 (8.70%) | 0 (0.00%) | 0 (0.00%) | 3 (20.00%) | 2 (13.33%) | 0 (0.00%) |
|  | **TDPD** | 0 (0.00%) | 6 (60.00%) | 6 (37.50%) | 11 (47.83%) | 2 (25.00%) | 2 (16.67%) | 5 (33.33%) | 2 (13.33%) | 0 (0.00%) |
| **4CH** | **All** | 4 | 1 | 3 | 0 | 4 | 4 | 3 | 9 | 4 |
|  | **TD** | 0 (0.00%) | 0 (0.00%) | 0 (0.00%) | 0 (0.00%) | 0 (0.00%) | 0 (0.00%) | 0 (0.00%) | 3 (33.33%) | 0 (0.00%) |
|  | **PD** | 0 (0.00%) | 0 (0.00%) | 0 (0.00%) | 0 (0.00%) | 0 (0.00%) | 2 (50.00%) | 0 (0.00%) | 0 (0.00%) | 0 (0.00%) |
|  | **TDPD** | 0 (0.00%) | 0 (0.00%) | 0 (0.00%) | 0 (0.00%) | 0 (0.00%) | 2 (50.00%) | 0 (0.00%) | 3 (33.33%) | 0 (0.00%) |

**Table S16.** Number and proportion of tandem (TD) duplicated or proximal duplicated (PD) genes of 4CL and 4CH gene families in general phenylpropanoid pathways. All, all genes of the relevant gene families in general phenylpropanoid pathways.

| **Enzyme** | **TDPD** | ***A. trichopoda*** | ***N. colorata*** | ***P. nigrum*** | ***L. chinense*** | ***C. kanehirae*** | ***L. megaphylla*** | ***L. cubeba*** | ***A. coerulea*** |
| --- | --- | --- | --- | --- | --- | --- | --- | --- | --- |
| **F3H** | **All** | 22 | 20 | 32 | 30 | 25 | 33 | 27 | 14 |
|  | **TD** | 7 (31.82%) | 9 (45.00%) | 4 (12.50%) | 14 (46.67%) | 8 (32.00%) | 12 (36.36%) | 9 (33.33%) | 7 (50.00%) |
|  | **PD** | 4 (18.18%) | 2 (10.00%) | 5 (15.63%) | 6 (20.00%) | 1 (4.00%) | 6 (18.18%) | 2 (7.41%) | 0 (0.00%) |
|  | **TDPD** | 10 (45.45%) | 11 (55.00%) | 9 (28.13%) | 20 (66.67%) | 9 (36.00%) | 18 (54.55%) | 11 (40.74%) | 7 (50.00%) |
| **F3‘5’H** | **All** | 2 | 7 | 4 | 4 | 6 | 6 | 10 | 6 |
|  | **TD** | 0 (0.00%) | 3 (42.86%) | 0 (0.00%) | 2 (50.00%) | 0 (0.00%) | 0 (0.00%) | 6 (60.00%) | 0 (0.00%) |
|  | **PD** | 0 (0.00%) | 1 (14.29%) | 0 (0.00%) | 0 (0.00%) | 2 (33.33%) | 2 (33.33%) | 0 (0.00%) | 0 (0.00%) |
|  | **TDPD** | 0 (0.00%) | 4 (57.14%) | 0 (0.00%) | 2 (50.00%) | 2 (33.33%) | 2 (33.33%) | 6 (60.00%） | 0 (0.00%) |

| **Enzyme** | **TDPD** | ***P. trichocarpa*** | ***A. thaliana*** | ***C. cajan*** | ***S. lycopersicum*** | ***C. canephora*** | ***O. sativa*** | ***S. bicolor*** | ***M. acuminata*** | ***Z. marina*** |
| --- | --- | --- | --- | --- | --- | --- | --- | --- | --- | --- |
| **F3H** | **All** | 38 | 10 | 19 | 18 | 15 | 16 | 19 | 25 | 11 |
|  | **TD** | 15 (39.47%) | 2 (20.00%) | 3 (15.79%) | 5 (27.78%) | 4 (26.67%) | 2 (12.50%) | 6 (31.58%) | 2 (8.00%) | 0 (0.00%) |
|  | **PD** | 5 (13.16%) | 0 (0.00%) | 1 (5.26%) | 1 (5.56%) | 1 (6.67%) | 4 (25.00%) | 4 (21.05%) | 0 (0.00%) | 2 (18.18%) |
|  | **TDPD** | 20 (52.63%) | 2 (20.00%) | 4 (21.05%) | 6 (33.33%) | 5 (33.33%) | 6 (37.50%) | 10 (52.63%) | 2 (8.00%) | 2 (18.18%) |
| **F3‘5’H** | **All** | 10 | 0 | 5 | 2 | 5 | 5 | 7 | 10 | 0 |
|  | **TD** | 0 (0.00%) | 0 (0.00%) | 0 (0.00%) | 0 (0.00%) | 4 (80.00%) | 0 (0.00%) | 4 (57.14%) | 3 (30.00%) | 0 (0.00%) |
|  | **PD** | 7 (70.00%) | 0 (0.00%) | 0 (0.00%) | 0 (0.00%) | 0 (0.00%) | 0 (0.00%) | 2 (28.57%) | 1 (10.00%) | 0 (0.00%) |
|  | **TDPD** | 7 (70.00%) | 0 (0.00%) | 0 (0.00%) | 0 (0.00%) | 4 (80.00%) | 0 (0.00%) | 6 (85.71%) | 4 (40.00%) | 0 (0.00%) |

**Table S17.** Number and proportion of tandem (TD) duplicated or proximal duplicated (PD) genes of F3H and F3'5'H gene families in flavonoid biosynthesis pathways. All, all genes of the relevant gene families in flavonoid biosynthetic pathway.

| **Enzyme** | **TDPD** | ***A. trichopoda*** | ***N. colorata*** | ***P. nigrum*** | ***L. chinense*** | ***C. kanehirae*** | ***L. megaphylla*** | ***L. cubeba*** | ***A. coerulea*** |
| --- | --- | --- | --- | --- | --- | --- | --- | --- | --- |
| **C3H** | **All** | 1 | 1 | 5 | 6 | 3 | 2 | 3 | 2 |
|  | **TD** | 0 (0.00%) | 0 (0.00%) | 0 (0.00%) | 2 (33.33%) | 0 (0.00%) | 0 (0.00%) | 0 (0.00%) | 0 (0.00%) |
|  | **PD** | 0 (0.00%) | 0 (0.00%) | 0 (0.00%) | 0 (0.00%) | 2 (66.67%) | 0 (0.00%) | 2 (66.67%) | 0 (0.00%) |
|  | **TDPD** | 0 (0.00%) | 0 (0.00%) | 0 (0.00% | 2 (33.33%) | 2 (66.67%) | 0 (0.00%) | 2 (66.67%) | 0 (0.00%) |
| **CAD** | **All** | 9 | 2 | 10 | 22 | 11 | 15 | 12 | 13 |
|  | **TD** | 5 (55.56%) | 0 (0.00%) | 5 (50.00%) | 16 (72.73%) | 6 (54.55%) | 2 (13.33%) | 2 (16.67%) | 7 (53.85%) |
|  | **PD** | 0 (0.00%) | 0 (0.00%) | 0 (0.00%) | 5 (22.73%) | 2 (18.18%) | 6 (40.00%) | 4 (33.33%) | 2 (15.38%) |
|  | **TDPD** | 5 (55.56%) | 0 (0.00%) | 5 (50.00%) | 21 (95.45%) | 8 (72.73%) | 8 (53.33%) | 6 (50.00%) | 9 (69.23%) |
| **CCoAOMT** | **All** | 2 | 6 | 21 | 9 | 7 | 6 | 3 | 3 |
|  | **TD** | 0 (0.00%) | 0 (0.00%) | 10 (47.62%) | 3 (33.33%) | 2 (28.57%) | 3 (50.00%) | 0 (0.00%) | 2 (66.67%) |
|  | **PD** | 0 (0.00%) | 2 (33.33%) | 7 (33.33%) | 4 (44.44%) | 3 (42.86%) | 0 (0.00%) | 0 (0.00%) | 0 (0.00%) |
|  | **TDPD** | 0 (0.00%) | 2 (33.33%) | 17 (80.95%) | 7 (77.78%) | 5 (71.43%) | 3 (50.00%) | 0 (0.00%) | 2 (66.67%) |
| **CCR** | **All** | 14 | 9 | 25 | 18 | 17 | 23 | 18 | 14 |
|  | **TD** | 7 (50.00%) | 3 (33.33%) | 8 (32.00%) | 8 (44.44%) | 9 (52.94%) | 11 (47.83%) | 3 (16.67%) | 7 (50.00%) |
|  | **PD** | 0 (0.00%) | 2 (22.22%) | 5 (20.00%) | 2 (11.11%) | 4 (23.53%) | 5 (21.74%) | 4 (22.22%) | 0 (0.00%) |
|  | **TDPD** | 7 (50.00%) | 5 (55.56%) | 13 (52.00%) | 10 (55.56%) | 13 (76.47%) | 16 (69.57%) | 7 (38.89%) | 7 (50.00%) |
| **COMT** | **All** | 1 | 12 | 25 | 21 | 22 | 11 | 15 | 25 |
|  | **TD** | 0 (0.00%) | 5 (41.67%) | 15 (60.00%) | 14 (66.67%) | 16 (72.73%) | 5 (45.45%) | 9 (60.00%) | 8 (32.00%) |
|  | **PD** | 0 (0.00%) | 6 (50.00%) | 6 (24.00%) | 5 (23.81%) | 4 (18.18%) | 3 (27.27%) | 1 (6.67%) | 9 (36.00%) |
|  | **TDPD** | 0 (0.00%) | 11 (91.67%) | 21 (84.00%) | 19 (90.48%) | 20 (90.91%) | 8 (72.73%) | 10 (66.67%) | 17 (68.00%) |
| **F5H** | **All** | 2 | 4 | 10 | 14 | 7 | 4 | 4 | 16 |
|  | **TD** | 2 (100.00%) | 0 (0.00%) | 2 (20.00%) | 2 (14.29%) | 2 (28.57%) | 0 (0.00%) | 0 (0.00%) | 9 (56.25%) |
|  | **PD** | 0 (0.00%) | 0 (0.00%) | 4 (40.00%) | 8 (57.14%) | 3 (42.86%) | 2 (50.00%) | 2 (50.00%) | 0 (0.00%) |
|  | **TDPD** | 2 (100.) | 0 (0.00%) | 6 (60.00%) | 10 (71.43%) | 5 (71.43%) | 2 (50.00%) | 2 (50.00%) | 9 (56.25%) |
| **HCT** | **All** | 9 | 14 | 22 | 13 | 16 | 20 | 11 | 17 |
|  | **TD** | 4 (44.44%) | 6 (42.86%) | 8 (36.36%) | 4 (30.77%) | 10 (62.50%) | 7 (35.00%) | 2 (18.18%) | 4 (23.53%) |
|  | **PD** | 0 (0.00%) | 1 (7.14%) | 12 (54.55%) | 0 (0.00%) | 2 (12.50%) | 7 (35.00%) | 5 (45.45%) | 2 (11.76%) |
|  | **TDPD** | 4 (44.44%) | 7 (50.00%) | 20 (90.91%) | 4 (30.77%) | 12 (75.00%) | 14 (70.00%) | 7 (63.64%) | 6 (35.29%) |

| **Enzyme** | **TDPD** | ***P. trichocarpa*** | ***A. thaliana*** | ***C. cajan*** | ***S. lycopersicum*** | ***C. canephora*** | ***O. sativa*** | ***S. bicolor*** | ***M. acuminata*** | ***Z. marina*** |
| --- | --- | --- | --- | --- | --- | --- | --- | --- | --- | --- |
| **C3H** | **All** | 0 | 1 | 1 | 1 | 2 | 3 | 3 | 1 | 0 |
|  | **TD** | 0 (0.00%) | 0 (0.00%) | 0 (0.00%) | 0 (0.00%) | 0 (0.00%) | 0 (0.00%) | 0 (0.00%) | 0 (0.00%) | 0 (0.00%) |
|  | **PD** | 0 (0.00%) | 0 (0.00%) | 0 (0.00%) | 0 (0.00%) | 0 (0.00%) | 2 (66.67%) | 0 (0.00%) | 0 (0.00%) | 0 (0.00%) |
|  | **TDPD** | 0 (0.00%) | 0 (0.00%) | 0 (0.00%) | 0 (0.00%) | 0 (0.00%) | 2 (66.67%) | 0 (0.00%) | 0 (0.00%) | 0 (0.00%) |
| **CAD** | **All** | 20 | 13 | 10 | 9 | 30 | 12 | 12 | 9 | 4 |
|  | **TD** | 8 (40.00%) | 5 (38.46%) | 3 (30..00%) | 6 (66.67%) | 13 (43.33%) | 4 (33.33%) | 7 (58.33%) | 2 (22.22%) | 0 (0.00%) |
|  | **PD** | 1 (5.00%) | 0 (0.00%) | 0 (0.00%) | 0 (0.00%) | 6 (20.00%) | 0 (0.00%) | 1 (8.33%) | 0 (0.00%) | 0 (0.00%) |
|  | **TDPD** | 9 (45.00%) | 5 (38.46%) | 3 (30.00%) | 6 (66.67%) | 19 (63.33%) | 4 (33.33% | 8 (66.67%) | 2 (22.22%) | 0 (0.00%) |
| **CCoAOMT** | **All** | 7 | 6 | 8 | 11 | 5 | 5 | 6 | 9 | 4 |
|  | **TD** | 2 (28.57%) | 4 (66.67%) | 0 (0.00%) | 3 (27.27%) | 0 (0.00%) | 0 (0.00%) | 2 (33.33%) | 2 (22.22%) | 0 (0.00%) |
|  | **PD** | 0 (0.00%) | 0 (0.00%) | 0 (0.00%) | 1 (9.09%) | 0 (0.00%) | 2 (40.00%) | 0 (0.00%) | 0 (0.00%) | 0 (0.00%) |
|  | **TDPD** | 2 (28.57%) | 4 (66.67%) | 0 (0.00%) | 4 (36.36%) | 0 (0.00%) | 2 (40.00%) | 2 (33.33%) | 2 (22.22%) | 0 (0.00%) |
| **CCR** | **All** | 27 | 10 | 10 | 12 | 12 | 34 | 32 | 15 | 12 |
|  | **TD** | 9 (33.33%) | 2 (20.00%) | 0 (0.00%) | 2 (16.67%) | 2 (16.67%) | 6 (17.65%) | 17 (53.13%) | 0 (0.00%) | 2 (16.67%) |
|  | **PD** | 5 (18.52%) | 0 (0.00%) | 4 (40.00%) | 0 (0.00%) | 1 (8.33%) | 13 (38.24%) | 0 (0.00%) | 0 (0.00%) | 3 (25.00%) |
|  | **TDPD** | 14 (51.84%) | 2 (20.00%) | 4 (40.00%) | 2 (16.67%) | 3 (25.00%) | 19 (55.88%) | 0 (0.00%) | 0 (0.00%) | 5 (41.67%) |
| **COMT** | **All** | 9 | 3 | 3 | 3 | 15 | 0 | 3 | 34 | 19 |
|  | **TD** | 2 (22.22%) | 0 (0.00%) | 0 (0.00%) | 0 (0.00%) | 3 (20.00%) | 0 (0.00%) | 0 (0.00%) | 22 (64.71%) | 2 (10.53%) |
|  | **PD** | 0 (0.00%) | 0 (0.00%) | 0 (0.00%) | 0 (0.00%) | 4 (26.67%) | 0 (0.00%) | 0 (0.00%) | 7 (20.59%) | 3 (15.79%) |
|  | **TDPD** | 2 (22.22%) | 0 (0.00%) | 0 (0.00%) | 0 (0.00%) | 7 (46.67%) | 0 (0.00%) | 0 (0.00%) | 29 (85.29%) | 5 (26.32%) |
| **F5H** | **All** | 8 | 2 | 7 | 8 | 8 | 4 | 3 | 5 | 2 |
|  | **TD** | 1 (12.50%) | 0 (0.00%) | 3 (42.86%) | 1 (12.50%) | 3 (37.50%) | 0 (0.00%) | 0 (0.00%) | 1 (20.00%) | 0 (0.00%) |
|  | **PD** | 3 (37.50%) | 0 (0.00%) | 1 (41.29%) | 1 (12.50%) | 2 (25.00%) | 0 (0.00%) | 0 (0.00%) | 0 (0.00%) | 0 (0.00%) |
|  | **TDPD** | 4 (50.00%) | 0 (0.00%) | 4 (57.14%) | 2 (25.00%) | 5 (62.50%) | 0 (0.00%) | 0 (0.00%) | 1 (20.00%) | 0 (0.00%) |
| **HCT** | **All** | 13 | 13 | 18 | 10 | 10 | 11 | 23 | 5 | 10 |
|  | **TD** | 4 (30.77%) | 2 (15.38%) | 2 (11.11%) | 4 (40.00%) | 2 (20.00%) | 2 (18.18%) | 4 (17.39%) | 0 (0.00%) | 3 (30.00%) |
|  | **PD** | 0 (0.00%) | 4 (30.77%) | 7 (38.89%) | 1 (10.00%) | 0 (0.00%) | 3 (27.27%) | 7 (30.43%) | 0 (0.00%) | 2 (20.00%) |
|  | **TDPD** | 4 (30.77%) | 6 (46.15%) | 9 (50.00%) | 5 (50.00%) | 2 (20.00%) | 5 (45.45%) | 11 (47.83%) | 0 (0.00%) | 5 (50.00%) |

**Table S18.** Number and proportion of tandem (TD) duplicated or proximal duplicated (PD) genes in lignin biosynthesis pathways. All, all genes of the relevant gene families in lignin biosynthetic pathway.

|  | ***A. trichopoda*** | ***N. colorata*** | ***Z. marina*** | ***O. sativa*** | ***S. bicolor*** | ***M. acuminata*** | ***P. nigrum*** | ***L. chinense*** |
| --- | --- | --- | --- | --- | --- | --- | --- | --- |
| **TD** | 13 (34.21%) | 24 (32.43%) | 0 (0.00%) | 2 (22.22%) | 9 (50.00%) | 35 (31.25%) | 39 (15.29%) | 32 (39.02%) |
| **PD** | 10 (26.32%) | 18 (24.32%) | 0 (0.00%) | 1 (11.11%) | 1 (5.56%) | 39 (34.82%) | 67 (26.27%) | 22 (26.83%) |
| **TD\|PD** | 23 (60.53%) | 42 (56.76%) | 0 (0.00%) | 3 (33.33%) | 10 (55.56%) | 74 (66.07%) | 106 (41.57%) | 54 (65.85%) |
| **ALL** | 38 | 74 | 4 | 9 | 18 | 112 | 255 | 82 |

|  | ***L. cubeba*** | ***L. megaphylla*** | ***C. kanehirae*** | ***A. coerulea*** | ***S. lycopersicum*** | ***C. canephora*** | ***C. cajan*** | ***P. trichocarpa*** | ***A. thaliana*** |
| --- | --- | --- | --- | --- | --- | --- | --- | --- | --- |
| **TD** | 51 (57.30%) | 39 (50.65%) | 83 (63.85%) | 22 (29.33%) | 6 (21.43%) | 18 (41.86%) | 9 (23.08%) | 6 (15.00%) | 2 (33.33%) |
| **PD** | 18 (20.22%) | 27 (65.06%) | 23 (17.69%) | 25 (33.33%) | 5 (17.86%) | 5 (11.63%) | 9 (23.08%) | 13 (32.50%) | 0 (0.00%) |
| **TD\|PD** | 69 (77.53%) | 66 (85.71%) | 106 (81.54%) | 47 (62.67%) | 11 (39.29%) | 23 (53.49%) | 18 (46.15%) | 19 (47.50%) | 2 (33.33%) |
| **ALL** | 89 | 77 | 130 | 75 | 28 | 43 | 39 | 40 | 6 |

**Table S19.** Number and proportion of tandem (TD) duplicated or proximal duplicated (PD) genes of TPS (terpene synthases) genes. All, all the annotated TPS genes.
